# Financing transformative health systems towards achievement of the health Sustainable Development Goals: a model for projected resource needs in 67 low-income and middle-income countries

**DOI:** 10.1016/S2214-109X(17)30263-2

**Published:** 2017-07-17

**Authors:** Karin Stenberg, Odd Hanssen, Tessa Tan-Torres Edejer, Melanie Bertram, Callum Brindley, Andreia Meshreky, James E Rosen, John Stover, Paul Verboom, Rachel Sanders, Agnès Soucat

**Affiliations:** a Department of Health Systems Governance and Financing, WHO, Geneva, Switzerland; b 76, ch de Boissonnet, Lausanne, Switzerland; c 385 Chemin de L'Ovellas, 15 Les Collines de Pitegny, Gex, France; d Avenir Health, Glastonbury, CT, USA

## Abstract

**Background:**

The ambitious development agenda of the Sustainable Development Goals (SDGs) requires substantial investments across several sectors, including for SDG 3 (healthy lives and wellbeing). No estimates of the additional resources needed to strengthen comprehensive health service delivery towards the attainment of SDG 3 and universal health coverage in low-income and middle-income countries have been published.

**Methods:**

We developed a framework for health systems strengthening, within which population-level and individual-level health service coverage is gradually scaled up over time. We developed projections for 67 low-income and middle-income countries from 2016 to 2030, representing 95% of the total population in low-income and middle-income countries. We considered four service delivery platforms, and modelled two scenarios with differing levels of ambition: a progress scenario, in which countries' advancement towards global targets is constrained by their health system's assumed absorptive capacity, and an ambitious scenario, in which most countries attain the global targets. We estimated the associated costs and health effects, including reduced prevalence of illness, lives saved, and increases in life expectancy. We projected available funding by country and year, taking into account economic growth and anticipated allocation towards the health sector, to allow for an analysis of affordability and financial sustainability.

**Findings:**

We estimate that an additional $274 billion spending on health is needed per year by 2030 to make progress towards the SDG 3 targets (progress scenario), whereas US$371 billion would be needed to reach health system targets in the ambitious scenario—the equivalent of an additional $41 (range 15–102) or $58 (22–167) per person, respectively, by the final years of scale-up. In the ambitious scenario, total health-care spending would increase to a population-weighted mean of $271 per person (range 74–984) across country contexts, and the share of gross domestic product spent on health would increase to a mean of 7·5% (2·1–20·5). Around 75% of costs are for health systems, with health workforce and infrastructure (including medical equipment) as the main cost drivers. Despite projected increases in health spending, a financing gap of $20–54 billion per year is projected. Should funds be made available and used as planned, the ambitious scenario would save 97 million lives and significantly increase life expectancy by 3·1–8·4 years, depending on the country profile.

**Interpretation:**

All countries will need to strengthen investments in health systems to expand service provision in order to reach SDG 3 health targets, but even the poorest can reach some level of universality. In view of anticipated resource constraints, each country will need to prioritise equitably, plan strategically, and cost realistically its own path towards SDG 3 and universal health coverage.

**Funding:**

WHO.

## Introduction

The Sustainable Development Goals (SDGs) were adopted by the UN General Assembly in September, 2015. They set the global direction for 17 development goals, one of which, SDG 3, focuses on health.^1^ The SDGs substantially broaden the development agenda beyond the Millennium Development Goals (MDGs), with an emphasis on country-level ownership and multisectoral investments and a focus on leaving no one behind. After two decades of mostly positive economic growth, the number of low-income countries that need external development assistance has been falling.^2^ The 2015 Addis Ababa Action Agenda calls for increased mobilisation of domestic resources to achieve the SDGs.^3^

**Research in context**

**Evidence before this study**

In 2009, WHO published estimates of resources needed by 2015 to strengthen health service delivery in low-income countries to achieve the Millennium Development Goals (MDGs). These estimates were presented through the High-level Taskforce on Innovative International Financing for Health Systems (HLTF). At the time, the average per-person need was estimated as an additional US$29 (US$ 2005) by 2015, equivalent to a total mean spending need of $54 across low-income countries, reflecting MDG-related service benchmarks for 49 countries. Others subsequently converted the $54 estimate to $86 (US$ 2012). Since the adoption of the Sustainable Development Goals (SDGs) in September, 2015, demand is growing for guidance on pathways and resources needed to achieve the health-related SDG targets. Previous attempts to project resource implications for countries adopting SDG 3 targets have drawn upon the HLTF 2009 estimates, because no updates have been published.

**Added value of this study**

In recognition of the need to update the previous estimates and provide a more comprehensive assessment, we modelled country-based projections of strengthening health systems efforts to achieve the dual goals of population health and financial protection. We drew upon available studies and sectoral price tags, best practice, and tools to run models for 67 low-income and middle-income countries to assess yearly resource needs from 2016 to 2030. We present projected costs and health effects, along with the estimated financing gap. To our knowledge, ours is the first study to present a combined analysis of system-wide strategies to address a wide range of SDG health indicators and the associated overall health effects as shown by projected gains in life expectancy and healthy years lived.

**Implications of all the available evidence**

We have developed models and tools that allow detailed analysis of resource needs to strengthen country health systems and expand service packages, and projection of the associated expected health benefits. Our results provide evidence about the probable cost drivers within countries seeking to expand their health service coverage and an indicative estimate of the additional resource need. These estimates can be used to inform global policy discussions around post-2015 investment strategies and the relative role of domestic versus external funding. Application of these methods and tools at the country level can guide national priority setting and resource allocation.

SDG 3—“Ensure healthy lives and promote well-being for all at all ages”—is a broad health goal, and calls for achieving universal health coverage (UHC), which is defined as access for all people and communities to services that they need without financial hardship.^1^ Many countries are still far from UHC as measured by an index of access to 16 essential services.^4^ Furthermore, 100 million people yearly are driven below the poverty line because of direct health payments.^5^ Moving towards UHC entails adopting principles of progressive universalism, whereby equitable access to a set of key health services increases with time, starting with the poorest. The service package provided is successively expanded, and an increasing share of costs is covered through pooled funding, thereby reducing reliance on out-of-pocket payments. The intersectoral links between the SDGs are crucial, because many goals represent sectors that are essential to address the environmental and social determinants of health.^6^

The additional costs for the entire SDG agenda in low-income and lower-middle-income countries have been estimated at a minimum of US$1·4 trillion (2013) per year.^7^ However, the health components of these estimates were derived from a pre-2015 analysis of various factors. Global targets and resource-needs estimates for post-2015 investments have been published for specific areas, including HIV/AIDS,^8^ vaccines,^9^ malaria,^10^ tuberculosis,^11^ and health workforce.^12^ However, when considering sector-wide estimates for health systems, WHO's previous estimates, which were produced for the High Level Task Force on Innovative Financing for Health Systems (HLTF) in 2009,^13^ still remain widely quoted.^14^ In these estimates, the mean per-person cost was estimated as an additional $29 by 2015, equivalent to a total of $54 (2005) when added to contemporary health spending ($25). The HLTF estimates reflected primarily an MDG agenda in low-income countries. Other researchers have since inflated the estimates, to $86 in 2012 terms.^15^ The Lancet Commission on Investing in Health drew upon the 2009 HLTF estimates, and estimated that the cost of convergence for low-income countries—with a focus on maternal and child health and communicable diseases—would be $30 billion per year by 2035.^16^, ^17^

We revisit these estimates and provide a new round of WHO estimations of the resource needs for strengthening transformative health systems to reach UHC in the post-2015 era of SDGs.

## Methods

### Definition of the scope

**Figure 1:**
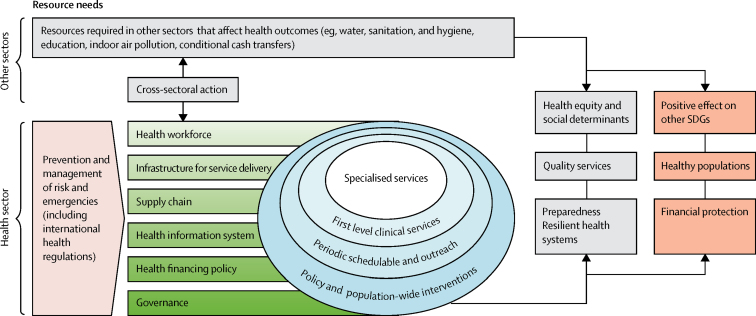
Conceptual framework for transforming health systems towards SDG 3 targets Overall contextual factors include climate change, poverty, migration, and changes in the level and distribution of wealth. Country-specific contextual factors include epidemiological and demographic transitions, urbanisation, and recovery from conflict and disasters. SDGs=Sustainable Development Goals.

Our analysis considers specific SDG targets as integrated parts of the broader attainment of UHC. In addition to SDG 3, we considered other targets for which health is a primary intent and for which we can model costs or outcomes, including SDGs 2, 6, and 7. The investments modelled in our analysis also link to other SDGs, such as those related to education and gender equality (Table 1, Table 2). Attainment of these targets will require the expanded provision of service packages delivered through multiple platforms (figure 1). Our framework places resilient health systems at the centre, with a people-centred approach to service delivery.

**Table 1:** SDG targets and indicators addressed in analysis

	**Indicators for which analysis produces outputs**	**Examples of investments considered in analysis**
**Overall (healthy, longer lives)**		
Life expectancy at birth (years)	Yes	Increased coverage of health services
Healthy life years at birth (years)	Yes	Increased coverage of health services
**SDG 3.1 (reduce maternal mortality)**		
3.1.1 Maternal mortality ratio (per 100 000 livebirths)	Yes	Antenatal care
3.1.2 Proportion of births attended by skilled health personnel	Yes	Skilled attendance at birth
**SDG 3.2 (end preventable neonatal and child deaths)**		
3.2.1 Under-5 mortality rate (per 1000 livebirths)	Yes	Immunisation
3.2.2 Neonatal mortality rate (per 1000 livebirths)	Yes	Essential newborn care
SDG 3.3 (communicable diseases*)		
3.3.1 New HIV infections (per 1000 uninfected population)	Yes	Access to condoms, male circumcision
3.3.2 Tuberculosis incidence (per 1000 population)	No	Expanding tuberculosis treatment
3.3.3. Malaria incidence (per 1000 population at risk)	No	Vector control, antimalarial drugs
3.3.4 Hepatitis B incidence (per 100 000 population)	No	Hepatitis B vaccine
3.3.5 Number of people requiring interventions against neglected tropical diseases	Yes	Drugs for neglected tropical diseases
**SDG 3.4 (reduce mortality from NCDs and promote mental health)**		
3.4.1 Probability of dying from cardiovascular disease, cancer, diabetes, or chronic respiratory disease aged 30–70 years	Yes	Mass media campaigns aimed at reducing risk factors for NCDs
3.4.2 Suicide mortality rate (per 100 000 population)	No	Psychosocial treatment and antidepressants
**SDG 3.5 (strengthen prevention and treatment of substance misuse)**		
3.5.1 Coverage of treatment interventions for substance use disorders	Yes	Screening and brief intervention for hazardous and harmful alcohol use
3.5.2 Total alcohol consumption per person (>15 years), in litres of pure alcohol, projected estimates	No	Increase excise taxes on alcohol
**SDG 3.6 (halve global deaths and injuries from road traffic accidents)**		
3.6.1 Road traffic mortality rate (per 100 000 population)	No	..
**SDG 3.7 (ensure universal access to sexual and reproductive health-care services)**		
3.7.1 Proportion of women of reproductive age (15-49 years) whose needs for family planning are satisfied with modern methods	Yes	Increased uptake of contraceptives
3.7.2 Adolescent birth rate (per 1000 adolescent girls aged 10–14 or 15–19 years)	No†	Adolescent-friendly health services
**SDG 3.8 (achieve universal health coverage, including financial risk protection, access to good-quality essential health-care services, medicines, and vaccines for all)**		
3.8.1 Coverage of essential health services (based on tracer interventions including reproductive, maternal, newborn, and child health, infectious diseases, NCDs, and service capacity and access)	Yes	Increased coverage of services through four platforms
3.8.2 Proportion of population with large household expenditures on health as a share of total household expenditure	No‡	Administrative costs for health financing reform
**SDG 3.9 (reduce deaths and illnesses from hazardous chemicals and air, water, and soil pollution and contamination)**†		
3.9.1 Mortality rate attributed to household and ambient air pollution	No	Expand use of clean cooking stoves and clean fuel
3.9.2 Mortality rate attributed to exposure to unsafe water, unsafe sanitation, and lack of hygiene services	No	Expanding water, sanitation, and hygiene coverage
3.9.3 Mortality rate from unintentional poisoning	No	Poison centres
**SDG 3.a (strengthen implementation of framework convention on tobacco control)**		
3.a.1 Age-standardised prevalence of current tobacco use in people aged 15 years or older	Yes	Plain packaging, enforce bans on tobacco advertising, promotion, and sponsorship
**SDG 3.b (support the research and development of vaccines and drugs, and provide access to drugs for all)**		
3.b.1 Proportion of target population covered by vaccines	Yes	Strengthening the cold chain
3.b.2 Official development assistance to medical research and basic health sectors	No‡	..
3.b.3 Proportion of health facilities that have core set of relevant essential medicines available	No	Drugs provided for essential interventions
**SDG 3.c (increase health financing and health workforce in developing countries)**		
3.c.1 Health worker density and distribution	Yes	Increased production and recruitment
**SDG 3.d (strengthen capacity for early warning, risk reduction, and management of health risks)**		
3.d.1 Average of 13 international health regulations and preparedness core capacity scores	No§	Construction of laboratories, emergency operation centres
**SDG 2.1 (end all forms of malnutrition)**		
2.2.1 Prevalence of stunting in children younger than 5 years	Yes¶	Counselling on complementary feeding practices
2.2.2 Prevalence of malnutrition in children younger than 5 years (wasting and overweight)	Yes¶	Management of severe, acute malnutrition
**SDG 6.1 (achieve universal and equitable access to safe and affordable drinking water)**		
6.1.1 Proportion of population using safely managed drinking-water sources	Yes‖	Provide piped water (eg, borehole, tube well)
**SDG 6.2 (achieve access to adequate and equitable sanitation and hygiene)**		
6.2.1 Proportion of population using safely managed sanitation services, including hand washing	Yes‖	Information campaigns on hand washing
**SDG 7.1 (ensure universal access to affordable, reliable, and modern energy services)**		
7.1.2 Proportion of population with primary reliance on clean fuels and technology	Yes‖	Expand use of clean cooking stoves and clean fuel

All goals were fully or partly included in our analysis except for goal 3.6. Outputs were not modelled for several outcome indicators because of a lack of data (3.7.2) or a lack of projection model (3.4.2). Some of the targets are addressed within the analysis (eg, harmful use of alcohol [3.5], for which we estimate costs related to prevention and counselling); however, we do not project and report outcomes for the exact SDG indicator (3.5.2, which relates to the consumption as measured in litres of alcohol per capita). SDG=Sustainable Development Goal. NCDs=non-communicable diseases.

* End the epidemics of HIV, tuberculosis, malaria, and neglected tropical diseases, and combat hepatitis, waterborne, and other communicable diseases.

† Adolescent maternal mortality is incorporated in aggregate maternal mortality projections.

‡ Our optimistic scenario for expenditure projections is based on normative increases in public expenditure that would be favourable for increasing financial protection and reducing reliance on out-of-pocket payments. However, in our projections we do not specifically look at household health expenditure, nor do we specifically model the share of official development assistance allocated to health.

§ Estimates take into account international health regulations indicators as the basis for assessments of what investments are required, but the model does not project the extent to which capacity would increase.

¶ Analysis only includes underweight (wasting and stunting).

‖ Costs mainly fall in sectors outside the health sector.

**Table 2:** Interlinkages with other SDGs considered within analysis

	**SDG**	**Pathway**	**Direction of effect**	**Examples of investments considered in analysis**
1	Eliminate poverty	Address socioeconomic determinants through cash transfers	Alleviation of poverty leads to health improvements	Cash transfers to poor populations*
4	Quality education	Increase access to contraception to allow women and girls to stay in school, and increase investment in education	Improved access to health services leads to education improvements	Modern contraceptives
5	Gender equality	Cash transfers to address socioeconomic determinants, increase access to contraceptives, expand health workforce labour market opportunities	Investments in poverty reduction and greater access to health services improves gender equality	Cash transfers to poor populations*; recruitment of health workers in rural area
7	Energy	Equip health facilities with renewable sources of energy	Investment in renewable sources of energy within the health system leads to improved energy use	Solar panels for cold chain
8	Decent work and economic growth	Expand health workforce by recruiting an additional 23·6 million health workers; additional jobs would be created in construction, commodity production, and trade	Investment in the health system fosters conditions for decent work and economic growth	Health worker salaries
16	Peaceful inclusive societies	Strengthen equitable health systems to make societies more resilient and stable	Investment in the health system is a precondition for inclusive societies	Construction of new facilities in rural areas

SDG=Sustainable Development Goal.

* Costs mainly fall in sectors outside the health sector.

Our cost estimates included investments to reach minimum required levels in terms of inputs (ie, workforce, health facility density, and laboratories) across the health system. The modelling for the three most resource-intensive health system components (health workforce, infrastructure, and supply chain) was interlinked and closely related to the scope of services provided. Other health system investments (eg, health information systems, emergency risk management, governance and health financing) are more independent of the service package and relate to strengthening institutions. We considered resources needed for strengthening health system performance (eg, governance-related functions such as audits, licensing, and inspection of health providers, contracting out health services), and costs for provision of 187 specific interventions, such as iron and folic acid for pregnant people, and outreach services to high-risk populations for HIV/AIDS (Table 1, Table 2; appendix).

In recognition of links to other sectors, we estimated costs (and, when possible, the associated effect) of increasing access to water, sanitation, hygiene, clean fuels for cooking, and cash-transfer programmes that benefit poor households—interventions that have direct effects on health but the costs of which would not be borne mainly by the health sector. For these cross-sectoral investments, we estimated the share of costs that would be attributed to, and financed by, the health sector as opposed to other sectors. In this Article, we focus on health sector costs, with costs for other sectors described in detail in the accompanying technical report.^18^

Although the SDGs concern all countries, we limited our analysis to low-income and middle-income countries, because these countries are faced with the greatest challenges in terms of increasing service provision and resource mobilisation (appendix). Our model included all low-income countries, the 20 most populous lower-middle-income countries, and the 20 most populous upper-middle-income countries (thereby including large countries such as China, India, and Indonesia). We excluded four countries for which gross domestic product (GDP) data were lacking, so our final sample was 67 countries. These countries represent 95% of the total population in low-income and middle-income countries, and include a set of the most vulnerable conflict-affected and fragile nations (appendix).

### Pathways to UHC

Progressive universalism^17^ and the building of sustainable, resilient health systems capable of ensuring equitable access though a people-centred service delivery approach are at the centre of our model. We considered four service delivery platforms, representing different modes for providing patients with information, counselling, essential preventive commodities, screening, diagnosis, treatment, and follow-up—a continuum of care (appendix).

In view of the global nature of our analysis, we set targets consistent with SDG 2030 global targets on the basis of global best practices, including globally accepted health system benchmarks and WHO intervention guidelines and recommended practices.^8^, ^9^, ^10^, ^11^, ^12^, ^19^ We modelled a progressive expansion of service coverage as health systems developed. We recognised that some types of services face fewer implementation challenges and therefore can be scaled up faster than other more complex services. For example, services delivered through the policy and population-wide or periodic schedulable and outreach platforms (eg, bednets) require less well developed infrastructure and referral chains than do specialised care services (eg, cancer treatment). When setting targets, we took into account the probable attainable frontiers for different types of service delivery platforms (figure 1). For example, management of non-communicable diseases is modelled to reach a maximum of 60% coverage, a level that many high-income countries have not reached. Other services, such as maternal, child, and immunisation services, were projected to potentially reach 95% coverage.

Acknowledging the diversity in low-income and middle-income countries, we grouped countries into five types—conflict-affected countries, countries with vulnerable systems, and countries in health systems categories 1, 2, and 3 (appendix)—to determine the timing and duration of strategic investments. Conflict-affected countries are those with an internal or external conflict which considerably limits the state's ability to provide health services. Vulnerable countries were those with structural vulnerabilities—such as localised conflicts, a weak state apparatus, external international humanitarian response structures, or health crises (eg, Ebola)—that score high on fragility. Health systems categories refer to health system strength, with proxies for scale-up capacity based on existing resources and current service delivery performance (appendix). Each country was expected to make progress towards UHC, but is by necessity constrained by the level of development of the existing health system, especially human resources and functional infrastructure. Conflict-affected and emergency-affected states in particular require stability before capital investments can be made to strengthen the foundations of health systems. More stable systems (eg, countries in health systems categories 2 and 3) could scale up more rapidly within our model.

Because of uncertainty about the capacity of health systems to absorb additional resources in a timely manner,^20^ we modelled two scenarios with differing levels of ambition: a progress scenario, in which countries' advancement towards global targets was constrained by their health systems' assumed absorptive capacity, and an ambitious scenario, in which most countries attained global targets (appendix). Scale-up trajectories in the two scenarios were driven by characteristics of different interventions and delivery platforms. We modelled that policy and population-wide interventions and periodic schedulable and outreach services would be rapidly scaled up for all countries in both scenarios, whereas facility-based services would follow the pathways of health-system strengthening, where the two scenarios increasingly diverge (appendix). Throughout the modelling, we incorporated costs for reducing inequities, including reorientation of health systems to practices that favour inclusiveness and explicit adjustments for special populations (appendix).

### Projection of costs, effects, and financing

In this analysis, we use more robust and comprehensive methods and tools than were used in the previous 2009 HLTF estimates. For direct intervention-related costs and effects, we used Spectrum-based OneHealth tool, which takes an integrated approach to the assessment of costs and health benefits, and incorporates interlinked epidemiological reference models. Targets were aligned with published disease-specific costs in terms of priority health interventions and 2030 targets,^8^, ^9^, ^10^, ^11^, ^12^, ^19^, ^21^ and combined within a system-wide perspective. We computed the estimated need for health services dynamically over time, taking into account population growth, reduced mortality, and reduced incidence or prevalence of disorders as coverage of interventions (preventive and curative) increased. Analysis with the OneHealth tool was complemented by Excel-based models, when needed, and system-specific components were excluded from disease-specific costs to avoid double counting.^18^ We used a bottom-up, inputs-based costing approach (quantities times price), taking into account a steady closing of the gap between current and target investments year by year. Inputs were multiplied by country-specific prices from the WHO-CHOICE database and other publicly available sources. We report costs in non-inflation adjusted 2014 US$.

Projected health outcomes are reported in line with the SDG indicator framework and include improved nutrition, reduced disease prevalence and age-specific mortality rates. On the basis of OneHealth tool projections and Spectrum outputs, we estimated the rise in life expectancy as a result of increased intervention coverage, and compared this increase to 2015 life expectancy. Tuberculosis^11^, ^22^ and neglected tropical disease^23^ outcomes were adapted from earlier studies. We ran life-expectancy projections for 18 countries representing 60% of the global burden of disease (2010) and 79% of the population of the 67-country set. We also modelled a second summary effect measure: the projected increase in healthy years lived across all 67 countries.

On the basis of International Monetary Fund data (from October, 2016), we developed two main financial space scenarios by country, incorporating GDP projections and assumptions on available government revenues and government health priorities (appendix). Projections detail the financial space for total health expenditure to assess the potential envelope of available resources, and focus on fiscal space and general government health expenditure, which have central roles in advancing UHC through prepayment, cross-subsidies, pooling, and strategic purchasing.^3^ To assess affordability and the financing gap, we calculated the incremental cost by year, and compared this cost with the projected available financing by country and year.

Because investments in infrastructure peak in 2029 (such that access to services is maximised in 2030), we report additional costs in billions as the mean annual need during a mature (ie, end-term) scale-up phase (2026–30). Additional costs per-person are reported for 2030. To provide an estimate similar to the previously published estimate of $86,^15^ we also calculated a measure for total cost per person, which we defined as total current health expenditure (reported in 2014 in national health accounts) plus the estimated incremental cost by country-year from our model.

### Consultation and review

A consultation and review process shaped this analysis. We took into account the breadth of previous work and suggestions on what should be included in the scope of the exercise. A WHO and UNAIDS expert group met monthly to provide inputs on the framework and modelling approach from the perspective of individual disease areas and health system building blocks. In July 2016, WHO organised an expert review and country feedback meeting to discuss the methodology and preliminary results of the analysis. Participants included international experts and academics, and representatives from 14 low-income and middle-income countries, who jointly accounted for more than 75% of the population covered in the analysis. Country participants reviewed country-specific input assumptions, and their feedback was incorporated into the models.

### Role of the funding source

The study funders had roles in study design; data collection, analysis, and interpretation; and writing of the Article. The corresponding author had full access to all the data in the study and had final responsibility for the decision to submit for publication.

## Results

The progress scenario costs increased over time, from an initial $104 billion annually to $274 billion per year in 2026–30, the final years of scale-up, or $41 per person (range $15–102) by 2030. The ambitious scenario would require annual additional investments of $134 billion per year initially, reaching $371 billion in 2026–30; the equivalent mean per-person estimate for 2030 was $58, which varied widely by country (range 22–167).

Adding incremental costs of the ambitious scenario to current spending would produce an estimated mean total cost per person in 2030 of $271 for all 67 countries (table 3). In the ambitious scenario, additional costs represent a mean of 4·6% of projected GDP in 2030 (range 0·2–17·9), and adding these costs to current health spending is projected to increase health spending as a share of GDP from a mean of 5·6% (2·2–10·8) to a mean of 7·5% (2·1–20·5) for the entire sample (appendix). In the model, conflict-affected countries, countries with vulnerable systems, and countries in health system category 1 had the greatest increases in health spending as a proportion of GDP over time (appendix), because these countries have the largest current gaps and slowest forecasted GDP growth.

**Table 3:** Estimated additional resource needs, by country typology and income group

	**n**	**Mean population by year during end-term scale up 2026–30 (millions)**	**Total additional cost 2016–30 (billions)**	**Mean annual cost (billions)**			**% of costs classified as health-system costs**	**Additional incremental investment need per person (2030)**			Modelled total cost per person, 2030 (THE)*			**Modelled total cost per person, 2030 (GGHE)**†		
				Initial scale-up (2016–20)	Mid-term scale-up (2021–25)	End-term scale-up (2026–30)		Population-weighted mean	Minimum	Maximum	Population-weighted mean	Minimum	Maximum	Population-weighted mean	Minimum	Maximum
**Progress scenario**																
All countries	67	6402	2920	104	205	274	74%	41	15	102	249	60	979	149	40	496
Conflict-affected countries	4	111	90	3	6	9	77%	77	54	97	187	70	373	136	62	257
Vulnerable systems	11	250	202	3	13	24	79%	87	35	102	133	81	231	100	48	129
Health system category 1 countries	15	455	304	12	22	27	78%	55	33	98	79	60	154	66	50	116
Health system category 2 countries	16	3044	1303	46	91	123	75%	38	31	84	101	63	339	59	40	206
Health system category 3 countries	21	2542	1021	40	73	91	70%	36	15	83	474	159	979	278	101	496
Low-income countries	28	708	524	16	37	52	76%	66	35	98	92	60	154	71	48	116
Lower-middle-income countries	21	3402	1471	51	102	141	75%	40	15	102	130	63	339	72	40	206
Upper-middle-income countries	18	2291	923	37	66	82	69%	36	30	84	519	264	979	303	132	496
**Ambitious scenario**																
All countries	67	6286	3944	134	284	371	75%	58	22	167	271	74	984	168	57	511
Conflict-affected countries	4	110	99	3	6	10	78%	94	68	101	206	93	392	154	80	276
Vulnerable systems	11	334	272	4	17	33	82%	93	53	167	127	104	297	103	66	195
Health system category 1 countries	16	471	396	15	30	34	79%	71	46	141	94	74	198	82	64	160
Health system category 2 countries	16	2800	1756	59	126	163	76%	57	37	120	126	79	342	80	57	233
Health system category 3 countries	21	2571	1422	52	102	127	72%	50	22	89	486	164	984	291	106	511
Low-income countries	28	804	671	20	48	66	79%	76	46	141	112	74	198	91	64	160
Lower-middle-income countries	21	3127	1942	65	138	183	77%	58	22	167	146	76	342	89	57	210
Upper-middle-income countries	18	2355	1330	49	96	119	71%	51	36	118	536	297	984	320	134	511

Data are in US$ (2014). Income groups were defined as of July, 2016. Per person costs are reported as population-weighted mean values per group for the year 2030. If the mean annual investment need per person during the full 5 years of the end-term scale-up phase 2026–30 were considered instead, values in the ambitious scenario per person would be $59 overall, $95 in conflict-affected countries, $99 in countries with vulnerable systems, $73 in health system category 1 countries, $59 in health system category 2 countries, $50 in health system category 3 countries, $82 in low-income countries, $59 in lower-middle-income countries, and $51 in upper-middle-income countries—some per-person costs would thus be higher than the 2030 value, particularly in low-income and vulnerable countries. Because of rounding, numbers might not add up. THE=total health expenditure. GGHE=general government health expenditure.

* Computed as current THE in 2014 plus modelled additional cost in 2030, divided by the projected population in 2030.

† Computed as current GGHE in 2014 plus modelled additional cost in 2030, divided by the projected population in 2030.

The annual funding gap in 2026–30 when the two resource-needs scenarios were paired with an optimistic and a more moderate financing scenario, was estimated at $20–54 billion for all 67 countries (table 4). 23–32 countries are projected to face a funding gap, 20–27 of which are low-income countries (table 4). Countries affected by conflict, with vulnerable systems, or in health system category 1 can mobilise only some domestic resources in both the optimistic and moderate financing scenarios (appendix). Countries in health system categories 2 and 3, where most of the sample's population resides, account for a high share (80%) of additional costs (table 3), but were projected to have the greatest ability to move towards UHC through domestic financing (appendix).

**Table 4:** Estimated mean annual financing gap 2026–30, by country group

	**Optimistic financing scenario**			**Moderate financing scenario**		
	n	Population (millions)	Billions US$ (2014)	n	Population (millions)	Billions US$ (2014)
**Progress scale-up**						
All countries	23	624	20	28	958	30
Conflict-affected countries	3	60	3	3	60	3
Vulnerable systems	7	178	11	9	231	13
Health system category 1 countries	12	276	6	14	383	8
Health system category 2 countries	1	110	0·5	2	284	5
Low-income countries	20	436	17	24	598	22
Lower-middle-income countries	3	187	2	4	359	8
Upper-middle-income countries	0	0	0	0	0	0
**Ambitious scale-up**						
All countries	30	1083	41	32	1189	54
Conflict-affected countries	3	59	4	3	79	4
Vulnerable systems	10	323	19	11	333	22
Health system category 1 countries	14	402	11	14	437	15
Health system category 2 countries	3	299	8	4	340	14
Low-income countries	26	737	29	27	777	35
Lower-middle-income countries	4	346	12	5	432	19
Upper-middle-income countries	0	0	0	1	27	0·2

This table includes only countries for which projected costs exceed the projected available financing in one or more years during the end-term scale-up period—ie, there is a financing gap during at least one of the years 2026–30 within the modelled projections. Population and cost data refer to the year or years in which a financing gap has been projected. If the gap lasts for more than 1 year, the results represent the mean gap and population size during those years. n=the number of countries within each group that is projected to have a financing gap during at least 1 year.

**Figure 2:**
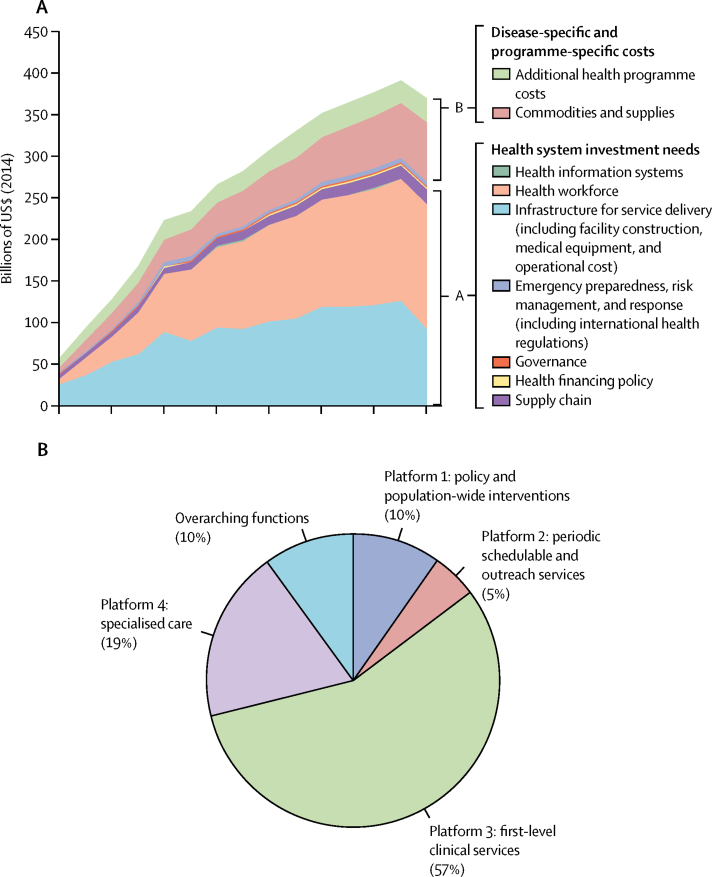
Additional investments required in 67 low-income and middle-income countries to meet Sustainable Development Goal 3 (US$ 2014 billion) (A) and additional resource needs by service delivery platform (B) in the ambitious scenario Additional health programme costs include those that are programme specific but do not refer to specific drugs, supplies, or laboratory tests. Examples include costs for programme-specific administration staff, supervision, and monitoring relative to the services for which the programme provides leadership and oversight (eg, the national malaria programme provides implementation guidance, and monitors and supervises service delivery for malaria). Other examples include mass media campaigns and demand generation. These data are presented as a table in the appendix.

Around 75% of the additional cost is for health systems; health workforce and health facilities (including equipment and operating costs) are the main cost drivers (figure 2A). The ambitious scenario projections add more than 23·6 million health workers, 3·0 million of whom would be medical doctors, and includes the construction of over 415 000 health facilities, 378 000 of which would be primary health centres (appendix). Most resources will be needed to support first-level (ie, primary) clinical services (figure 2B). Such investments would bring health workforce population densities for nurses and midwifes above current densities in upper-middle income countries (table 5). Among programme-specific costs, non-communicable diseases account for 44% of costs (appendix).

**Table 5:** Moving health systems closer to convergence on public health system benchmarks

	**Health worker density**			**Hospital beds (per 1000 population)**	**Total health expenditure per person (US$ 2014)**		**Life expectancy (years)**
	Doctors (per 1000 population)	Nurses or midwives (per 1000 population)	Other health workers (per 1000 population)		Current and projected	Projected minimum health spending need by 2030	
OECD (current, 2014)	2·76	6·61	3·52	4·68	4760	N/A	80·1
Upper-middle-income countries in sample (current, 2014)	1·64	2·56	2·56	3·08	472	N/A	75·9
Low-income countries in sample (projected 2030)	1·18	3·21	3·30	1·46	76*	114	68·6†
Lower-middle-income countries in sample (projected 2030)	1·43	4·07	3·52	2·35	275*	182	72·5‡
Upper-middle-income in sample (projected 2030)	1·78	4·11	3·07	3·13	953*	533	78·6§

Data are average estimates per country group. Data are from WHO, the OECD, or WHO Global Health Observatory and National Health Planning Documents. Projections are for the ambitious scale-up scenario, unless otherwise specified. OECD=Organisation for Economic Co-operation and Development. N/A=not applicable.

* Projections are for optimistic health financing scenario.

† Number of countries=3.

‡ Number of countries=10.

§ Number of countries=5.

**Figure 3:**
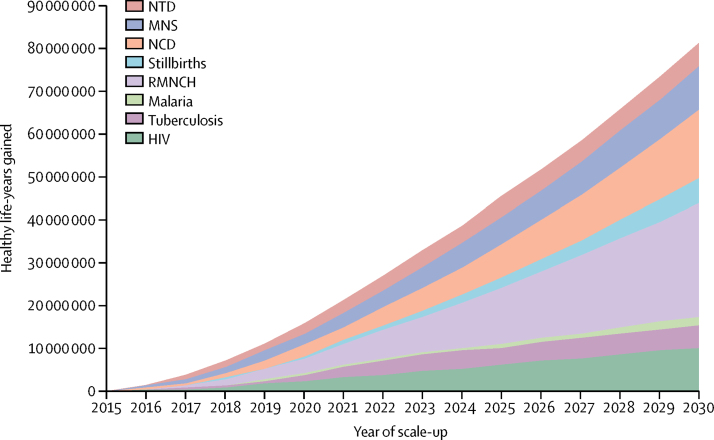
Projected healthy life-year gains, compared with the flatline scenario, as a result of intervention scale-up in the ambitious scenario (67 countries) NTD=neglected tropical diseases. MNS=Mental health and substance use. NCD=non-communicable disease. RMNCH=reproductive, maternal, newborn, and child health.

If additional funds were used as described, 97 million lives could be saved and life expectancy could increase by as much as 8·4 years (Table 6, Table 7; appendix). The 67 countries would see a total gain of 535 million healthy life-years during the SDG period, with 81 million healthy life-years gained in 2030 (figure 3).

**Table 7:** Projected increases in health and wellbeing

	**Sustainable Development Goal target**	**Baseline**	**Progress scale-up**	**Ambitious scale-up**
**Deaths averted (2016–30)**				
Stillbirths	N/A	N/A	6 700 000	11 400 000
Neonatal deaths (0–1 years)	3·2	N/A	13 800 000	19 400 000
Post-neonatal deaths (1–4 years)	3·2	N/A	15 400 000	21 500 000
Maternal deaths	3·1	N/A	1 500 000	2 100 000
Cancer deaths	3·4	N/A	2 900 000	4 300 000
Cardiovascular disease, diabetes, depression, and epilepsy	3·4	N/A	11 650 000	16 130 000
Tuberculosis	3·3	N/A	11 200 000	11 200 000
HIV/AIDS	3·3	N/A	8 100 000	10 800 000
**Additional health outcomes (2016–30)**				
Additional unplanned births averted if unmet need for family planning is satisfied	3·7	N/A	153 000 000	400 000 000
Unsafe abortions averted because modern contraception provided	3·7	N/A	71 900 000	146 200 000
**Additional health outcomes (2030)**				
Total fertility rate	3·7	3·64	3·0	2·4
Stunting* prevalence in children aged 0–5 years	2·2	32·2	28·7	28 100 000
Number of children in whom stunting* would be prevented	2·2	N/A	51 800 000	87 000 000
Wasting† prevalence in children aged 0–5 years	2·2	9·0	8·1	7·7
Number of children in whom wasting† would be prevented	2·2	N/A	22 700 000	36 800 000
Maternal mortality rate (deaths per 100 000 livebirths)	3·1	327	208	174
Proportion of births attended by skilled health personnel	3·1	69·6	85·1	92·7
Under-5 mortality rate (deaths per 1000 livebirths)	3·2	55	35	29
Neonatal mortality rate (deaths per 1000 livebirths)	3·2	22	13	10
Annual number of new HIV infections	3·3	1 676 000	720 000	197 000

Data are the totals for 67 countries. Ambitious and progress scenario scale-up refer to additional health outcomes attained by expanding service coverage beyond current (flatline) coverage, and whereby the ambitious scenario has higher targets than the progress scenario (the appendix includes more detail on target setting).

* More than two SDs less than the median normal height for age.

† More than two SDs less than the median normal weight for height.

**Table 6:** Life expectancy gains 2015–2030, compared with alternative comparators

	**Number**	**Projected life expectancy gain in flatline scenario**	**Ambitious scenario**	
			Additional life expectancy gain directly because of Sustainable Development goal package*	Total life expectancy gain compared with baseline†
Conflict-affected countries	2	1·39	1·74	3·12
Vulnerable systems	2	3·13	5·24	8·37
Health system category 1 countries	2	2·84	3·89	6·73
Health system category 2 countries	6	2·23	3·27	5·50
Health system category 3 countries	6	2·66	1·17	3·83

Results are modelled for 18 countries and include the projected effect of scaling up HIV/AIDS, maternal and child health (including stillbirth prevention), and a set of non-communicable diseases (eg, cardiovascular disease, diabetes, asthma, chronic obstructive pulmonary disease, epilepsy, mental disorders, neurological disorders, and substance use disorders). Results are shown as population-weighted estimates per country category.

* Estimated increase in life expectancy as a result of the interventions considered within the analysis, based on comparisons between 2015 life expectancy and the scenario with ambitious coverage increase.

† Modelled difference in life expectancy between projecting the 2015 coverage level through to 2030 with existing population profile and life expectancy in the modelled ambitious scale-up scenario. This estimate provides a more conservative increase in life expectancy attributed to the modelled interventions directly, and excludes projected health improvements as captured within the UN population projections. The reporting of life expectancy is valid given that, within our model, we project an expansion of health systems that will serve conditions beyond those explicitly identified within our intervention list. With the exception of the countries with the strongest health systems at baseline, the interventions being scaled up would, in most cases, more than double the projected life expectancy gains.

## Discussion

According to our model, an additional $371 billion will be needed per year for low-income and middle-income countries to reach the health-related SDG targets. Our estimate is higher than those from previous modelling studies. The UN Sustainable Development Solutions Network estimated a yearly additional resource need for all the SDGs of $1·4 trillion, with required resources for health estimated to be $69–89 billion.^7^ Our estimates are also higher than the commonly cited benchmark of $86 per person, derived from the HLTF analysis for the MDGs.^15^

Our ambitious scenario estimates of projected country total costs ranged from $74–984 (mean $271) per person per year. However, our estimates differ from previous ones in terms of the number and type of countries included (our analysis included more middle-income countries than did previous analyses), which makes direct comparison complicated. A more relevant comparison with the HLTF estimates would be to consider low-income countries only, for which we estimated an additional $76 per person for ambitious targets, or a projected total cost of $112 (table 3). Differences between this set and previous estimates are driven by new and more ambitious health system benchmarks (eg, health workforce density), the scope of the costing (with our inclusion of emergency risk management, non-communicable diseases, etc), the level of ambition for disease-specific targets (eg, for HIV/AIDS),^8^ and higher current (ie, baseline) health spending. Our presentation of mean, minimum, and maximum estimates by country group underline the varied investment needs and should be understood as a caveat against adopting a single number.

About three-quarters of additional investments need to go towards health-systems strengthening. This finding is consistent with those of the HLTF (2009) and confirms the findings of the four main Commissions (by WHO, Harvard University and the London School of Hygiene & Tropical Medicine, the US National Academy of Medicine, and the UN) in the wake of Ebola, that health systems were underfunded in the MDG era.^24^, ^25^ Substantial investments are needed to put infrastructure, health workforce, and equipment in place and to provide essential health services—all of which are required to attain the SDG targets. A key public health concern today is the shortfall of health workers in a context of global shortage of health skills.^26^ Health workers and infrastructure are a public necessity, not luxuries: even if countries implement our proposed model, they would still fall short of current system capacity in countries in the Organisation of Economic Co-operation and Development (table 5).

Middle-income countries are well equipped to self-finance the investment—the financing gap is mostly in low-income countries. Some middle-income countries might even set more ambitious targets than we did in this analysis, targets that address broader health issues, including ageing and further boosting the quality of care, which require more resources. Of the total annual financing gap of $20–54 billion per year, $17–35 billion per year falls on low-income countries, with conflict-affected countries burdened with a gap of $3–4 billion (table 4). Many countries will thus continue to need external financial support throughout the period of the SDGs, mostly to build the foundations of their health systems.^3^, ^7^

However, even the poorest countries can reach some level of universality. In settings where clinical services are still underdeveloped and human resources for health are critically low, there is potential to rapidly move towards full coverage with interventions that can be delivered through non-clinical service delivery platforms. All countries could afford universal access to the range of public health services delivered through mostly policy, population-wide, and periodic schedulable and outreach delivery platforms (appendix). Examples include effective policy interventions to curb the rise in non-communicable diseases, which could substantially reduce future expenses on disease management^27^—eg, fiscal policies, such as public health taxes on goods harmful to health, including tobacco, alcohol, and sugar.^28^

Investments on the scale modelled would bring countries closer to UHC standards and could save 97 million lives. The modelled increase in life expectancy and gains in healthy life-years—overall measures of UHC impact that should be considered in addition to disease-specific SDG indicator reporting—is substantial. Estimates of healthy life-years gained are crucial for diseases for which treatment focuses on quality of life rather than cure. For example, mental, neurological and substance use disorders contribute only 3% of projected life expectancy gain, but 15% of the projected healthy life-years gained. We also expect a reduction in out-of-pocket payments with time as universal, obligatory pre-paid financing for UHC expands.^29^

Improvement of the efficiency of current systems will be crucial to reach SDG targets. In our modelled scale-up, we assume efficient practices. However, evidence shows that resources are not always used to their best potential.^29^, ^30^, ^31^ Although expectations of zero wastage might be unrealistic, we considered scenarios that would improve system efficiencies (eg, shifting to generic drugs, reducing fraud and corruption), thereby effectively freeing up resources and decreasing overall projected costs. A converse argument would be that weak capacity in low-income countries increases the costs of making improvements, and that current inefficiencies could be assumed to also be prevalent in future systems, implying that costs should be higher than those presented here. In the appendix we present cost estimates in high-efficiency and low-efficiency scenarios.

Investment in health should accelerate progress in other SDG areas, and vice versa. Our results are consistent with previous findings that investment in the health sector alone is not sufficient to attain global health targets (table 6). For example, under 5 mortality is modelled to reach 29 per 1000 livebirths on average, but the global target is 25. Important additional factors include multisector engagement, emphasis on accountability, and alignment of stakeholder action.^32^ Strengthening of the health workforce should result in direct and indirect contributions to economic growth, as noted by the High Level Commission on Health Employment and Economic Growth.^24^ Several studies^17^, ^33^ have shown substantial economic gains as a result of investments in health.

Although global estimates are useful advocacy tools to highlight investment needs, our cost estimates are not to be interpreted as universal spending targets that would apply for every country. Contexts are diverse, and spending a specific amount per person will not necessarily produce a specific outcome, nor would spending a specific amount per person result in the same outcome in two different countries. To advocate for SDG investments, progress needs to be monitored and global estimates need to be regularly updated accordingly, taking into account new evidence, improved projection models, and the emergence of new technologies.

We acknowledge the limitations of our work, many of which concern uncertainties related to projections. GDP forecasts are uncertain and are a major determinant of our financing projections and subsequent analyses of affordability. Uncertainties increase as the projections stretch into the future. Another limitation is the exclusion of some SDG conditions or targets, such as road traffic accidents, hepatitis treatment, and chemical poisoning, because costing frameworks and impact models were not available. We were unable to include interventions for which current levels of coverage or benchmarks could not be identified (eg, oral health, assistive technologies). Our estimates should therefore be considered as minimum indicative estimates.

Our estimates should be considered a starting point for discussions. Not every country can provide the full range of services recommended to attain the SDGs at the same speed, but every country can make substantial progress during the next 15 years. With 17 development goals, health and development advocates in each country will need to make the case as to why health care should be prioritised financially. Strong public financial management and good implementation capacity will be required to use resources effectively in making progress towards the SDGs. The investment case for health is strong and can be easily made. Each country should use available evidence and tools to prioritise equitably, plan strategically, and cost realistically its own path towards SDG 3 and UHC.
