# Supplementary material for: Financing transformative health systems towards achievement of the health Sustainable Development Goals: a model for projected resource needs in 67 low-income and middle-income countries
Source: Lancet Glob Health. 2017 Jul 17;5(9):e875–87. doi: 10.1016/S2214-109X(17)30263-2 (PMC5554796; doi:10.1016/S2214-109X(17)30263-2)
Supplement: Supplementary appendix [file mmc1.pdf]

THE LANCET
Global Health

Supplementary appendix

This appendix formed part of the original submission and has been peer reviewed. We post it as supplied by the authors.

Supplement to: Stenberg K, Hanssen O, Tan-Torres Edejer T, et al. Financing transformative health systems towards achievement of the health Sustainable Development Goals: a model for projected resource needs in 67 low-income and middle-income countries. *Lancet Glob Health* 2017; published online July 17. http://dx.doi.org/10.1016/S2214-109X(17)30263-2.

Financing transformative health systems towards achievement of the health Sustainable Development Goals: a model for projected resource needs in 67 low-income and middle-income countries

Karin Stenberg, Odd Hanssen, Tessa Tan-Torres Edejer, Melanie Bertram, Callum Brindley, Andreia Meshreky, James E Rosen, John Stover, Paul Verboom, Rachel Sanders, Agnès Soucat

Supplementary Material

This document provides supplementary information to the main paper.[1] It has eight sections:

Section 1: List of countries included in the analysis
Section 2: Country groups and pathways towards Universal Health Coverage
Section 3: Start and end points within models
Section 4: Cost and impact projection methods
Section 5: Methods for projecting available financing
Section 6: Review processes
Section 7: Efficiency considerations
Section 8: Additional results, tables and figures

Section 1. List of countries included in the analysis

While the SDGs concern all countries, our model includes only low and middle income countries, as these are faced with the greatest challenges in terms of health burden and mobilisation and effective use of resources. We model estimates for all low-income countries, the 20 most populous lower middle income countries and the 20 most populous upper middle income countries.[2] When excluding 4 countries lacking GDP data we are left with a total of 67 countries, in size representing 95% of the total population in low and middle income countries, including a set of the most vulnerable conflict-affected and fragile contexts (Table S1).

[1] For readers wishing additional detail to what is outlined within this document, please contact the corresponding author (stenbergk@who.int; or whochoice@who.int)

[2] The selection of countries was performed in March 2016. At this time, the Russian Federation was classified as a high income country, which is why it is not included among the 67 countries. It was reclassified to an upper middle income country on July 1st, 2016. Furthermore, Cambodia and Tunisia were both reclassified as lower middle income on July 2016, which means that the resulting country list for which results are presented here includes 28 LICs, 21 LMICs and 18 UMICs.

Table S1. List of countries included in the analysis

Country	Income Group*	WHO epidemiological region	Population (2015)**	Resource Availability ***	Skilled Birth Attendance ****	Human Resources for Health *****	Population affected by conflict (%) ******	Fragility Index Score *******
Afghanistan	LIC	EMRD	32,526,562	2,000	45.2	0.72	8.3	47
Algeria	UMIC	AFRD	39,666,519	13,880	96.6	5.03	-	31
Angola	UMIC	AFRD	25,021,974	7,227	46.7	1.57	-	35
Azerbaijan	UMIC	EURB	9,753,968	16,920	97.2	9.60	-	28
Bangladesh	LMIC	SEARD	160,995,642	3,330	41.7	0.56	-	36
Benin	LIC	AFRD	10,879,829	2,020	77.2	0.79	-	37
Brazil	UMIC	AMRB	207,847,528	15,570	99.1	9.15	-	26
Burkina Faso	LIC	AFRD	18,105,570	1,600	65.9	0.57	-	40
Burundi	LIC	AFRE	11,178,921	770	60.3	0.48	-	42
Cambodia	LMIC	WPRB	15,577,899	3,080	89.0	0.93	-	35
Cameroon	LMIC	AFRD	23,344,179	2,950	64.7	0.49	1.5	38
Central African Republic	LIC	AFRE	4,900,274	600	40.0	0.29	46.9	46
Chad	LIC	AFRD	14,037,472	2,070	24.3	0.48	-	44
China	UMIC	WPRB	1,376,048,943	13,170	99.9	3.10	-	26
Colombia	UMIC	AMRB	48,228,704	12,910	98.7	2.09	-	31
Comoros	LIC	AFRD	788,474	1,430	82.2	0.49	-	39
Côte d'Ivoire	LMIC	AFRE	22,701,556	3,130	56.4	0.56	-	42
Democratic Republic of the Congo	LIC	AFRE	77,266,814	650	80.1	0.08	2.1	46
Dominican Republic	UMIC	AMRB	10,528,391	12,600	97.7	2.79	-	30
Ecuador	UMIC	AMRD	16,144,363	11,190	96.3	3.78	-	30
Egypt	LMIC	EMRD	91,508,084	10,280	91.5	5.69	-	36
Eritrea	LIC	AFRE	5,227,791	1,529	34.1	0.64	-	42
Ethiopia	LIC	AFRE	99,390,750	1,500	15.5	0.26	-	41
Gambia	LIC	AFRD	1,990,924	1,580	57.2	0.90	-	37
Ghana	LMIC	AFRD	27,409,893	3,900	70.8	0.97	-	32
Guinea	LIC	AFRD	12,608,590	1,130	45.3	0.49	-	45
Guinea-Bissau	LIC	AFRD	1,844,325	1,380	45.0	0.61	-	44
Haiti	LIC	AMRD	10,711,067	1,730	37.3	0.51	-	45
India	LMIC	SEARD	1,311,050,527	5,630	74.4	2.30	-	34
Indonesia	LMIC	SEARB	257,563,815	10,190	87.4	1.54	-	30
Iran (Islamic Republic of)	UMIC	EMRB	79,109,272	15,688	96.4	4.98	-	32
Iraq	UMIC	EMRD	36,423,395	15,100	90.9	3.86	27.5	42
Kazakhstan	UMIC	EURC	17,625,226	21,710	99.5	11.48	-	26
Kenya	LMIC	AFRE	46,050,302	2,940	61.8	1.02	-	41

Liberia	LIC	AFRD	4,503,438	700	61.1	0.27	-	45
Madagascar	LIC	AFRD	24,235,390	1,400	44.3	0.50	-	40
Malawi	LIC	AFRE	17,215,232	790	87.4	0.34	-	38
Malaysia	UMIC	WPRB	30,331,007	24,770	99.0	4.38	-	25
Mali	LIC	AFRD	17,599,694	1,510	57.1	0.45	2.7	44
Mexico	UMIC	AMRB	127,017,224	16,840	98.7	4.45	-	31
Morocco	LMIC	EMRD	34,377,511	7,290	73.6	1.45	-	28
Mozambique	LIC	AFRE	27,977,863	1,120	54.3	0.42	-	40
Myanmar	LMIC	SEARD	53,897,154	4,546	77.9	1.60	0.7	37
Nepal	LIC	SEARD	28,513,700	2,410	48.2	0.51	-	36
Niger	LIC	AFRD	19,899,120	910	29.3	0.14	-	44
Nigeria	LMIC	AFRD	182,201,962	5,710	35.2	1.92	0.8	41
Pakistan	LMIC	EMRD	188,924,874	5,090	52.1	1.35	-	44
Peru	UMIC	AMRD	31,376,670	11,440	89.9	2.56	-	27
Philippines	LMIC	WPRB	100,699,395	8,450	72.8	1.43	-	37
Romania	UMIC	EURB	19,511,324	19,950	98.7	9.03	-	21
Rwanda	LIC	AFRE	11,609,666	1,630	90.7	0.76	-	36
Sierra Leone	LIC	AFRD	6,453,184	1,770	59.7	0.18	-	40
South Africa	UMIC	AFRE	54,490,406	12,700	94.3	5.75	-	28
South Sudan	LIC	AFRD	12,339,812	1,800	17.2	1.34	18.6	49
Sri Lanka	LMIC	SEARB	20,715,010	10,300	98.6	2.30	-	32
Sudan	LMIC	EMRD	40,234,882	3,920	19.9	1.06	5.5	45
Tanzania, United Republic of	LIC	AFRE	53,470,420	2,510	48.9	0.43	-	37
Thailand	UMIC	SEARD	67,959,359	14,870	99.6	2.44	-	29
Togo	LIC	AFRD	7,304,578	1,290	44.6	0.31	-	37
Tunisia	LMIC	EMRD	11,253,554	11,020	73.6	4.36	-	31
Turkey	UMIC	EURB	78,665,830	19,560	97.4	3.92	-	28
Uganda	LIC	AFRE	39,032,383	1,720	58.0	0.51	-	40
Ukraine	LMIC	EURC	44,823,765	8,560	99.0	11.38	5.1	32
Uzbekistan	LMIC	EURB	29,893,488	5,830	99.6	14.64	-	31
Viet Nam	LMIC	WPRB	93,447,601	5,350	93.8	2.33	-	27
Yemen	LMIC	EMRD	26,832,215	3,586	43.0	0.80	10.1	46
Zimbabwe	LIC	AFRE	15,602,751	1,650	80.0	1.29	-	41

* Classification as of July 2016, World Bank Atlas method. At the time of country selection (March 1st, 2016), two countries had another classification: Cambodia-low income; and Tunisia-upper middle income.

** United Nations, Department of Economic and Social Affairs, Population Division, World Population Prospects: The 2015 Revision, New York, 2015

*** Gross National Income per capita, adjusted for purchasing power parity, from the World Bank World Development Indicators, accessed on June 3rd, 2016. For countries where data on GNI per capita was unavailable, we used Gross Domestic Product per capita, adjusted for purchasing power parity (Angola, Eritrea, Iran, Myanmar, and Yemen).

**** From the WHO global health observatory, at the time of country selection, March 1st, 2016.

*****Defined as the combined measure of doctors, nurses and midwives per 1000 population. Data from the WHO global health observatory, May 24th, 2016.

****** Percentage of the population in a country affected by a conflict, taken from WHO Humanitarian Response Plans and WHO country Health Resource Availability Mapping Systems.

******* Composite score of five subcomponents of the Fund for Peace Fragility Index, which were demographic pressures, poverty and economic decline, limits to the provision of public services, inexistence of a security apparatus, and presence of external intervention. In addition to these final two indicators, we also considered countries that had undergone a serious shock to their health systems in the past five years (Guinea, Liberia, Mali, and Sierra Leone) to be "vulnerable" countries.

Section 2. Country groups and pathways towards Universal Health Coverage

2.1 Country groups

Information on the current situation in countries is best known domestically, including current constraints and opportunities for moving forward. However, to ensure a realistic basis for our analysis, countries were classified into five groups based on publicly available data. We employed criteria that capture countries' current risk and disease burden, their current resource availability, and the effective use of those resources. We consider the following dimensions: (i) conflict/fragility; (ii) resource availability as measured by gross domestic income (GNI) per capita and/or gross domestic product (GDP) per capita[3]; (iii) health system capacity as measured by the current density of health workers as a proxy for service delivery readiness, and (iv) current health system performance as measured through skilled birth attendance coverage.

Table S2 provides an overview of the five country categories. The main purpose of the classification is to inform the modelled timing and duration of strategic investments. Countries belonging to lower level groups (C, V, HS1) are assumed to be unable to scale-up as rapidly as countries belonging to higher level groups (HS2, HS3) for many of the investments considered, due to the more limited absorptive capacity in their systems.[4]

Table S2. Country groups considered for the analysis

Type	Description	Criteria<br>(a) Conflict/fragility<br>(b) Resource availability[5]<br>o GNI/Capita in PPP<br>o GDP/Capita in PPP<br>(c) Service delivery readiness:<br>o HR density<br>(d) Current service delivery performance, as measured by skilled birth attendance coverage (SBA)
**Conflict-affected states (C)**	Countries with an internal or external conflict which considerably limits the state's ability to provide health services	• More than 10% of the population is affected by conflict (criteria a).
**Vulnerable countries (V)**	Countries with structural vulnerabilities, ranging from localized conflicts, a weak state apparatus, presence of external actors such as international humanitarian response structures, or recent health crises, which limit the state`s ability to provide health services	Countries with **vulnerable systems** that have one or more of the following characteristics:<br>• Recent health system crisis (criteria a)<br>• High score on the International Fragility Index[6] (criteria a)
**Health System category 1 (HS1)**	Countries with poor performance across health system functions. These countries require an engineering of their health system in order to build the foundations of strong health system institutions, and will thus require significant investments across the health system.	Countries have limited resources and low coverage of care.<br>• GNI (PPP) / GDP (PPP) per capita falls under 2,500 (b), AND<br>• Less than 2.28 health workers per 10,000 population (c), OR<br>• SBA<90% (criteria d)

[3] GDP data used when GNI data is not available.
[4] One of the main factors for absorptive capacity is the available health workforce which effectively sets the production frontier. Other criteria include conflict/fragility, governance, and past performance on public expenditure management.
[5] GDP/Capita PPP used when GNI/Capita PPP is unavailable. PPP = Purchasing Power Parity –adjusted dollars.
[6] Countries with a combined score of more than 43.5 out of 50, based on scores for five key components of the Fragility Index developed by the Fund For Peace. The five components are: demographic pressures, poverty and economic decline, limits to the provision of public services, inexistence of a security apparatus, and presence of external intervention.

**Health System category 2 (HS2)**	Countries have invested in the foundations of health systems but institutional performance is poor and there are challenges related to health system efficiency and access. There is scope for rapid health system scale-up to improve performance and move towards greater domestic financing sustainability.<br><br>This includes countries that:<br>• have limited resources but are performing well in terms of SBA coverage<br>• have fewer limitations on economic resources but face challenges with respect to health worker density<br>• have fewer limitations on economic resources but are doing less well on service coverage	Countries with a combination of criteria:<br><br>• Countries that are resource constrained (GNI-PPP per capita <2,500) but perform well on a representative indicator for complex care (SBA>90%), signalling service delivery readiness that allows for quick scale up for public service coverage, should resources be made available.<br><br>• Countries that are less resource constrained (GNI-PPP per capita >2,500) but where key health workforce availability is limited (HRH <2.28) , OR countries exceed the health workforce 2.28 benchmark but are doing less well on service coverage and delivery of complex services (SBA<90 %).
**Health System category 3 (HS3)**	Countries with mature health systems but in which there is an ongoing need to support health system transformation and reorient models of care to address emerging challenges and existing inequities.	This category includes:<br><br>• Countries with relatively high resource availability[7] defined as a GNI-PPP greater than 5,000, and high levels of delivery of complex care, defined as greater than 90% coverage of skilled birth attendance (criteria b, d).<br>• Countries with high resource availability defined as a GNI-PPP greater than 10,000 per capita (criteria b).

Table S3 presents the average and median values within each country group for economic and health systems resources, and current service delivery performance, as measured by Skilled Birth Attendance (SBA) and treatment of acute respiratory infection (ARI) in children.

It should be noted that the scale and scope of investments required are determined within the analytical model for each health system or service component, based on an account of the current situation as well as the anticipated system that countries will need to attain by 2030. There is therefore significant variation within each of the four groups as to what the additional investment requirements are, and what the additional associated costs would be.

[7] More than 10,000 GNI PPP/capita, or 10,000 GDP PPP/capita when data on GNI PPP/capita unavailable.

Table S3. Current resource availability and health system performance within the five country groups, average and median values [8]

			Economic resources	Health system resources		Service coverage	
Type		Number of countries	GDP/Capita [9]	Health workforce density per 1,000 population [10]	Number of Health Centers per 100,000 people [11]	Skilled Birth Attendance (%)[12]	Pneumonia treatment (%)[13]
**Conflict-affected states (C)**	4	Average	7,088	2.2	10.5	60.5	62.9
		Median	3,123	1.1	2.7	41.5	49.2
**Vulnerable systems (V)**	11	Average	1,637	0.4	10.6	50.9	39.8
		Median	1,626	0.5	10.5	45.2	38.8)
**HS1**	15	Average	2,002	0.6	10.6	50.1	24.4
		Median	1,529	0.5	9.3	58.0	34.3
**HS2**	16	Average	5,892	1.8	6.0	67.8	27.7
		Median	4,511	1.2	7.5	64.7	41.5
**HS3**	21	Average	13,611	4.3	16.8	98.6	67.2
		Median	13,262	4.5	12.1	98.6	59.0

Figure S1 presents the median values for each country group across a pair of service delivery and health system capacity indicators. We would expect an increasing trend for HS1, HS2 and HS3 countries. Health workforce density is significantly higher in HS3 countries than in the other categories. With respect to density of available infrastructure, measured by available health centres and health posts per 100,000 population, the results are more mixed, which can likely be both attributed to incomplete and unreliable data on infrastructure availability, as well as different service delivery models in different countries, where a very high number of health posts will distort this indicator. We therefore did not use facility density as a criterion to classify countries into the five groups.

In general, results for Conflict countries are more difficult to interpret since this category includes both low and middle income countries. Moreover, the latest available data may not represent the current situation in Conflict countries, which has most likely deteriorated since the data was collected.

[8] General note: average values are population weighted averages per group. Average and median values are based on countries for which data is available.
[9] US$,2014, World Bank.
[10] Doctors, nurses and midwifes per 1,000 population. Latest available data from WHO GHO.
[11] Number of health centers or health posts per 100,000 people. Available data from WHO GHO and national strategic national health sector plans (http://nationalplanningcycles.org/).
[12] Percentage (%) of births treated by skilled attendants. Latest available data from WHO GHO.
[13] Percentage (%) of children under five with symptoms of pneumonia given antibiotic treatment. Latest available data from WHO Global Health Observatory.

Figure S1. Availability of key infrastructure and health workforce per country group (median values)

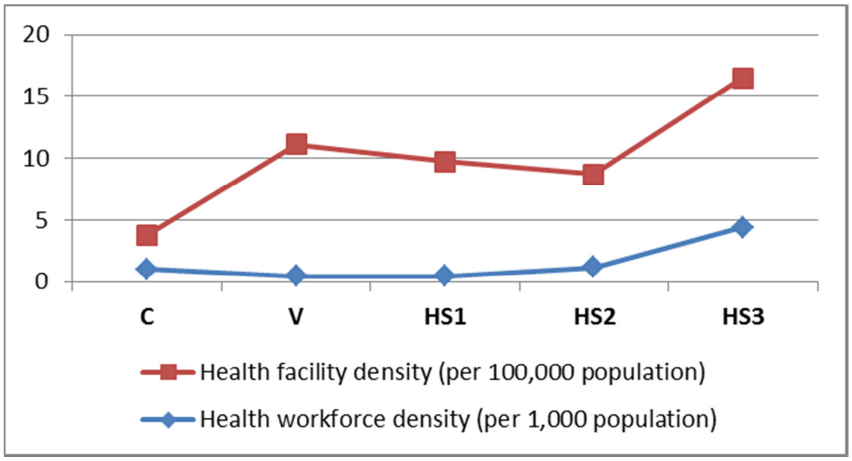

Facility density per 100,000 population. Includes health centers and health posts only.
Health workforce density: medical doctors, nurses, and midwifes per 1,000 population.

Meanwhile, data on management of acute respiratory infection in children indicates that on average, population coverage is higher in HS3 countries than in HS1 and HS2, and similarly coverage is higher in HS2 than in HS1 countries, as would be expected (Figure S2).

Figure S2. Current coverage of essential health services per country group (median values)

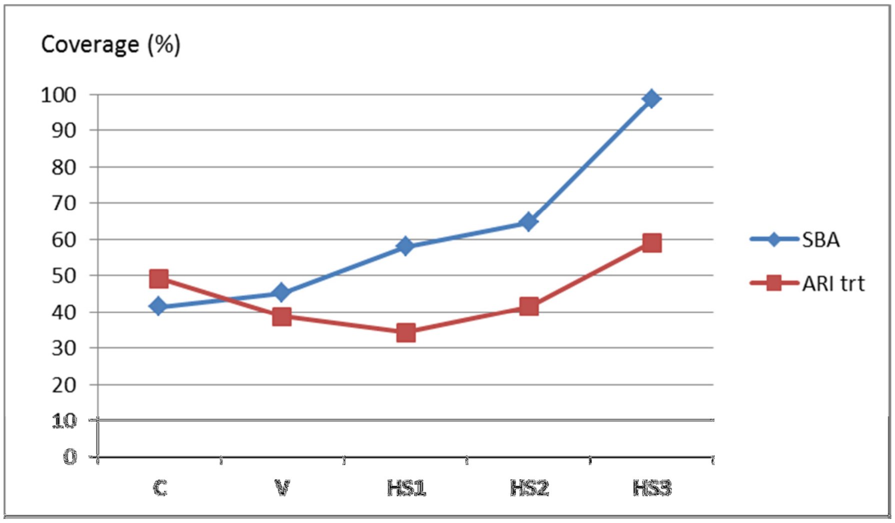

SBA: Skilled birth attendance; ARI trt: treatment coverage of acute respiratory infection in children [14]

[14] Percentage (%) of children under five with symptoms of pneumonia given antibiotic treatment; latest available data from WHO Global Health Observatory. http://www.who.int/gho/en/ Accessed 24 May 2016.

2.2. *Integrated health service delivery*

SDG 3 includes a broad health goal, “Ensure healthy lives and promote well-being for all at all ages”, and calls for achieving universal health coverage (UHC). The package of services to be provided as part of UHC is country-specific and evolves over time, in response to changes in epidemiology, consumer demand, resource constraints, and available technology. In terms of setting boundaries for a set of services to model for the purpose of analysis, we reviewed the disease/programme-specific targets under SDG 3 and other related SDGs (2 and 6) as well as the proposed tracer indicators for UHC service coverage.[15] We reviewed published guidance on essential health interventions and the list of services included under available disease-specific global strategies. We also consulted individual technical departments within WHO for each relevant area in order to obtain a list of recommended essential interventions.

Service delivery platforms: In recognition of the diversity of available guidance and technologies to prevent and treat health conditions, our analysis considers four service delivery platforms. The delivery platforms represent different modes for providing patients with information, counselling, essential preventive commodities, screening, diagnosis, treatment, and follow-up. We discuss the delivery platforms in terms of three characteristics of health services: discretionary vs standardised services, the level of intensity of the transaction involved between provider and patient, and asymmetry of information. Before describing the platforms, we describe these characteristics in brief.

Individualized vs standardized services: the concept of heterogeneous health services refer to those that are tailored to the individual and not necessarily provided to the population *en masse*. An example is caesarean sections, where doctors must exercise significant judgment on the aspects of the individual case in order to determine what to deliver and how. On the other end of the spectrum, standardized interventions are those that have great uniformity across patients. The latter tend to be recommended for and provided to a greater share of the population, without screening or diagnosis. Measles vaccination is a typical standardized intervention which is recommended for the entire population. Similarly, when a policy is implemented to have plain/standard packaging and/or large graphic health warnings on all tobacco packages, this is a standardized intervention which all people will benefit from and from which there is no “opting out”.

Intensity of transaction: transaction-intensive services are those that require a large amount of client-provider contact.[16] This includes services with repeated check-ups, such as management of more or less chronic conditions like antiretroviral treatment for HIV/AIDS, or management of non-chronic conditions that require extensive periods of health worker follow-up, such as management of severe acute malnutrition. It also includes those interventions where significant health worker time is required during a peak time (complex surgery). On the other hand, several interventions require very limited interaction with a provider, such as deworming.

***Asymmetry of information**:* For many health services, doctors and other caregivers hold considerably more information with regards to the recommended behaviours or actions to be adopted, than the patient does. For example, patients contracting a sexually transmitted infection will require medical assistance to diagnose and treat the infection. Many patients will have asymptomatic infections and will not be aware that they are infected, which can only be discovered through testing. On the other hand, there are other interventions for which there is very little asymmetry of information. For example, when bed nets are distributed, there is little doubt regarding their intended purpose or manner of use. Similarly, immunization campaigns carry a clear message to prevent illness, which is easily understood by the population.

With the above three characteristics in mind, we define four service delivery platforms, and assign health interventions accordingly. We fully recognise that the organisation and presentation of interventions by platforms does not imply that each intervention can only be delivered through one platform or at one service

[15] WHO (2015) Tracking universal health coverage: first global monitoring report.
[16] See World Development Report 2004.

delivery level. There exist a multitude of options for delivering services, and many may be simultaneously relevant. We also acknowledge that technologies may change over time, and that the characteristics of future service delivery platforms, or the preferred platform of specific interventions, may differ.

Within our model we use the organisation of services into platforms in order to assess the constraints associated with the provision of each type of health intervention, and the rate at which those constraints can be overcome. For presentation purposes, interventions are assigned to the platform where their delivery is considered most cost-effective.

Platform 1: Policy and population wide interventions. This platform focuses on policies and information communication that can be delivered to the population *en masse* at relatively low cost, to support changes in behaviours among risk groups in the population, whether for preventive purposes (i.e., reduce smoking, promote physical exercise, sleep under a mosquito net) or to ensure an appropriate response to a health problem (i.e., ensure that a child with diarrhoea takes oral rehydration salts and receives an increased intake of fluids).

Interventions included within this category are typically standardized, with low transaction intensity, and limited information asymmetry. A key characteristic is that they require little or no contact with a health provider, and thus do not place a burden on health worker time. Some interventions included within this package include distribution of commodities, i.e., insecticide treated bed nets[17].

Not all interventions within this category would follow the same pace of implementation. Many policy interventions require initial investments in regulatory frameworks to improve implementation capacity. Other interventions can however be scaled-up more rapidly (i.e., distribution of bed nets, with accompanying information campaigns).

Many interventions in this category, such as tobacco prevention policies and mass media campaigns for HIV/AIDS awareness, are highly cost-effective, and can be rapidly expanded at low cost in most countries, although they will require initial investment in capacity, to design and implement effective programmes, and institutions, to oversee their implementation.

This platform also includes services mainly funded and delivered by actors working outside the health sector, particularly those related to water, sanitation, and hygiene, and the reduction of indoor air pollution. The promotion of healthy behaviours related to environmental conditions is less reliant on health worker time, but heavily dependent on hardware investments in pipelines and equipment improving households' access to, and use of, safe water and clean cooking equipment.

Platform 2: Periodic schedulable and outreach services. This category includes services which are provided routinely and periodically. They may be provided periodically (for example mass distribution of drugs for deworming) or provided continuously but accessed at a certain pre-determined period from the perspective of the patient (such as in antenatal care or iodine supplementation). The key characteristic of these interventions is that they are standardized and have low levels of information asymmetry. They require user contact with health workers, but through brief and schedulable interventions. Because of their relatively high level of standardization, a number of interventions can be delivered through health workers with short training. Some interventions refer to the provision of counselling to certain patient groups – e.g., counselling parents to ensure appropriate nutrition for their children, or reaching out to injecting drug users to make sure they exchange needles safely.

In most settings – including resource constrained systems - services provided through this platform can be rapidly expanded. There are opportunities for rapid scale-up of preventive care through population-based approaches including community and outreach services, such as routine immunization campaigns and vector control for neglected tropical diseases.

[17] Because the actual correct utilisation of bed nets is highly dependent on effective communication around their use, and there is very limited information asymmetry with respect to the purpose of the nets, we have placed this intervention under platform 1.

For other interventions in this package, the recommendation remains for delivery at the health center level – e.g., delivery of new immunizations based on more recent technology, such as rotavirus and pneumococcal vaccine.

Platform 3: First level clinical services: This platform includes mainly services delivered through primary level health facilities. Compared to the first platforms, this platform includes individual health-care interventions that are specific to the patient's needs. This is the largest category of services within our model, and it covers a wide range of services with different characteristics. Typically, however, these services require more than a brief interaction with a health worker. They also require the health worker to have a certain level of skills and diagnostic tools, and therefore can be described to have a medium level of transaction intensity. Examples include treatment of sexually transmitted infections, treatment of TB, and treating and managing non-communicable diseases such as diabetes. One consideration for placing services in this category rather than in platforms 2 and 4 is the level at which they can be delivered. Many interventions can be delivered through a primary health care model, and many patients can be seen at health centers for these conditions. Care is more often than not tailored to specific patient needs (e.g., treatment of high blood pressure and diabetes), and with medium to high levels of information asymmetry – more often than not at the higher end, following the typical information asymmetry relationship between patients and health care providers.

Platform 4: Specialized care: Specialized care would typically be delivered by highly skilled health personnel, and rely on sound diagnostic and referral systems. Examples of interventions include diagnosis and treatment of cancer, management of obstructed labour, and management of severe acute malnutrition. These are health services requiring a significant amount of health worker time, and with high transaction intensity. Services in this category are typically highly individualized – e.g., identification and management of infertility. While information asymmetry is high, many services are also discretionary in nature, meaning that patients can opt in or out, and agree on the treatment in a participatory process (e.g., cancer treatment).

Table S4. A summary of the four service delivery platforms

Service delivery platform	Typical service characteristics	Implementation model	Typical interventions (examples)
**Policy and population wide interventions**	• Standardized<br>• Low transaction intensity (require little or no contact with a health provider)<br>• Limited information asymmetry.	Policies driven jointly with other sectors, such as Ministries of Finance, for example fiscal policies to make harmful and unhealthy products less affordable.<br><br>Changing consumer products – e.g., large graphic health warnings and plain packaging on tobacco products–is a quick-win policy to communicate health messages to large populations, while the production of these packages is the obligation of the tobacco industry.<br><br>Mass media campaigns and community mobilization interventions can be rapidly scaled-up since they are less reliant on health system strengthening. These can make standardized products (such as bed nets) universally available.	Increase excise taxes and prices on tobacco products, alcohol and sugar-sweetened beverages.<br><br>Large graphic health warnings and plain packaging on all tobacco products.
		Certain policy interventions require a lag time during which institutions are built up, after which implementation rapidly expands.	Bed nets for malaria. Restrictions on availability of retailed alcohol, and implementation of drunk driving laws.
**Periodic schedulable and outreach services**	• Standardized<br>• Low transaction intensity (require brief contact with a health provider)<br>• Limited information asymmetry	Rapidly scaled-up as less reliant on health system strengthening. Makes use of outreach into the community, but also health centre level delivery.	Immunization, Neglected Tropical Disease programmes.
**First level clinical services**	• Individualized<br>• Medium transaction intensity<br>• Medium to high information asymmetry.	Primary health care platform where service coverage relies on a successively strengthened health system, with functioning and accessible facilities that are adequately staffed with health workers providing quality outpatient care.	Treatment of pneumonia in children,<br>Management of sexually transmitted infections,<br>Management of depression.
**Specialized services**	• Individualized<br>• High transaction intensity<br>• High information asymmetry.	Expansion of service coverage only happens after the build-up of specialized resources, which may take longer to acquire and will rely more heavily on investments in the health system.	Skilled birth attendance,<br>Surgery for trauma care and fractures.

Table S5. Essential Interventions organized into Service Delivery Platforms

Platform number (1-4)	Intervention number	Intervention name	Programme	Delivery levels within modelled approach[18]	Tool used to model costs and impact
Platform 1. Policy and population wide interventions					
1	1	Increase excise taxes and prices on tobacco products.	NCD	National policy	OHT / Excel (*)
1	2	Implementation of plain/standardized packaging and/or large graphic health warnings on all tobacco packages	NCD	National policy	OHT / Excel (*)
1	3	Comprehensive ban of tobacco advertising, promotion and sponsorship, including cross-border advertising and on modern means of communication	NCD	National policy	OHT / Excel (*)
1	4	Elimination of exposure to second-hand tobacco smoke in all indoor workplaces, public places, public transport, and in all outdoor mass-gathering places	NCD	National policy	OHT / Excel (*)
1	5	Implement effective mass media campaigns that educate the public about the harms of smoking/tobacco use and second hand smoke	NCD	National policy	OHT / Excel (*)
1	6	Provision of cost-covered, effective and population-wide support (including brief advice, national toll-free quit line services and mCessation) for tobacco cessation to all those who want to quit	NCD	National policy	OHT / Excel (*)
1	7	Hazardous alcohol use: Enforce restrictions on availability of retailed alcohol (**)	NCD	National policy	Excel
1	8	Hazardous alcohol use: Enforce restrictions on alcohol advertising (**)	NCD	National policy	Excel
1	9	Hazardous alcohol use: Enforce drunk driving laws (sobriety checkpoints) (**)	NCD	National policy	Excel
1	10	Hazardous alcohol use: Raise taxes on alcoholic beverages (**)	NCD	National policy	Excel
1	11	Physical inactivity: Implement public awareness and motivational communications for physical activity, including mass media campaign for physical activity behaviour change	NCD	National policy	OHT / Excel (*)
1	12	Sodium: Surveillance	NCD	National policy	OHT / Excel (*)
1	13	Sodium: Harness industry for reformulation	NCD	National policy	OHT / Excel (*)
1	14	Sodium: Adopt standards: Front of pack labelling	NCD	National policy	OHT / Excel (*)
1	15	Sodium: Adopt standards: Strategies to combat misleading marketing	NCD	National policy	OHT / Excel (*)
1	16	Sodium: Knowledge: Education and communication	NCD	National policy	OHT / Excel

[18] Non-health indicated for interventions where all or a share of total costs are assumed to fall under other sectors than health.

					(*)
1	17	Sodium: Environment: Salt reduction strategies in community-based eating spaces	NCD	National policy	OHT / Excel (*)
1	18	Diet: Complete elimination of industrial trans fats through the development of legislation banning their use in the food chain	NCD	National policy	OHT / Excel (*)
1	19	Mass media (HIV/AIDS)	HIV/AIDS	National policy	OHT
1	20	Community mobilization (HIV/AIDS)	HIV/AIDS	Community	OHT
1	21	Distribution of long lasting insecticide treated bed nets	Malaria	Community, first level facility	OHT
1	22	Management of diarrhoea using Oral Rehydration Salts, zinc and increased intake of fluids	RMNCH	Community, first level facility	OHT
1	23	Use of improved water source within 30 minutes	WASH	Community, non-health	OHT/ Excel (*)
1	24	Use of water connection in the home	WASH	Community, non-health	OHT/ Excel (*)
1	25	Improved excreta disposal (latrine/toilet)	WASH	Community, non-health	OHT/ Excel (*)
1	26	Hand washing with soap	WASH	Community, non-health	OHT/ Excel (*)
1	27	Hygienic disposal of children's stools	WASH	Community, non-health	OHT/ Excel (*)
1	28	Promotion of the use of clean fuels and technologies for cooking (**)	ENV	Community, non-health	Excel
**Platform 2: Periodic outreach services**					
2	29	Measles vaccine	EPI	Outreach, first level facility	OHT
2	30	Polio vaccine	EPI	Outreach, first level facility	OHT
2	31	HPV vaccine	EPI	Outreach, first level facility	OHT
2	32	Rotavirus vaccine	EPI	First level facility	OHT
2	33	Pentavalent vaccine	EPI	First level facility	OHT
2	34	DPT vaccination	EPI	First level facility	OHT
2	35	Hib vaccine	EPI	First level facility	OHT
2	36	Hep B vaccine to prevent liver cancer	EPI	First level facility	OHT
2	37	BCG vaccine	EPI	First level facility	OHT
2	38	Pneumococcal vaccine	EPI	First level facility	OHT
2	39	Yellow Fever vaccine (**)	EPI	Outreach, first level facility	Excel

2	40	Meningitis vaccine (**)	EPI	Outreach, first level facility	Excel
2	41	Japanese Encephlopathy Vaccine (**)	EPI	Outreach, first level facility	Excel
2	42	Neglected Tropical Diseases: Preventive chemotherapy (PC) including post-PC surveillance (**)	NTD	Community, outreach	Excel
2	43	Neglected Tropical Diseases: Vector management (**)	NTD	Community, outreach	Excel
2	44	Neglected Tropical Diseases: Disease management including active case finding (**)	NTD	Community, outreach, first level facility	Excel
2	45	Vector control for malaria	Malaria	Community	Excel
2	46	Chemoprevention in vulnerable populations (**)	Malaria	Outreach	Excel
2	47	Clean practices and immediate essential newborn care (home)	RMNCH	Community	OHT
2	48	Family planning	RMNCH	Community, outreach, first level facility	OHT
2	49	Outreach to injecting drug users	HIV/AIDS	Community, outreach	OHT
2	50	Needle exchange for injecting drug users	HIV/AIDS	Community, outreach	OHT
2	51	Interventions focused on female sex workers	HIV/AIDS	Community, outreach	OHT
2	52	Interventions focused on men who have sex with men	HIV/AIDS	Community, outreach	OHT
2	53	Condoms for HIV/AIDS	HIV/AIDS	Community	OHT
2	54	Iodine supplementation for pregnant women and for children (**)	Nutrition	Community, first level facility	Excel
2	55	Daily iron and folic acid supplementation (pregnant women)	Nutrition	Community, first level facility	OHT
2	56	Daily Iron folic acid, postpartum, anaemic women (**)	Nutrition	Community, first level facility	OHT
2	57	Breastfeeding counselling and support	Nutrition	Community, outreach, first level facility	OHT
2	58	Complementary feeding counselling and support	Nutrition	Community, first level facility	OHT
2	59	Nurturing care counselling for early child development	RMNCH	Community, first level facility	Excel
2	60	Support for maternal depression	RMNCH	Community, first level facility	Excel
2	61	Home fortification of food with multiple micronutrient powders (children 6-23 months)	Nutrition	Community, first level facility	OHT

2	62	Vitamin A supplementation in infants and children 6-59 months	Nutrition	Community, first level facility	OHT
2	63	Intermittent iron supplementation in children	Nutrition	Community	OHT
2	64	Daily iron supplementation for children 6 to 23 months (where anaemia is >= 40%)	Nutrition	Community	OHT
2	65	Management of moderate acute malnutrition (children)	Nutrition	Community, first level facility	OHT
2	66	Feeding counselling and support for infants and young children in emergency situations (**)	Nutrition	Outreach	OHT
2	67	Offer to help quit tobacco use: Brief intervention	NCD	First level facility	OHT/Excel (*)
2	68	Screening and brief intervention for hazardous and harmful alcohol use	NCD	First level facility	OHT/Excel (*)
2	69	Physical inactivity: Brief advice as part of routine care	NCD	First level facility	OHT/Excel (*)
2	70	Basic palliative care for breast, cervical and colorectal cancer (**)	NCD/cancer	Community, outreach, hospital outpatient	Excel
Platform 3: **First level clinical services**					
3	71	Safe abortion	RMNCH	First level facility, hospital outpatient, hospital inpatient	OHT
3	72	Post-abortion case management	RMNCH	First level facility, hospital outpatient, hospital inpatient	OHT
3	73	Ectopic case management (medical)	RMNCH	Hospital inpatient	OHT
3	74	Tetanus toxoid immunization (pregnant women)	RMNCH	First level facility	OHT
3	75	Syphilis detection and treatment (pregnant women)	RMNCH	First level facility	OHT
3	76	Basic antenatal care (4 visits)	RMNCH	First level facility, hospital outpatient	OHT
3	77	Hypertensive disorder case management	RMNCH	First level facility, hospital outpatient, hospital inpatient	OHT
3	78	Management of pre-eclampsia (Magnesium sulphate)	RMNCH	First level facility, hospital outpatient, hospital inpatient	OHT
3	79	Labor and delivery management - normal delivery	RMNCH	First level facility, hospital outpatient, hospital inpatient	OHT
3	80	Active management of the 3rd stage of labour	RMNCH	First level facility, hospital outpatient, hospital inpatient	OHT
3	81	Management of eclampsia (Magnesium sulphate)	RMNCH	First level facility, hospital outpatient, hospital inpatient	OHT

3	82	Neonatal resuscitation (institutional)	RMNCH	First level facility, hospital outpatient, hospital inpatient	OHT
3	83	Treatment of local infections (Newborn)	RMNCH	First level facility, hospital outpatient	OHT
3	84	Kangaroo mother care	RMNCH	First level facility, hospital outpatient	OHT
3	85	Feeding counselling and support for low-birth-weight infants (**)	RMNCH	Community, First level facility, hospital outpatient,	OHT
3	86	Antibiotics for preterm premature rupture of membranes (pPRoM)	RMNCH	First level facility, hospital outpatient	OHT
3	87	Maternal Sepsis case management	RMNCH	First level facility, hospital outpatient	OHT
3	88	Newborn sepsis - Injectable antibiotics	RMNCH	First level facility	OHT
3	89	Clean postnatal practices	RMNCH	First level facility	OHT
3	90	Mastitis	RMNCH	First level facility, hospital outpatient	OHT
3	91	Chlorhexidine for cord care	RMNCH	First level facility, hospital outpatient	OHT
3	92	Treatment of syphilis	RMNCH	First level facility, hospital outpatient	Excel
3	93	Treatment of gonorrhoea (**)	RMNCH	First level facility, hospital outpatient	Excel
3	94	Treatment of chlamydia (**)	RMNCH	First level facility, hospital outpatient	Excel
3	95	Treatment of trichomoniasis (**)	RMNCH	First level facility, hospital outpatient	Excel
3	96	Treatment of lower abdominal pain and Pelvic Inflammatory Disease (PID) - lower abdominal pain (**)	RMNCH	First level facility, hospital outpatient	Excel
3	97	Treatment of urinary tract infection (UTI) (**)	RMNCH	First level facility, hospital outpatient	Excel
3	98	Vitamin A supplementation for treatment of xerophthalmia in women of reproductive age (**)	RMNCH	First level facility	OHT
3	99	Vitamin A supplementation for treatment of xerophthalmia in children (**)	RMNCH	First level facility	OHT
3	100	Pneumonia treatment (children)	RMNCH	First level facility	OHT
3	101	Antibiotics for treatment of dysentery in children	RMNCH	First level facility, hospital outpatient	OHT
3	102	Vitamin A for measles treatment (children)	RMNCH	First level facility	OHT
3	103	Intermittent preventive treatment of malaria in pregnancy (iptp)	Malaria	First level facility	OHT

3	104	Malaria diagnosis and treatment (children under five)	Malaria	First level facility	OHT
3	105	Malaria diagnosis and treatment (population aged 5 years and above, including pregnant women)	Malaria	First level facility	OHT
3	106	TB: first line	TB	First level facility	Excel
3	107	TB: second line	TB	First level facility	Excel
3	108	Collaborative TB/HIV activities, and management of co-morbidities	TB	First level facility	Excel
3	109	TB: diagnostic	TB	First level facility	Excel
3	110	Drug substitution for injecting drug users	HIV/AIDS	First level facility	OHT
3	111	Voluntary counselling and testing	HIV/AIDS	First level facility	OHT
3	112	Male circumcision	HIV/AIDS	First level facility	OHT
3	113	Prevention of mother-to-child transmission (PMTCT)	HIV/AIDS	First level facility, hospital outpatient	OHT
3	114	Post-exposure prophylaxis	HIV/AIDS	First level facility, hospital outpatient	OHT
3	115	ART (Second-Line Treatment) for adults	HIV/AIDS	First level facility, hospital outpatient	OHT
3	116	Paediatric ART	HIV/AIDS	First level facility, hospital outpatient	OHT
3	117	Cotrimoxazole for children	HIV/AIDS	First level facility, hospital outpatient	OHT
3	118	HIV/AIDS service package for transgender populations (**)	HIV/AIDS	Outreach	Excel
3	119	HIV/AIDS service package for prisoners (**)	HIV/AIDS	Outreach	Excel
3	120	Pre-exposure prophylaxis (PrEP) (**)	HIV/AIDS	First level facility	Excel
3	121	Intermittent iron-folic acid supplementation (menstruating women where anaemia is public health problem)	Nutrition	Community, outreach, first level facility, hospital outpatient	OHT
3	122	Intermittent iron and folic acid supplementation (non-anaemic pregnant women) (**)	Nutrition	First level facility, hospital outpatient	OHT
3	123	Vitamin A supplementation in pregnant women	Nutrition	First level facility, hospital outpatient	OHT
3	124	Calcium supplementation for prevention and treatment of pre-eclampsia and eclampsia	Nutrition	First level facility, hospital outpatient	OHT
3	125	Nutritional care and support (HIV+ pregnant and lactating women) (**)	Nutrition	First level facility	OHT
3	126	Nutritional care and support for pregnant and lactating women in emergencies	Nutrition	First level facility	OHT
3	127	Intermittent FAF, postpartum, non-anemic pregnant women (**)	Nutrition	First level facility	OHT

3	128	Screening for risk of CVD/diabetes	NCD	First level facility	OHT
3	129	Follow-up care for those at low risk of CVD/diabetes (absolute risk: 10-20%)	NCD	First level facility	OHT
3	130	Treatment for those with very high cholesterol but low absolute risk of CVD/diabetes (< 20%) OHT	NCD	First level facility	OHT
3	131	Treatment for those with high blood pressure but low absolute risk of CVD/diabetes (< 20%)	NCD	First level facility	OHT
3	132	Treatment for those with absolute risk of CVD/diabetes 20-30%	NCD	First level facility	OHT
3	133	Treatment for those with high absolute risk of CVD/diabetes (>30%)	NCD	First level facility, hospital outpatient	OHT
3	134	Treatment of cases with rheumatic heart disease (with benzathine penicillin)	NCD	First level facility, hospital outpatient	OHT
3	135	Standard glycemic control	NCD	First level facility, hospital outpatient	OHT
3	136	Intensive glycemic control	NCD	First level facility, hospital outpatient	OHT
3	137	Neuropathy screening and preventive foot care	NCD	First level facility, hospital outpatient	OHT
3	138	Screening and Treat pre-cancerous lesions (Cervical cancer: VIA, HPV+VIA)	NCD/cancer	First level facility, hospital outpatient	Excel
3	139	Colorectal Cancer screening	NCD/cancer	Community, First level facility, hospital outpatient	Excel
3	140	Post-cancer surveillance (breast, cervical, colorectal)	NCD/cancer	First level facility, hospital outpatient	Excel
3	141	Extended palliative care for breast cancer for breast, cervical and colorectal cancer	NCD/cancer	Community, outreach, hospital outpatient	Excel
3	142	Asthma: Inhaled short acting beta agonist for intermittent asthma	NCD	First level facility, hospital outpatient	OHT
3	143	Asthma: Low dose inhaled beclometasone + short-acting beta 2-agonists (SABA)	NCD	First level facility, hospital outpatient	OHT
3	144	Asthma: High dose inhaled beclometasone + short-acting beta 2-agonists (SABA)	NCD	First level facility, hospital outpatient	OHT
3	145	Chronic obstructive pulmonary disease (COPD): Smoking cessation	NCD	First level facility, hospital outpatient	OHT
3	146	Chronic obstructive pulmonary disease (COPD): Inhaled salbutamol	NCD	First level facility, hospital outpatient	OHT
3	147	Chronic obstructive pulmonary disease (COPD): Low-dose oral theophylline	NCD	First level facility, hospital outpatient	OHT
3	148	Chronic obstructive pulmonary disease (COPD): Ipratropium inhaler	NCD	First level facility, hospital outpatient	OHT
3	149	Basic psychosocial treatment for anxiety disorders	MNS	First level facility,	OHT

		(mild cases)		hospital outpatient	
3	150	Basic psychosocial treatment and anti-depressant medication for anxiety disorders (moderate-severe cases)	MNS	First level facility, hospital outpatient	OHT
3	151	Basic psychosocial treatment for mild depression	MNS	First level facility, hospital outpatient	OHT
3	152	Basic psychosocial treatment and anti-depressant medication of first episode moderate-severe cases	MNS	First level facility, hospital outpatient	OHT
3	153	Intensive psychosocial treatment and anti-depressant medication of first episode moderate-severe cases	MNS	First level facility, hospital outpatient	OHT
3	154	Basic psychosocial support and anti-psychotic medication	MNS	First level facility, hospital outpatient	OHT
3	155	Intensive psychosocial support and anti-psychotic medication	MNS	First level facility, hospital outpatient	OHT
3	156	Basic psychosocial treatment, advice, and follow-up for bipolar disorder, plus mood-stabilizing medication	MNS	First level facility, hospital outpatient	OHT
3	157	Intensive psychosocial intervention for bipolar disorder, plus mood-stabilizing medication	MNS	First level facility, hospital outpatient	OHT
3	158	Basic psychosocial support, advice, and follow-up, plus anti-epileptic medication	MNS	First level facility, hospital outpatient	OHT
**Platform 4: Specialized care**					
4	159	Labor and delivery management - emergency obstetric care	RMNCH	Hospital inpatient	OHT
4	160	Pre-referral management of labor complications	RMNCH	First level facility, hospital outpatient	OHT
4	161	Management of obstructed labor	RMNCH	Hospital inpatient	OHT
4	162	Antenatal corticosteroids for preterm labor	RMNCH	Hospital inpatient	OHT
4	163	Induction of labor (beyond 41 weeks)	RMNCH	Hospital inpatient	OHT
4	164	Newborn sepsis - Full supportive care	RMNCH	Hospital inpatient	OHT
4	165	Treatment of postpartum hemorrhage	RMNCH	First level facility, hospital inpatient	OHT
4	166	Treatment of severe illness in children (diarrhea, pneumonia, malaria)	RMNCH	Hospital inpatient	OHT
4	167	Management of severe malnutrition (children)	Nutrition	Community, First level facility, hospital inpatient	OHT
4	168	Retinopathy screening and photocoagulation	NCD	First level facility, hospital outpatient	OHT
4	169	Treatment of new cases of acute myocardial infarction (AMI) with aspirin	NCD	Hospital outpatient	OHT
4	170	Treatment of cases with established ischaemic heart disease (IHD) and post MI	NCD	Hospital outpatient	OHT

4	171	Treatment for those with established cerebrovascular disease and post stroke	NCD	Hospital outpatient	OHT
4	172	Mammography	NCD/cancer	Hospital outpatient	Excel
4	173	Cervical cancer treatment: stage 1 to stage 4	NCD/cancer	Hospital inpatient	Excel
4	174	Colorectal cancer treatment: stage 1 to stage 4	NCD/cancer	Hospital inpatient	Excel
4	175	Breast cancer treatment: stage 1 to stage 4	NCD/cancer	Hospital inpatient	Excel
4	176	Asthma: Theophylline + High dose inhaled beclometasone + SABA	NCD	First level facility, hospital outpatient	OHT
4	177	Asthma: Oral Prednisolone + Theophylline + High dose inhaled beclometasone + SABA	NCD	First level facility, hospital outpatient	OHT
4	178	COPD: Exacerbation treatment with antibiotics	NCD	First level facility, hospital outpatient	OHT
4	179	COPD: Exacerbation treatment with oral prednisolone	NCD	First level facility, hospital outpatient	OHT
4	180	COPD: Exacerbation treatment with oxygen	NCD	First level facility, hospital outpatient	OHT
4	181	Intensive psychosocial treatment and anti-depressant medication for anxiety disorders (moderate-severe cases)	MNS	First level facility, hospital outpatient	OHT
4	182	Intensive psychosocial treatment and anti-depressant medication of recurrent moderate-severe cases on an episodic basis	MNS	First level facility, hospital outpatient	OHT
4	183	Intensive psychosocial treatment and anti-depressant medication of recurrent moderate-severe cases on a maintenance basis	MNS	First level facility, hospital outpatient	OHT
4	184	Surgical and trauma care (***)	Surgery	Hospital outpatient, hospital inpatient	Excel
Additional programmatic interventions incl. activities addressing socioeconomic determinants					
	185	Cash transfers for girls in hyper-endemic countries with low rates of secondary school enrolment (**)	HIV/AIDS	Non-health	Excel
	186	Cash transfer to poor women to deliver in facilities (**)	RMNCH	National level	Excel
	187	Programme support costs include training, monitoring, supervision, programme administration costs. (**)	ENV, EPI, HIV/AIDS, NCD, Malaria, MNS, NTD, Nutrition, RMNCH, Surgery, TB	National level	Excel

Notes to table: ENV= Environmental health; EPI = Expanded Program on Immunization, MNS = Mental Health and Substance Use; NCD = Non Communicable Disease; NTD= Neglected Tropical Diseases, OHT = OneHealth Tool, RMNCH = Reproductive, Maternal, Child and Newborn Health.

(*) Health impact was projected within the OHT projections, while costs were modelled in Excel. (**) No health impact modelling directly associated with this intervention. (***) Costs for surgical and trauma care is not modelled on a bottom-up patient perspective but rather from a health systems perspective ensuring that the necessary resources are made available. Health impact is not estimated.

2.3. Scenarios towards UHC

Given the uncertainty around the current capacity of health systems to absorb additional resources, we have modelled two scenarios with differing levels of ambition. Firstly, we worked with experts in each technical area to interpret global targets and their implications, in order to inform an ***ambitious scenario*** towards reaching global targets by 2030. The ambitious scenario considers strengthening health systems towards global benchmarks, and an accompanying expansion of the full package of services towards 95% coverage for most country categories, albeit at different speeds. It implies strengthening the foundations and institutions within health systems to enable these to support models of care that provide responsive, quality health services. It entails addressing six essential gaps (Box S1), by modelling investments towards attainment of benchmarks within each respective health system building block, where examples include attaining targets of facility density within the infrastructure component, and attaining high governance scores within the component for governance and regulation.

However, while global best practice targets can be, and have been, set for where countries should strive to be in 2030, our model recognizes that not all countries may fully achieve these targets.

Therefore, to illustrate the advancement that can be made under a more resource constrained scenario, we designed a ***progress scenario*** which models progress towards global targets whilst taking into account limits on absorptive capacity and health systems in distress. The purpose of the progress scenario is to illustrate a scenario where progress towards UHC will not occur evenly on all fronts, and where not all of the SDG targets may be met by 2030, but where significant progress can still be made, in particular through scaling up the provision of health interventions through the lower level service delivery platforms (policy, population-wide, and periodic schedulable and outreach delivery).

**Box S1. Addressing six essential gaps for health systems** [19]
• Financing: Invest in financial engineering to build a unified and transparent financial management system and procurement procedures, ensuring secure and transparent financial flows and enhancing accountability
• Health workforce: Invest in pre-service education for the PHC workforce, especially education pathways of six months to three years, with the parallel development of deployment and retention strategies in rural and remote areas
• Pharmaceuticals/medical products: Invest in supply chains, diagnostic facilities, stocks
• Health information: Invest in unified underlying health information systems, including surveillance
• Governance: invest in local health governance systems through district health management (including supervision, monitoring, performance management, health facility management committees, etc.) and community engagement
• Service delivery: invest in basic infrastructure and equipment

[19] WHO (2016), FIT – Foundations, Institutions, Transformation. Information Brochure, Department of Health Systems Governance and Financing.

In the "Ambitious" scenario, gaps are significantly closed by 2030, such that systems attain the aspirational targets identified and require their corresponding level of resource use. In the "Progress" scenario, substantial advancement is obtained, but there will still be a gap remaining for many countries by 2030 in most areas. The progress scenario may be considered by some to be a more realistic scenario which considers the limited capacity in many settings to absorb new funding and efficiently translate this into equitable service delivery.

Both scenarios illustrate the need for countries to proceed in a step-wise fashion. In both scale-up scenarios, fragile states will require a certain period of stability before frontloaded capital investments in (re)constructing health systems can take place. Moreover, as discussed above, service provision can be scaled up faster in certain delivery platforms than in others.

In both scenarios, health systems are modelled to be scaled up towards set benchmarks by 2030. The modelling for the three most resource intensive health system components (health workforce, infrastructure, and supply chain) is interlinked and closely related to the scope of services provided. Other health system investments (health information systems, Emergency Risk Management, governance and health financing) are more independent of the service package and relate to strengthening institutions. Table S6 provides an overview of the components that are modelled within the analysis and differences across the two scenarios.

Table S6. Investments to transform health systems

Strategic investment area	Ambitious Scenario	Progress Scenario
**Health workforce**: This component builds on the recommendation by the Global Strategy on Human Resources for Health that a ratio of 4.45 health workers per 1,000 population *represents a density fit for purpose for a transformed workforce to reach the SDGs.* Drawing upon the model developed for the Global Strategy, targets for health worker density per population are set by country, taking into account rural-urban population distributions.<br><br>Different service delivery platforms have different health workforce requirements, which are taken into account by our model. For instance, the necessary number of health workers who deliver outreach services based in rural centers varies in line with the projected % of rural population in each country. The scale-up of the workforce is matched to the facility infrastructure scale-up.<br><br>Taking into account current pre-service training capacity, we model the number of additional health workers expected to be added to the workforce each year (assuming that doctors require five years of training, new doctors needed to close an SDG target related gap only enter the labor market in 2021; similarly nurses and other cadres of workers are added in 2019 and 2017, respectively).<br><br>Our analysis also estimates the health workforce required to deliver health services as part of the 5 service delivery platforms outlined in Figure 1, using a bottom-up approach built into the OneHealth Tool , whereby each intervention is associated with a specific health worker provider time.	Costs relate to the additional health workers employed, assuming that the difference between the current country baseline densities and the 2030 targets will be entirely closed by 2030. It also includes continued in-service training, including all hazard training (but whose costs are part of Emergency Risk Management below).<br><br>The modelled increase in workforce relates both to the modelled expansion within the current pre-service education system, as well as additional production methods that need to be put in place in order to close the gap.	The progress scenario assumes a slower pace in additional production methods.<br><br>The anticipated expansion within the current pre-service education system remains the same as in the ambitious scenario, but efforts to close the gap are more modest (two-thirds of the gap closed). However, the production of "other cadres", who are key in providing services in service delivery platforms 1 and 2 are scaled up to close 90% of the gap by 2030, reflecting the service provision targets.
**Infrastructure and equipment**: Targets take into account the health services to be provided as part of each service delivery platform, as well as projected future population growth and rural-urban migration. Costs include the construction of new facilities, their equipment, and the recurrent costs that these will accrue, including maintenance. Vehicle costs are also considered, including the referral chain i.e., ambulances and drivers. Additionally, we model improvements in making water and power lines available in those facilities not yet connected,	Costs relate to health centers built to reach targets of 1 per 12000 population (urban)/ 6000 population (rural); District hospitals (1 per 100,000 urban and 1 per 50,000 rural population) and Provincial hospitals (1 per 100,000 population). Required	The progress scenario follows the same benchmark as the ambitious scenario, but it is considered that reaching the targets would take more than 15 years, and

in order to expand quality of care and access to basic services. Finally, we include investments for both new facilities and a proportion of existing facilities to meet safe hospital standards, taking actions to promote the resilience of new and existing hospitals and other health facilities.[(1)]	equipment and recurrent costs are estimated per facility type, including vehicle costs, but do not include single use medical devices, such as syringes or sutures, nor implantables or assistive devices. Due to the complex situations and limited years to build facilities, post-conflict and vulnerable countries (C and F1) are modelled as only closing 80 and 90% of the gap, respectively, in required health facilities. The connection of facilities to utility systems include only the additional recurrent cost of utility bills, and not the cost of connecting water, sanitation lines to facilities.	that the benchmarks will not be fully attained by 2030. The gap between the current and the needed number of facilities is closed by two-thirds by 2030, except for health centers, where 90% of the gap is closed by 2030.
**Supply chain**: Costs are modelled based on the estimated additional volume and value of the consumables transported through the system, which is determined by the commodities required to provide the entirety of services modelled as being delivered in our model.<br><br>Supply chain estimates come from the numbers of commodities, including medicines and medical devices, related to delivering all health interventions across the various service delivery platforms. This does not include shipping or insurance costs, but does include in country recurrent costs, construction of new warehouses, new trucks, and buffer stock. The recurrent cost of the supply chain is considered to be a cost fraction of the value of commodities being sent through the system, determined by a variety of factors, including population density, and logistics scores from the Wold Bank Logistics Performance Index.	Fixed and recurrent costs both relate to the volume of consumables transported through the system, by country and year, and thus are directly linked to the service targets within the model.	The progress scenario entails a lower number of commodities and thus lower resource needs.
For temperature-sensitive vaccines we specifically estimate costs related to cold chain, which are determined by estimating the cold space required to store the volumes of vaccines required to be able to provide immunization services in the subsequent year, as the storage capacity needs to be in place before mobilization and supplies of vaccines can be increased.	For cold chain estimates, the infrastructure needs for each country required to deliver the vaccine coverages of the ambitious scenario are identfied, estimating a gap of cold chain capacity. This gap is filled over time with new facilities scaled up at the same speed as similar-complexity health facilities. Similarly, the facilities and their cold storage equipment currently in existence in the 67 countries are deemed to be in need of upgrading between now to 2030, to incorporate new technology and replace outdated equipment. Almost all of this replacement is expected to fall under planned, continued government expenditure, but it is estimated that a small group of low income countries will need to raise additional resources to finance the replacement of 20% of their existing equipment.	The progressive scenario will follow the ambitious scenario and will just calculate a smaller capacity gap, based on the progressive scenario`s lower vaccination coverage levels. However, it should be noted that the countries that require additional resources to replace 20% of their cold storage equipment will still require the same resources in this scenario, as the need to upgrade the existing scenario, and its urgency and need, remains the same.

**Governance**: The necessary activities of a health system required to improve its governance structure, measured as improving a country`s "Country Policy and Institutional Assessment" (CPIA) Score[20]. These include strategic planning, consensus building, regulation and accreditation.	The gap between a countries current CPIA score and an ideal score of 6 is identified, whereby the activities related to governance are carried out so that a country`s score is increased every other year, reaching the highest level of 6 in every country by 2030.	This scenario is similar to the ambitious scenario, but sees countries only improve their governance to reach a CPIA score of 5.
**Health information systems:** we estimate the resources required to build up resilient information systems across the sector. This includes a financial information system, a health workforce information system, a public health institute, an information system division in the Ministry of Health, a facility-based information system and the periodic occurrence of surveys. This includes specialized health workers, equipment, and recurrent costs and governance-related activities. Scale up of the facility-based information system is linked to the scale up of facilities.	Following the Roadmap for Health Measurement for the Post-2015 Agenda, the ambitious scenario outlines a series of activities and capabilities a fully functioning health information system should have, Several components are linked to the building or refurbishment of facilities, while others follow a categorization of information systems that transition through various levels to an uppermost level of health information system development.	The components that are linked to health facilities scale up line with their construction or repair in this scenario. Where components follow a separate scale up pattern of progression, the speed of improvement or scale up is reduced.
**Health financing**: this component estimates the resources required to improve health financing towards achieving Universal Health Coverage, through strengthening the purchasing functions of social health insurance institutions and Ministries of Health who have public service provision[21].	After an assessment of countries that have either recently started or will shortly start reforming their health financing systems, it was estimated that countries should be spending 1 to 2% more of their General Government Health Expenditure (GGHE) to strengthen the administrative portions of SHIs and MoHs in order to achieve more effective reform of their health financing functions.	As the resources required are based on estimated countries changes in GGHE, the progressive scenario applies the same methodology to a less ambitious projection of GGHE.
**Emergency Risk Management and post emergency and conflict relief**:<br>We estimate the resources required for preparing for, and responding to, health emergencies and conflict. This includes three main components:<br><br>• *Emergency Preparedness, risk mitigation, and emergency response* includes general disaster preparedness and emergency management, as well as main activities related to establishing the International Health Regulations (2005). Costs include activities required to improve a country`s capacity to minimize the effect of a disaster, and its response to the same, including the creation of Emergency Management teams within Ministries of health, with functional emergency operation centers for coordinated response, laboratory capacity, and national action plans for emergency preparedness. The scale-up of laboratories is linked to the scale-up of health facilities. Furthermore, all countries in our sample require strengthening of their IHR core capacities, moving towards "sustainable capacity" in	The ambitious scenario sets out a series of activities and targets all countries should meet to best minimize the effects of disasters, best handle and coordinate response to emergencies, and to establish compliance with the International Health Regulations (2005). It also considers the rebuilding or repairing 100% of conflict affected health facilities and its related costs, as well as the costs primarily related to health worker time in disaster-relief situations.	The progress scenario deviates very slightly from the ambitious scenario, with the only difference being where resource needs are linked to other targets, such as new facilities.<br><br>*Risk mitigation and emergency response:* the same level of investments in most components, except a fewer number of labs where fewer health facilities are being built.

[20] World Bank CPIA building human resources rating, a part of the Social Inclusion and Equity CPIA cluster.

[21] While revenue-raising is an important part of health financing, it has negligible incremental costs for this exercise, as its costs should be borne outside the health sector.

each.<br>• *Post conflict reconstruction*. For the reconstruction or repair of health facilities after conflicts, the number of facilities that were either destroyed or severely damaged that are counted within the baseline of health facilities, and have not been fixed or rebuilt, are modelled as being fixed and rebuilt over three years after a period of stability in post-conflict settings. This applied to the Conflict and Vulnerable category countries, where data was available.<br>• *Post-emergency relief* entails the additional workforce costs in countries currently recovering from emergencies or conflicts, as well as countries currently in conflict where our model assumes a peace agreement will hold. It is mainly comprised of a hazard pay given to health workers, the number of which are estimated in proportion to the share of the population affected by conflict, or by a maximum quarter of workers who will be delivering services in such circumstances. This applies to all Conflict and Vulnerable category countries.<br>• It is assumed that national contingency funds and the World Bank Group's Pandemic emergency financing facility would constitute the first line of resources available for responding to an emergency. Their costs fall outside the health sector, and are not included here		*Post conflict reconstruction:* same costs as in the ambitious scenario.<br><br>*Post-emergency relief:* same methods as in the ambitious scenario, but with lower costs as the volume of health workers is lower in the progress scenario.
**Health service delivery**:<br><br>We consider four service delivery platforms, representing different modes for providing patients with information, counselling, essential preventive commodities, screening, diagnosis, treatment, and follow-up.<br><br>**Platform 1**: ***Policy and population-wide interventions*** focus on policies and information communication that can be delivered to the population *en masse* at relatively low cost, to support changes in behaviours among risk groups in the population, whether for preventive purposes (i.e., reduce smoking, promote physical exercise, sleep under a mosquito net) or to ensure an appropriate response to a health problem (i.e., ensure that a child with diarrhoea takes oral rehydration salts and increased intake of fluids). Such interventions can be rapidly expanded at low cost even as countries are in the process of building up the foundations of their health systems.<br><br>***Platform 2: Periodic schedulable and outreach services:*** includes services which are provided routinely and periodically. The key characteristic of these interventions is that they are standardized and have low information asymmetry yet require an individual transaction with a qualified health worker. They require contact with health workers, but through brief and schedulable interventions (e.g., immunization, routine antenatal care, iodine supplementation). Because of their relatively high level of standardization, a number of interventions can be delivered through health workers with short term training, meaning that they can therefore be rapidly scaled up even in resource constrained systems.<br><br>***Platform 3: First level clinical services***: includes mainly individualized health-care interventions that are specific to the patient's needs, delivered through primary level health facilities. Examples include treatment of sexually transmitted infections, treatment of TB and treating and	Scale-up curves vary across the four country typologies. Scale-up towards global targets follow the same curves as published estimates where available.<br><br>In general, interventions under platforms 1 and 2 are rapidly scaled up across country groups, although the more advanced groups (HS2, HS3) do have steeper curves and attain benchmarks earlier than 2030, whereas conflict and foundation countries only attain benchmarks for their groups in the final year 2030. Moreover, conflict countries require a certain lag time before the country situation becomes stabilised and service expansion can start.<br><br>Coverage pathways for Platform 3 services varies more substantially between the country groups, as coverage for these services is modelled to increase slowly in initial years in Conflict and Low income countries, but to follow a linear scale-up in HS2 and HS3 countries.	The set of services is the same in both scenarios. The differences between the two lies mainly in the rate at which systems are expected to transform and expand.<br><br>For platforms 1 and 2, extensive progress towards universal coverage is modelled for all country settings. The model maintains fairly rapid scale-up curves for interventions delivered as part of these two platforms, and we estimate that they will attain close to the same levels of coverage by 2030 as in the ambitious scenario. Therefore, we close the coverage gap between current standards and the ambitious scenario "target" coverage by 90%.<br><br>For platforms 3 and 4 however the gap

managing non-communicable diseases such as diabetes. Typically these services require interaction with a health worker that has a certain level of skills and access to diagnostic tools, therefore requiring the foundations of health systems to be built up before services can be expanded.<br><br>***Platform 4: Specialized care***: would typically be delivered by highly skilled health personnel, and rely on specialized diagnostic and referral systems. Examples of interventions include diagnosis and treatment of cancer, management of obstructed labor, and management of severe malnutrition. Services in this category are typically heterogenous, highly individualized and transaction intensive. They have a high asymmetry of information between users and providers and require high quality service provision.	Coverage pathways for platform 4 are the most diverse across country groups, and this is also where end targets for 2030 diverge the most, since Conflict and Vulnerable countries are expected to not be able to expand these services until significant investments have been made to strengthen the foundations of their health systems, and therefore these countries do not attain the 95% targets for a set of services.<br><br>Supplementary material provides more detail on intervention targets.	between current standards and the ambitious scenario coverage target is closed by two-thirds (67%) within the progress scenario. This is not to say that countries should adopt lower targets, but that within our model, more time would be required for countries to attain the targets.
**Equity considerations:**<br>Our model considers equity at multiple levels.<br><br>First, many health system interventions will support efforts to leave nobody behind through investments in governance and health information systems.<br><br>Secondly, our infrastructure and health workforce models are structured to represent reorientation of care models towards close-to-client primary health care, with explicit consideration of rural vs urban needs.<br><br>Third, on the health services side, we model a rapid expansion of "pro-poor" interventions and strategies such as bed nets and neglected tropical disease interventions through population wide and the periodic schedulable and outreach strategies, and scale these up to high coverage targets.<br><br>Fourth, we incorporate costs for specific health activities for marginalised populations such as alcoholics, drug users, transgender populations and prisoners; and making health services adolescent -friendly.<br><br>Fifth, we model interventions to address social determinants of health, including cash transfers, and improving environmental conditions (water and sanitation, clean cooking fuels).	Example: governance includes costs for extending population participation.<br><br>Example: health workforce densities are higher in rural areas.<br><br>Example: bed nets are rapidly scaled up in all countries.<br><br>Example: outreach services for injecting drug users.<br><br>Example: conditional cash transfers for expecting mothers to support facility-based delivery; general cash transfers to promote families to seek preventive health care and for children to continue schooling.	The progress scenario includes similar investments in Governance.<br><br>The progress scenario maintains a focus on rural health workers and a higher proportion of the gap is closed for health centers than for hospitals.<br><br>Progress scenario maintains high investments in pro-poor strategies.<br><br>Progress scenario maintains high investments in strategies targeting vulnerable groups.<br><br>Conditional cash transfers for skilled delivery are included in the Progress scenario.

2.4 Scale-up curves

Table S4 highlighted typical service characteristics which identify which specific constraints related to the organization of service provision are present. In low and middle income countries where health systems face problems of inadequate human resources, services with high transaction intensity will need to be expanded at a more limited pace. Our model designs a pathway for each country through which health systems are

strengthened and service coverage is expanded. Given that our model uses year-specific projections for the number of people reached with each intervention, we designed stylized scale-up curves to model the potential expansion of service coverage within each platform and across different settings, exemplified through the four country groups.

In theory, the scale up of service provision can follow a wide range of curves. Figure S3 presents a variety of curves used within our model.

Figure S3. Types of health service scale-up curves used in the modelling

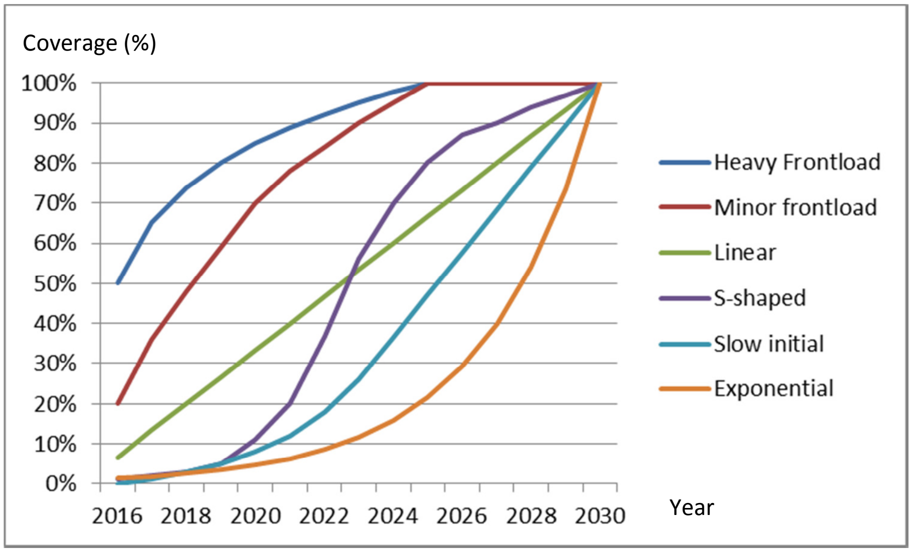

Each intervention is linked to one of three families of scale-up curves "s". The first two platforms (policy and population wide interventions and periodic outreach services) are both associated with scale-up curve type "s1" which indicates that relatively rapid scale-up is possible.[22]

The expansion of facility based care is associated with scale-up curve type "s2" which is less frontloaded than "s1" because service expansion relies on already having a strengthened health system with functioning and accessible facilities that are adequately staffed and able to provide quality outpatient care. The scale-up curves of health services for this platform therefore closely follow those of health system infrastructure and health workforce.

Finally, specialized care interventions require relatively more specialized resources, both in terms of skilled health workers and specialized equipment and facilities, which take longer to acquire and will rely more heavily on investments in the health system, and as such are scaled-up within our model according to curve type "s3".

Figure S4 shows typical stylized scale-up curves for selected interventions from the four service delivery platforms in a stylized Health System 1 country. Each intervention is assigned to a delivery platform which is associated with a specific shape of the scale-up curve, differentiated for the four country groups. For certain interventions, we do not apply the stylized scale-up curves but instead use globally projected targets and scale

[22] There are two exceptions within this group however: one is policy and regulation interventions, for which another set of curves are applied to take into account institutional build-up –see below; and the second exception are the interventions primarily funded outside of the health sector (water, sanitation and hygiene, and clean cooking stoves which both require significant hardware investments and are therefore also scaled up using curve type s2).

up curves(such as for Rotavirus vaccine, in the example below, where we use GAVI projected targets and scale up curves).[23] Baseline coverage levels are country-specific.

Figure S4. Examples of platform- and intervention-specific scale-up curves (stylized example for a HS1 country)

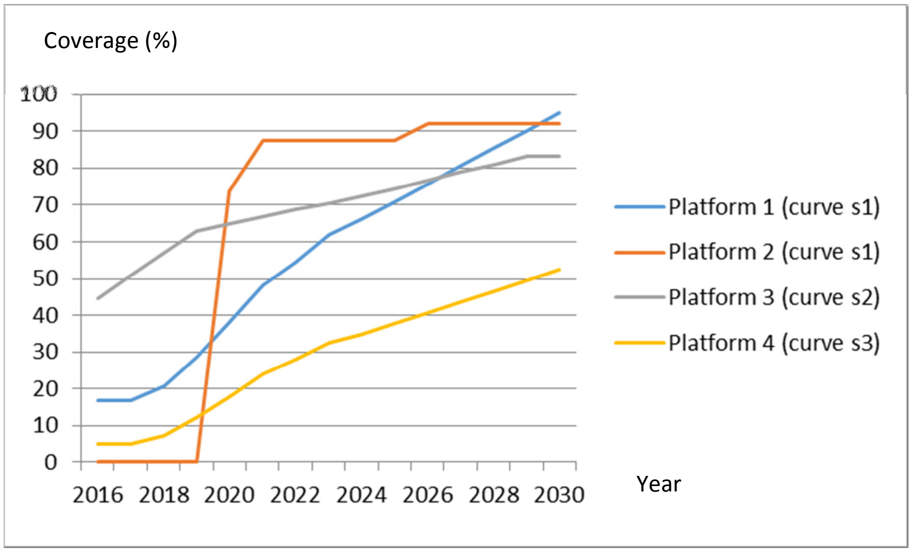

Note: Figure S4 presents data for one specific country, for the following interventions:
Platform 1: Policy and population-wide (curve s1): Hand washing with soap
Platform 2 Periodic schedulable and outreach services (curve s1): Rotavirus vaccine
Platform 3 First level clinical (curve s2): Malaria treatment in adults
Platform 4 Specialized care (curve s3): Screening for risk of cardiovascular disease/diabetes.
The Figure presents a stylized example only, for the Ambitious scenario. Every country and intervention has a unique starting point and end point to which curves are applied.

Moreover, the scale-up curves vary across the five country typologies. As Figure S5 shows, scale-up curves are modelled to take on different shapes depending on the country context. It should be noted, however, that the least difference between groups within our model is for services delivered in platforms 1 and 2, where even in the most fragile contexts we can still expect rapid progress towards universal health coverage for these types of interventions and services, should resources be made available.

[23] GAVI, the Vaccine Alliance. 2015 Strategic demand forecast.

Figure S5. Examples of scale-up curves from start to end point for services type “s2” (stylized examples by country group)

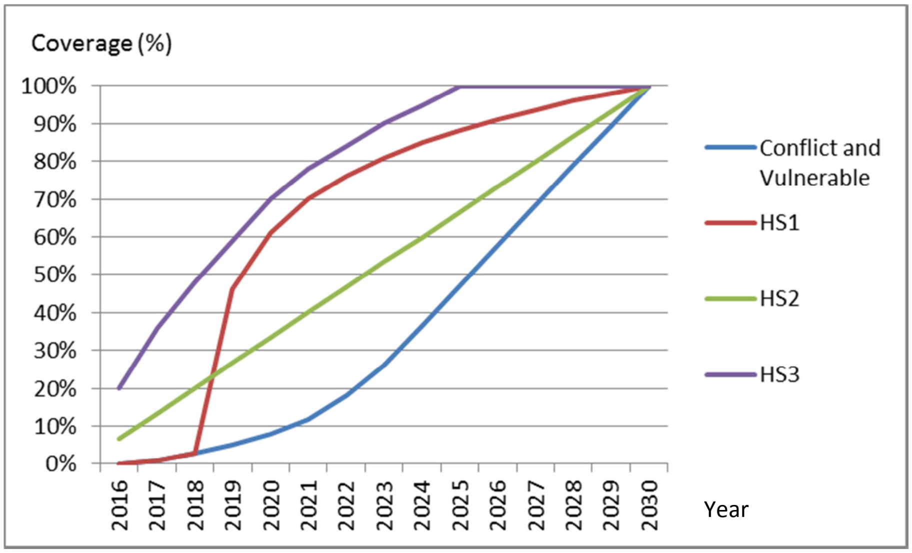

Table S7 outlines the stylized curves for service types s1, s2, and s3, and presents the generic SDG 2030 targets applied within our model for the ambitious scenario.

Table S7: Stylized curves and assumptions for target coverage achieved by 2030, Ambitious scenario, by country group

Country group	Curve type (s1, s2, s3) and Generic 2030 target for the Ambitious scenario (in parentheses)		
	**s1 (platforms 1 and 2)**	**s2 (platforms 1 and 3)**	**s3 (platform 4)**
**Conflict**	Minor frontload from 2017 or 2019<br><br>(in general reach 90% coverage by 2030)	Slow initial<br><br>(in general countries reach 80% coverage by 2030)	Exponential<br><br>(in general countries reach 80% coverage by 2030)
**Vulnerable systems**	Minor frontload from 2016<br><br>(in general reach 90% coverage by 2030)	Slow initial<br><br>(in general countries reach 90% coverage by 2030)	Exponential<br><br>(in general countries reach 80% coverage by 2030)
**Health System 1**	Minor frontload from 2016<br><br>(in general reach 95% coverage by 2030)	Major frontload from 2019<br><br>(in general countries reach 95% coverage by 2030)	Slow initial<br><br>(in general countries reach 95% coverage by 2030)
**Health System 2**	Heavy frontload<br><br>(in general reach 95% coverage by 2028)	Linear<br>(in general countries reach 95% coverage by 2028)	S-shaped adjusted<br><br>(in general countries reach 95% coverage by 2030)
**Health System 3**	Heavy frontload<br><br>(in general reach 95% coverage by 2025)	Minor frontload<br><br>(in general countries reach 95% coverage by 2025)	Linear<br><br>(in general countries reach 95% coverage by 2030)

With respect to policy interventions targeted at reducing non communicable disease (such as policies to restrict the use of tobacco), the model works in a step-wise fashion. The policy is either considered to be in place or not. Countries are first classified according to their current policy status, which is assumed to correspond to a certain level of investment. They are then modelled to shift towards full policy implementation in a given year depending on their country group.

The coverage curves for the Ambitious scenario were designed with the ambitious SDG agenda in mind, including targets for universal coverage of essential health interventions and their associated health impact.

Within the Progress scenario, we assume somewhat lower targets across systems as well as services:

- First of all, we take as our starting point that even in the more limited progress scenario, extensive progress can be made towards universal coverage in all settings when it comes to population-wide and outreach services. We therefore keep the rapid scale-up curves for interventions delivered as part of platforms 1 and 2, and we estimate that they will attain close to the same levels of coverage by 2030 as in the ambitious scenario. Therefore, we close the coverage gap between current standards and the ambitious scenario "target" coverage by 90% for interventions in packages 1 and 2. [24]
- With respect to platforms 3 and 4, the gap between current standards and the ambitious scenario coverage target is closed by two-thirds (67%) within the progress scenario. This is not to say that countries should adopt lower targets, but that within our model, universal targets are not reached by 2030, and countries will require more time for these to be reached.
- The 2030 targets set for coverage within the Progress scenario are thus intervention-specific and country-specific, depending on the current country baseline and the distance to the global benchmark.
- Health systems investments in the Progress scenario mirror (and drive) the assumptions in scale up of service delivery assumptions. For the health workforce component, a scale-up of "Other" health workers to close the gap by 90% is modelled, to increase the number of community health workers and health workers who provide outreach services as part of platforms 1 and 2, whereas the gap for the required numbers of nurses, midwifes and doctors is only closed by two-thirds, given that this is the qualified workforce needed to provide services in platforms 3 and 4, where the Progress scenario models a convergence towards gaps being closed by two-thirds. Similarly, the infrastructure model assumes that the difference between the currently existing infrastructure and the density benchmark is only reduced by two-thirds for hospitals, while 90% of the gap is closed for health centers..

The purpose of presenting two scenarios is to present different resource implications, to stimulate debate at the global and country level regarding what strategies can be implemented and what targets should be set for 2030, and to highlight the usefulness of scenario generation to compare and discuss alternative investment profiles. All countries can achieve universality progressively, and the conditions for doing so will vary across settings.

As shown in Table S7, our model aligns the targets used within the ambitious scenario with previously published global strategies and benchmarks. For areas where there were no published strategies with specific targets, we applied the 2030 targets outlined in table S7, by country group, which are referred to as the "generic SDG targets" applied within our model.

[24] Policy interventions attain the same full implementation level in the Progress scenario but in a later year than in the Ambitious scenario.

Table S8: Type of coverage target applied within model to be achieved by 2030, by scenario and country group

Programme area	*Ambitious scenario2030 targets*	*Progress scenario 2030 targets*
RMNCH, Nutrition, WASH	Generic SDG targets (80, 90, 95% by service type and country group)	Platforms 1 and 2: close 90% of the gap to reach the targets * <br>Platforms 3 and 4: close two-thirds of the gap to reach the targets *
Child immunization	GAVI forecasts	Closes two-thirds of the gap to GAVI forecast targets*[25]
HIV/AIDS	UNAIDS Fast-track	Closes two-thirds of the gap to reach the Fast-track targets*[26]
Malaria	WHO Global Malaria Strategy	Closes two-thirds of the gap to reach targets outlined in the strategy.
TB	Applies targets from the Stop TB Partnership Global Plan to End TB 2016-2020 for 64 countries. <br>Applies targets from Menzies (2016) for 3 countries.[27]	Applies targets from the Stop TB Partnership Global Plan to End TB 2016-2020 for all countries.
NCD policy interventions	Implement policies in all countries, with schedule depending on the group classification	Implement most policies in all countries, with a later schedule for implementation, depending on the group classification <br>Certain policies are only implemented in HS2 and HS3 countries, such as the brief interventions for tobacco, alcohol and physical inactivity. Policies related to diet and salt intake are not scaled up in conflict and vulnerable countries.
NCD screening and treatment	Reduce unmet need by half.	Closes two-thirds of the gap to reach the targets in the ambitious scenario.
Neglected tropical disease	WHO Investment case on neglected tropical diseases.[28]	Closes two-thirds of the gap to reach the targets outlined in the NTD investment case* (except for Preventive chemotherapy (PC) including post-PC surveillance, where the same targets are attained as in the ambitious scenario)

*Gap refers to the 2030 target minus the country-specific baseline data point (2015)

[25] Portnoy et al, 2015.
[26] Stover et al, 2016.
[27] Menzies et al. (2016).
[28] WHO (2015), Investing to overcome the global impact of neglected tropical diseases.

Section 3: Start and end points within models

This section presents a summary of the sources of data for start points and 2030 targets set within our model.

Table S9: Sources for assumptions on baseline and 2030 targets within the model

**Area of analysis**	**Source for assumptions and baseline data**	***2030 targets: ambitious scenario***	***2030 targets: Progress scenario***
Infrastructure			
Number of facilities	Global Health Observatory data (1) and country-specific country planning and health system documents, complemented by information from country representatives attending the country review meeting.	Benchmarks for the minimum number of facilities required to deliver care to the population uses the same targets as applied by WHO for HLTF 2009 (2), further adjusted to allow for people-centred primary health care. The benchmarks are attained only for HS1, HS2 and HS3 countries, while these are nearly estimated as being reached for the C and V groups.<br><br>The benchmarks are one urban health center per 12,000 people, one rural health center per 6,000 people, one urban district hospital per 100,000 people, one rural district hospital per 50,000 people, and one provincial hospital per 1 million people.	Closes two-thirds of the gap closed in the ambitious scenario, except for health centers, where 90% of the gap is closed.
Safe Hospitals	Costs for facilities to meet safe hospital standards are based on the WHO Comprehensive Safe Hospital Framework and country specific studies. Estimates on the baseline numbers for the share of each type of facilities that require retrofitting to fit safe hospital standard was provided by expert opinion, across country types.	All new facilities are equipped to meet safe fospital standards. The retrofitting of existing facilities is determined by the number of facilities that have urgency to withstand hazards (thus maintaining functionality in emergencies and disasters), as measured by Natural Disaster Propensity24[29]. Both of these are estimated to be a fixed % of new building costs.	Same methodology as in the ambitious scenario, but with fewer new facilities being built.
Health workforce			
Number of workers	Global Health Observatory data (1), complemented by information from country representatives attending a country review meeting.	Target human resource densities, and mix of cadres, estimated as part of work for the Global Strategy for Human Resources for Health, Adjusted "other" workers based on rural population distribution, to reflect different country local needs.<br><br>While final ratios vary across	Closes two-thirds of the gap in the ambitious scenario, except for "other" workers, where 90% of the gap is closed.

[29] Component of the Index for Risk Management – INFORM, http://www.inform-index.org/. Accessed March 2016.

		countries and settings within our model, as a general rule, the targets set are for 4.45 doctors, nurses and midwives and 2.15 "other" [30] health workers per 1000 people, with an additional 2 "other" workers per 1000 rural population.<br><br>The benchmarks are attained only for HS1, HS2 and HS3 countries, while these are estimated as being nearly reached for the C and V groups.	
Supply chain			
Fixed and Recurrent costs	Costs are based on the additional volume and value in the supply chain as a result of additional interventions or higher intervention coverage of the model. There is no assessment of current volume of commodities passing through the system.	The total volume and value of commodities related to the interventions modelled as being delivered determines costs of running the supply chain, and the required infrastructure and equipment (warehouses, trucks) to handle these commodities. Estimates are derived using an updated version of the JSI / USAID deliver model.[31]	Follows the same approach as the ambitious scenario, and here estimates are based on the relatively smaller volume of commodities related to the lower levels of coverage of interventions.
Cold chain	Existing cold chain equipment and volume capacity shared by WHO/IVB (Gavi grant proposals, cMYP and EVM results).<br><br>Baseline vaccine coverage and projections derived from GAVI projections.	Target cold chain capacity determined by projections of the increased volume of immunization commodities, which in turn is determined by coverage levels of immunization interventions.	The projected expansion of cold chain capacity is lower, since it aligns with the lower coverage targets for vaccines in this scenario.
Health information systems			

[30] "Others" refers to the other cadres of health workers in the WHO Global Health Workforce Statistics database, which include, dentists, pharmacists, laboratory health workers, community and traditional health workers, and health management and support health workers.
[31] For more information, see: http://deliver.jsi.com/dlvr_content/resources/allpubs/policypapers/EstiCostGlobSuppMDG.pdf

Health Facility Information Systems	Specialized staff, such as demographers and statisticians, is modelled to cover a certain population catchment, which corresponds to that of district hospitals. It is then assumed that the current amount of this staff is parallel to the existing number of district level hospitals in country. Where we assume a need to refurbish hospitals to meet Safe Hospital standards, we also assume that these are understaffed and do not have health information system staff.	We estimate the resource needs to strengthen the health information system at the facility level. This is dominated by specialized human resources not considered in the health workforce component above, that contribute specifically to health information and surveillance system. Targets here follow the targets and scale up of new district hospitals, and the refurbishment of them, where district hospitals that are assumed to need refurbishment also are assumed to not already have this specialized staff.	The progress scenario follows the same methodology as the ambitious one, but reflects the lower number of district hospitals modelled as being built or refurbished.
Financial Information System (FIS)	Costs are estimated for strengthening the system of tracking financial resources in the health system. Expert opinion from the HIS department has identified current level of FIS development for our 67 countries.	Specialized staff, activities and meetings are costed which are modelled as improving the level of FIS development. Countries move up levels until reaching the top level of a mature financial information system.	The progress scenario follows the same methodology, except for assuming slower improvements and movements up along the different levels of FIS maturity.
Surveys	Costs are estimated for the needs for 3 periodic surveys beyond censuses a country is already expected to carry out. The number of each type of survey needed is proportional to population.	A full schedule of surveys is carried out in all countries, estimating resources for household visits and interview costs.	A subset of countries with considered difficulty to carry out surveys are estimated as only being able to carry out half as many surveys until 2020, where they are estimated as being able to carry out the full schedule of them.
Health Workforce Information System	Costs are estimated at creating and managing a health workforce information system.	Specialized human resources comprise the majority of costs estimated, where a portion of the target is estimated as one data clerk per 500,000 population, while a team of technical professionals of different sizes is estimated as needed across 5 different population brackets. Scale up follows a curve where all countries reach 80% of targets by 2024.	Countries are split as in the survey component, where a subset of countries is estimated as only being able to scale up to 2/3 of targets by 2020, and then move towards final targets in the last 10 years.
National Statistical Office, Public Health Institute and Governance	Costs are estimated at creating and staffing the governance structure to manage the health information system of a country, including a national statistical office, a national public health institute, and a health information department in the ministry of health.	Capital investments, such as equipment, and recurrent costs, including specific specialized human resources, are scaled up following the same assumptions as in the above component.	The progress scenario varies from the ambitious one in the same manner as in the health workforce information system component.
Governance			
	Costs are assessed for activities to improve a country`s level of governance. To measure a country`s level of governance, we employ the Country	We estimate costs for activities that will bring each country towards a CPIA score of 6.	Within the progress scenario, countries currently scoring less than 5 are brought

	Policy and Institutional Assessment (CPIA; World Bank, 2007). Countries are scored in the range 1-6, where 6 equates to optimal performance.	The type and intensity of activities is determined by expert opinion and draws upon the same methodology used as in HLTF 2009(2). Each country is modelled as improving its CPIA score after following different regulatory and planning activities for three years, until reaching a CPIA score of 6.	towards a CPIA score of 5.
Health Financing Policy			
Strengthening the purchasing function, through new health finance reform	Contact and surveys with WHO regional and country offices identified countries that have just embarked on health finance reform or are likely to do so in the near future. Countries already undergoing reform were not evaluated in terms of current progress, and assumed to be able to continue reform as a continuation of current resources devoted to these processes.	Costs are estimates as a percentage of GGHE, the source for which are projections carried out based on global growth and fiscal projection scenarios (see below for more details.)<br><br>The source for the percentage share of general government health expenditures drew upon the work done for Social Health Insurance administrative costs as part of the 2009 High Level Task force on Innovative Finance, as well as country level National Health Accounts data, identifying administrative costs at between 1 and 2% of GGHE.	Similar approach as above, in that within the progress scenario, only that the more moderate financing scenario, and resulting smaller GGHE values, was used.
Emergency Risk Management			
Post-Conflict Reconstruction	The numbers of facilities destroyed or severely damaged in conflict were identified from a variety of WHO and external sources, including official multiyear reconstruction plans. Several plans had associated cost projections which provide data on resources needed to rebuild or repair severely damaged facilities. We applied the ratio of these costs to the cost of constructing new facilities in the same country, to allow for the estimation of repair and reconstruction for countries for which no cost data was obtained.	All severely damaged or destroyed facilities are repaired or rebuilt.	Same as in the ambitious scenario.
Emergency Relief	Costs for emergency relief in pre-existing humanitarian or conflict settings was estimated as being captured by a calculation of hazard pay for health workers not currently in place. Information on populations affected by conflict were identified from WHO Humanitarian Response plans. No estimates or considerations of current amounts of emergency relief were considered for countries not currently in	A country-specific proportion of modelled number of health workers receive additional hazard pay for the estimated duration of emergency relief, estimated for post-conflict contexts as being equivalent to the share of a country`s population affected by conflict, and as being 25% of the modelled workforce for non-conflict humanitarian scenarios, such as countries recovering from	Same as in the ambitious scenario, but reflecting the lower density of health workers modelled in this scenario.

	conflict.	Ebola.	
Laboratory Services	Based on published literature for primary data of existing laboratories at district, provincial and national level[32] and extrapolated baseline values.	Based on the minimum number of laboratories required per capita as identified by the Georgetown University Laboratory Capacity Costing Estimates Tool based on WHO and CDC Technical Guidelines for Integrated Disease Surveillance and Response in the African Region, 2010.<br><br>The benchmarks are attained for all countries by 2030 within our model, and are considered to be one district level lab per 150,000 people, one provincial level lab per 1,500,000 people and one national reference lab per country.	Closes two-thirds of the gap identified in the ambitious scenario.
Emergency Preparedness and Response, and International Health Regulations (2005)	Country self-assessments of compliance with core capacities of the International Health Regulations were used to identify the current starting point of countries towards meeting full achievement of these core capacities. For non-IHR components, used national planning documents and WHO Health Emergencies program working documents and expert opinion.	Activities carried out to raise the scores of the indicators within the core capacities of the International Health Regulations. For emergency preparedness and response components considered separately from the IHRs, the WHO Health Emergencies program provided guidance on each of these (ie. The size and equipment profile of a national poision control center).	The methodology is the same as in the ambitious scenario, as this component is deemed to be of strategic importance to all countries, and full attainment of targets necessary to ensure minimal effects of cross-border pandemics and epidemics.
Demography, epidemiology and current coverage of health services			
Service coverage modelled within OHT	OHT includes pre-populated country profiles that include demographic and epidemiological data specific to the country.[33]<br><br>Default coverage data within OHT originating from DHS and MICs surveys, and/or expert opinion where surveys not available[34].	See tables S6 and S7 above.	See tables S6 and S7 above.
Service coverage modelled in Excel	Baseline coverage adopted from existing documents (see table S7 above.)	See tables S6 and S7 above.	See tables S6 and S7 above.

Notes to table:

(1) Global Health Observatory http://www.who.int/gho/en/

(2) Constraints to Scaling Up the Health Millennium Development Goals: Costing and Financial Gap Analysis. Background Document for the Taskforce on Innovative International Financing for Health Systems. Working Group 1: Constraints to Scaling Up and Costs http://who.int/choice/publications/d_ScalingUp_MDGs_WHO_finalreport.pdf

[32] Elbireer et al., *The Good, the bad and the unknown : quality of clinical laboratories in Kampala, Uganda*. PLos One, 2013 May 30 ; 8(5), Schroeder, Lee F. and Amukele, Timothy. *Medical Laboratories in Sub-Saharan Africa That Meet International Quality Standards.* American Society for Clinical Pathology, 2014; 141 : 791-795, Scott et. al, *Establishing a simple and sustainable quality assurance program and clinical chemistry services in Eritrea.* Clin Chem, 2007, Nov ; 53(11) : 1945-53, and communication from the Namibian Institute of Pathology, and the Ministry of Health of Bhutan.

[33] For details, see www.avenirhealth.org/software-onehealth.php

[34] For immunizations, the Gavi Strategic Demand Forecast 2015 was used.

Section 4: Cost and impact projection methods

This section describes the methods and tools used to estimate the resources required and the potential impact of expanding health intervention coverage towards the SDG targets and UHC.

4.1 Country-specific projections

The analysis considers the unique context of every country when modelling investment needs. The country-specific cost and impact outputs take into account the demographic and epidemiological context of individual countries, including projected urbanization,[35] as well as the current health system structure, and country-specific prices for inputs. Modelling is set up to model standards of performance, grounded in empirical data, where possible (Table S8 above).

4.2 Defining health sector costs vs costs in other sectors ("below and above the line")

In this paper we focus the discussion on the resource needs required in the health sector. However, within our analysis we have also examined costs that would fall outside health sector expenditure but that have some impact upon health. We employ terminology traditionally used within health accounts, that of "above the line" for those costs that we classify as health sector spending, and "below the line" for costs that would not be funded through health expenditure. Below the line costs were estimated for clean cook stoves, cash transfers for poor populations, pre-service education of health workers, and the hardware investments required for water, sanitation and hygiene (WASH).

4.3 Ingredients-based bottom-up costing

The general approach is an ingredients-based costing (Quantities x Prices). Within each area, we specify the inputs required to carry out activities in order to attain the benchmarks. Inputs are defined relative to total population, population density, or to other appropriate denominators such as number of districts or the projected number of health facilities per country and year. Prices are country-specific, where possible (see below).

The non-use of unit costs implies that economies of scale (in terms of decreasing and/or increasing unit costs) is not taken into account. Instead, we consider that in certain settings, such as more sparely populated rural settings, there may be a need for more fixed resources for smaller populations than in urban settings, and as such, the implicit cost per capita is higher in most rural settings than in urban ones. A typical example is the health workforce and infrastructure modelling, where our model assumes a need for higher density of infrastructure and health workers in rural areas than in urban areas.

For service delivery costs, each intervention is associated with specific inputs and prices. Cost projections are needs-based, taking into account country-specific epidemiology and coverage trajectories. This differs significantly from an approach which would project an increase in average per capita utilization visits and associated costs. A needs-based approach allows us to identify which interventions drive the costs, and to model the impact of preventive interventions on the need for curative care.

4.4 Tools

Our estimates draw upon pre-existing models and estimations, including global strategies and plans in each respective area. Several health system models draw upon the methods used for the HLTF (2009), applying updates of the same tools (e.g., Governance, Infrastructure) or using Excel spreadsheets designed for other recent assessments (i.e., WHO's Global strategy on human resources for health: workforce 2030). There is an explicit effort, to the extent possible, to be consistent with other estimates on resource needs for the 2016-2030 period where those costs have already been made public, and to make use of the same estimates and projection

[35] United Nations Population Division, World Urbanization Prospects the 2014 revision. https://esa.un.org/unpd/wup/

models, but making sure to take out costs for shared resources such as health worker time, to avoid double counting.

Our analysis thus makes use of established tools and methods, many of which have been peer reviewed and published. Most of the health service scale-up and related impact is modelled within the OneHealth Tool (OHT) version 5.47, a software product whose development is overseen by the UN Inter Agency Working Group on costing (IAWG-COSTING), and carried out by Avenir Health[36]. OHT includes pre-populated country profiles that include demographic and epidemiological data specific to the country. The tool is also pre-populated with cost assumptions around consumables, and the health workforce inputs required, per service provided. Table S5 above indicates which interventions are modelled within OHT.

OHT is developed within Spectrum which is a suite of models that aim to provide policymakers with analytical tools to support priority setting and decision making processes. As such, OHT incorporates a variety of impact estimation models – including the Lives Saved (LiST) tool, the FamPlan model, and a number of models for Non-Communicable Diseases, – in order to project the costs and health impacts of scaling up specific interventions and activities in a given country.

Health impact is estimated through the OHT impact models that are directly linked to year- and country-specific intervention targets. Box S2 provides additional detail on the OHT models used for the analysis, as well as additional sources of data for projected impact. The key added value from projecting service coverage within the OHT is the linkage of separate disease impact projection models through a central demographic model, which ensures that deaths averted are not "double counted" but also allows us to benefit from the interaction of the interventions on different indicators in the tool (an example being a change in fertility rates from family planning affecting the number of children in need of a measles vaccination).

Box S2. Demographic and epidemiological models included within the OneHealth Tool and used for the analysis

For the majority of the health impact projections, we used the impact projection models built into the OneHealth Tool.

The *DemProj* model includes a demographic profile for every country, based on data produced by the Population Division of the United Nations. DemProj projects the population over time by age and sex, based on assumptions about fertility, mortality, and migration. The population projections within DemProj are used by the other modules to support calculations on the population in need for each intervention, the associated cost and health impact.

The *Lives saved Tool* (LiST) estimates the effect on maternal and child mortality and morbidity of scaling up a range of child and maternal health interventions, including malaria interventions. The model has been developed under the guidance of the Child Health Epidemiology Reference Group (CHERG). The LiST model uses the user's inputs on projected changes in the coverage of health interventions to adjust mortality rates and cause of death structure over time. The outputs produced include changes in population level of risk factors (such as wasting or stunting rates) and cause-specific mortality (including neonatal, children aged 1–59 months, maternal mortality, and stillbirths). The association between an input (change in intervention coverage) with one or more outputs is driven by intervention-specific effectiveness for reduction of the probability of that outcome (mortality of risk factor).

The *AIDS Impact Model (AIM*) uses historic data combined with user-inputted coverage targets to project the consequences of the AIDS epidemic including: the number of people infected with HIV, AIDS deaths, and the

[36] http://who.int/choice/onehealthtool/en/

number of people needing treatment. The projections are based on UNAIDS estimates and projections of adult prevalence, which is combined with information on the age and sex distribution of prevalence and progression to death in order to estimate the number of new adult infections by age and sex. Estimates of new infant infections are based on HIV prevalence among pregnant women and the rate of mother-to-child transmission, which is dependent on infant feeding practices and the coverage of prophylaxis with antiretrovirals (ARVs). New infections progress over time to a symptomatic stage where antiretroviral treatment (ART) is required. Those who receive first-line and or second-line ART will have extended survival. People at any stage are subject to non-AIDS mortality at the same rates as those who are not infected.

FamPlan uses targets set for family planning to model projected changes in fertility. The user can enter future contraceptive prevalence goals along with assumptions about the proximate determinants of fertility and the characteristics of the family planning program (method mix, source mix, discontinuation rates). The model estimates the number of users and acceptors of different methods by source, the number of pregnancies which are likely to terminate in spontaneous and induced abortions, and the overall fertility outcomes.

The *NCD impact model* estimates prevalence, and incidence of NCDs including cardiovascular disease, lung health, diabetes, and mental, neurological, and substance abuse disorders. The population in the base year is allocated to health states based on initial prevalence. Users can specify scale-up of preventive interventions which reduce incidence, and curative interventions, which reduce case fatality rates. A module within the NCD impact module calculates the prevalence of risk factors for NCDs such as tobacco use, which additionally influences the prevalence and incidence of the diseases. Policy interventions can be implemented at differing levels of intensity which affect the prevalence of risk factors for NCDs, and ultimately the prevalence of NCDs.

With respect to health interventions modelled outside the OneHealth Tool, we drew upon previous estimates where available to compute the additional health impact that would be attained.

Tuberculosis:

TB-related health impact estimates draw upon the Stop TB Partnership Global Plan to End TB 2016-2020.

Cancer:

Impact projections for Cervical, Colorectal and Breast cancer were estimated in Excel using country-specific incidence projections to 2030 (Globocan, 2016). Impact (deaths averted) for ambitious, progress and baseline scenarios was calculated using the following equation:

$$Deaths\ Averted_i = Incidence_i * Total\ Deaths\ Averted_i * Coverage_i$$

Where i is the cancer-stage (I-IV), $Incidence$ is the annual total incidence, $Total\ Deaths\ Averted$ is the proportion of deaths that would be averted at 100% coverage and $Coverage$ is the projected annual coverage rate.

For cervical cancer screening, impact (deaths averted) for ambitious, progress and baseline scenarios was calculated using the following equation:

$$Deaths\ Averted_i = Females\ 30 - 49_i * Positivity\ Rate * Total\ Deaths\ Averted_i * Coverage_i$$

Where i is the cancer-stage (I-IV), $Females\ 30 - 49$ is the total female population between ages 30 & 49 (UN World Population Prospects) , $Positivity\ Rate$ is the proportion of positive cases identified by screening, $Total\ Deaths\ Averted$ is the proportion of deaths that would be averted at 100% coverage and $Coverage$ is the projected annual coverage rate.

Exclusion

We were unable to include impact for some health interventions due to a lack of available models. Table

S5indicates the list of interventions for which we have not modelled gains in mortality and morbidity.

References for Box S2.

Demproj:

USAID. (2008) DemProj - A Computer Program for Making Population Projections.

LiST:

Garnett GP, Cousens S, Hallett TB, Steketee, R, Walker N. Mathematical models in the evaluation of health programmes. Lancet 2011; 378: 515–25.

AIM:

Stover J. (2007) AIM: a computer program for making HIV/AIDS projections and examining the social and economic impact of AIDS. Glastonbury, CT: Futures Institute.

Stover J, Johnson P, Zaba B, et al (2008). The Spectrum projection package: improvements in estimating mortality, ART needs, PMTCT impact and uncertainty bounds. Sex Trans Infect; 84: i24-i30.

FamPlan:

USAID. FamPlan - A Computer Program for Projecting Family Planning Requirements. http://www.healthpolicyinitiative.com/Publications/Documents/1256_1_FampmanE.pdf

The above listed references are available together with other reference materials at: www.avenirhealth.org.

4.5 Cost projection models

Commodity costs were generated by the OneHealth Tool. For interventions not included in the OHT Excel spreadsheets were used. Costs are country- and year-specific. We incorporated a 10% mark-up for wastage. Costs for activities to support programme administration and scale-up were estimated for each programme (Maternal and child health, SRHR, immunization, Nutrition, malaria, HIV/AIDS, NCDs, cancers, Mental Health and Substance Use, neglected tropical diseases, and environmental health) using the WHO-CHOICE standardised programme costs (www.who.int/choice) and using a tracer intervention approach for each programme. This entailed taking the current coverage of service provision (for the tracer intervention, by country), estimating the gap to reach universal coverage, and multiplying country-specific programme costs by the coverage gap. These programme administration costs include costs for training health workers, monitoring and evaluation of programme performance, supervision, information campaigns and general programme management. Some areas already had projected programme administration costs (e.g., TB) in which case we used the pre-existing estimates. Programme cost estimates for adolescent health -i.e., improving the quality and accessibility of health services to provide priority health interventions for adolescents- were estimated drawing upon the approach by Deogan et al. (2012). Costs include general programme coordination at national and district level, development and distribution of national standards for Adolescent Friendly Health Services (AFHS), in-service training on AFHS, information and communication activities, and upgrade of infrastructure and equipment to adolescent friendly standards.

We included costs specifically to provide financial incentives to women seeking to deliver at formal health facilities. We used the same methodological approach as was used for the WHO HLTF (2009) analysis. Thus the cost for incentives was calculated based on their provision to the total eligible population, and not just the

incremental proportion of the population that is currently not delivering in facilities. In addition to the cost of cash transfers, costs are also incurred for administering the program, identifying poor women and paying providers for their services. Given the findings from various countries we assumed that 30% additional costs, calculated as a proportion of the cash transfers, would be the absolute minimum for administration costs.

4.6 Prices

Within our model we apply prices sourced from publicly available references and databases. Where possible, prices are differentiated by country. As a general rule, price assumptions for drugs and commodities refer to generic drugs and the lowest (median) price selected in the international market. [37] The WHO-CHOICE database provides country specific prices for both traded and non-traded goods. [38] Where additional prices were needed but were not contained in the list of previously mentioned sources, we also made use of additional data sources for prices such as construction costs, [39] vaccine prices,[40] etc.

Prices are reported in 2014 USD. Prices from the WHO-CHOICE database, which were available in 2010 USD, were inflated to 2014 using country specific inflators. When costs were drawn from other pre-existing publications (such as NTDs), we adjusted the costs to 2014 USD.

As a general rule, price assumptions within our model do not vary with volume nor over time. Thus, for example, there is no inbuilt consideration of volume discounts for drug purchases. Similarly, we have not modelled an increase or decrease in future prices[41] (e.g., salaries might be expected to increase with GDP growth, and prices of certain drugs or medicines may be expected to decrease). The reason for not modelling changes in prices over time is uncertainty. For many current medications it is likely that biosimilars will be forthcoming in the future patent landscape; however, predictions remain uncertain.

4.7 Modelling increases in life expectancy

Summary measures of health such as life expectancy, healthy life expectancy and healthy life years gained provide a general assessment of country progress towards strong primary care and universal health coverage. The OneHealth Tool (OHT) projections, including Spectrum impact modules (AIM, GOALS, LIST, DemProj, FamPlan, NCD), produce estimates on changes to population and deaths by age, taking into account coverage of interventions to prevent or treat various diseases. Estimates on life expectancy were calculated in Excel, drawing upon outputs from Spectrum/OHT, complemented by additional data when required. The Spectrum model tracks the population by single age as people are born, grow older, and die, and produces outputs on modelled deaths by age. We used these outputs to adjust/construct standard life tables[42] to estimate life expectancy at birth, and drawing upon GBD2010 disability weights by region,[43] to calculate the healthy life years gained due to scale up of interventions

We calculated life expectancy for three scenarios: the first is life expectancy at birth in 2015, the base year of our analysis. The second is life expectancy at birth in 2030 based on projecting current intervention implementation forward without additional investment. The third is life expectancy at birth in 2030 projecting the health impacts of increased investments. Comparing the life expectancy at birth under scenario with additional investments to the projected life expectancy at birth in 2030 with a constant coverage scenario, allows

[37] MSH International Drug Price Indicator Guide http://erc.msh.org/mainpage.cfm?file=1.0.htm&module=DMP&language=English
[38] http://www.who.int/choice/cost-effectiveness/inputs/en/
[39] Data entracted from SPON`s construction costs handbooks, Compass International 2016 Construction Costs Yearbook, and IADB Infrastructure project reports.
[40] Portnoy et al (2015), costs of vaccine programs across 94 low-and middle-income countries.
[41] Traztuzumab for treating breast cancer is an exception, where a forecasted drop in its price is taken into account.
[42] Life tables: http://www.who.int/healthinfo/statistics/LT_method.pdf?ua=1&ua=1 WHO methods and data sources for life tables 1990-2015 (Global Health Estimates Technical Paper WHO/HIS/IER/GHE/2016.8)
[43] For Disability weights, see Salomon et al. (2012).

us to estimate the LE gained through the scale-up of the interventions, whilst implicitly taking into account the background projected increase in LE built-into the UN pop projections.

The 2030 projected life expectancy at birth within the scale-up scenarios includes the impact of scaling up care HIV/AIDS, maternal and child health, and a set of non-communicable diseases (cardiovascular disease, diabetes, asthma, COPD), epilepsy, and mental, neurological, and substance abuse disorders, as modelled through the OHT. Additional data available for Cancers, TB and NTDs were available from models with the same underlying methodology which we were able to incorporate into the calculations using an Excel-based calculation approach.[44] We additionally explicitly show the impact of avoiding still births on life expectancy increases. Intrapartum and Antepartum stillbirths are counted differently to avoided deaths following a live birth. A body of literature suggests that sentience begins at 28 weeks gestation, thus we would consider the fetus as a being from this point in time and would therefore include these data in health gain calculations.[45] Although sentience exists, there appears to be consensus that each stillbirth avoided should not be valued the same as neonatal death following live birth.[46] Thus each intrapartum still birth avoided is weighted at 75% and each antepartum stillbirth avoided is weighted at 25% of a neonatal death.

Table S10a: Modelled increase in life expectancy, selected countries, Ambitious scenario

		Life Expectancy increase		Share of LE_B increase due to conditions within model						
	N	LE_S	LE_B	HIV	TB	Malaria	RMNCH	Stillbirth	NCD	MNS
By country group										
Total for country subset	18	3.24	4.91	4%	11%	1%	29%	6%	46%	2%
Conflict (N=2)	2	1.74	3.12	0%	1%	0%	57%	10%	31%	1%
Vulnerable (N=2)	2	5.24	8.37	3%	9%	16%	57%	8%	6%	1%
HSS 1 (N=2)	2	3.89	6.73	2%	16%	1%	50%	14%	16%	1%
HSS 2 (N=6)	6	3.27	5.50	5%	17%	2%	43%	10%	23%	1%
HSS 3 (N=6)	6	1.17	3.83	3%	5%	0%	7%	1%	81%	3%
By income group										
LIC (N=3)	3	4.74	8.02	3%	10%	9%	57%	12%	9%	1%
LMIC (N=10)	10	3.13	5.31	5%	16%	1%	42%	9%	25%	1%
UMIC (N=5)	5	1.13	3.83	3%	4%	0%	7%	1%	83%	3%

** MNS = Mental Health and Substance Use; NCD = Non Communicable Disease; RMNCH= reproductive, maternal, newborn and child health.*

We ran projections for 18 countries representing 60% of the global burden of disease (2010) and 79% of the population of the 67-country set.[47] This analysis is intended as indicative only. We report two life expectancy results: the first compares life expectancy at birth in 2030 in the scale up scenario with life expectancy at birth in 2030 in the flatline scenario. This is a conservative estimate of life expectancy gain, and referred to in table s10 below as LE_S. Alternatively; we compare life expectancy at birth in 2030 in the scale up scenario with life expectancy at birth in 2015, referred to as LE_B in table s10. We report in our main results the full life expectancy gain between 2015 and 2030. Health system investments are required even in the absence of scale up of

[44] De Vlas et al (2016); Stop Tb partnership (2015), Menzies NA et al (2016).

[45] Quereshi, Z U (2015) ; Phillips and Millum, (2015).
[46] Jamison DT, Shahid-Salles SA, Jamison J, et al.(2006)
[47] Bangladesh, Brazil, China, Democratic Republic of the Congo, Egypt, Ethiopia, India, Indonesia, Iran, Iraq, Mali, Mexico, Myanmar, Nigeria, Pakistan, Philippines, Viet Nam, and Yemen.

interventions to support the current status of intervention implementation in a growing population, thus we believe that LE_B is valid as without these health system investments it may not be possible to continue to implement interventions at current scale. The proposed additional health investments would generate more than double the life expectancy gain than is expected in the flat line scenario. Tables S10a and s10b present life expectancy results for the 18 countries.

Table S10b: Modelled increase in life expectancy, selected countries, Progress scenario

		Life Expectancy increase		Share of LE_B increase due to conditions within model						
	N	LE_S	LE_B	HIV	TB	Malaria	RMNCH	Stillbirth	NCD	MNS
By country group										
Total for country subset	18	2.46	4.34	4%	12%	1%	25%	5%	51%	2%
Conflict (N=2)	2	1.36	2.74	0%	1%	0%	54%	8%	34%	2%
Vulnerable (N=2)	2	3.90	7.03	4%	11%	16%	55%	6%	7%	0%
HSS 1 (N=2)	2	2.97	5.81	2%	18%	1%	49%	12%	17%	1%
HSS 2 (N=6)	6	2.53	4.77	5%	17%	2%	40%	8%	28%	1%
HSS 3 (N=6)	6	0.90	3.56	3%	5%	0%	2%	1%	86%	3%
By income group										
LIC (N=3)	3	3.58	6.86	3%	11%	9%	56%	11%	10%	0%
LMIC (N=10)	10	2.42	4.60	4%	17%	1%	39%	8%	30%	1%
UMIC (N=5)	5	0.88	3.58	2%	5%	0%	1%	1%	89%	3%

The quality as well as quantity of health impact is important. In addition to the life expectancy, the number of healthy life years gained due to this set of interventions was estimated in the OHT projection models where possible, and from additional sources for TB and NTD. Increases in healthy life years lived within the Spectrum impact models are calculated based on comparisons between continuation of the status quo, and implementation of interventions to prevent or treat diseases, resulting in more people alive and healthy, and reduced disability of the population. Across the 67 countries, 81 million healthy life years would be gained in 2030, with a total gain of 535 million healthy life years over the course of the SDG period (Table S11). A calculation such as this is crucial for diseases for which treatment focusses on quality of life rather than cure. For example, mental, neurological and substance abuse disorders contribute only 3% of projected life expectancy gain, but 15% of the projected healthy life years gained.

Table S11: Modelled increase in healthy life years by cause, 67 countries

	HIV	TB	Malaria	RMNCH	Stillbirths	NCD	MNS	NTD	Total
**2015**	-	-	-	-	-	-	-	-	-
**2016**	0.2	0.0	0.0	0.2	0.0	0.3	0.5	0.4	1.6
**2017**	0.6	0.2	0.0	0.7	0.1	0.4	1.0	0.8	3.8
**2018**	1.1	0.4	0.1	1.5	0.2	1.2	1.6	1.3	7.2
**2019**	1.7	0.8	0.1	2.5	0.3	1.8	2.2	1.8	11.1
**2020**	2.4	1.6	0.2	3.7	0.5	2.5	2.8	2.2	15.9
**2021**	3.2	2.5	0.3	5.1	0.7	3.3	3.4	2.7	21.3
**2022**	4.0	3.3	0.4	6.7	1.0	4.2	4.1	3.2	27.0
**2023**	4.7	3.7	0.5	8.6	1.4	5.3	4.8	3.7	32.8

**2024**	5.5	3.9	0.6	10.7	1.9	6.4	5.5	4.3	38.9
**2025**	6.3	4.0	0.8	12.9	2.5	7.7	6.3	4.8	45.2
**2026**	7.0	4.5	1.0	15.4	3.1	9.1	7.0	4.9	51.9
**2027**	7.8	4.7	1.2	18.0	3.7	10.6	7.8	4.9	58.7
**2028**	8.6	4.9	1.5	20.7	4.4	12.2	8.6	5.0	65.9
**2029**	9.4	5.0	1.7	23.6	5.2	13.9	9.4	5.1	73.3
**2030**	10.2	5.2	2.0	26.6	6.0	15.7	10.2	5.2	81.1
**Total**	72.7	44.8	10.3	156.8	31.0	94.5	75.3	50.4	535.8

It is important to note three reasons why our life expectancy impact numbers are underestimates:

Firstly, the increase in LE_S only captures those interventions modelled in the OHT Spectrum platform.

Secondly, the estimated increase in LE_B reflects the built-in assumptions around extended longevity as in UN pop projections. However within our projection model we scale-up health systems significantly faster than what would happen if business as usual continued. Thus the UN pop projections do not adequately capture the extent to which UHC is strengthened within our model.

Third, the estimated healthy life year gain is projected only through to 2030, whereas the health impact of the many preventive actions implemented within the care package will only become apparent beyond the 15 year time period modelled.

At the same time, some of the underlying increase in general longevity as projected in the UN pop datasets would capture the conditions that we are explicitly modelling, without the increased intervention coverage but capturing the forthcoming impacts of preventive interventions. Thus, there is a chance of overestimation for the estimates for LE_S. We therefore consider the scenario “S” estimates to be a conservative, minimum measurement of life expectancy gain, with the comparisons to baseline, “B”, an optimistic estimation of future gains.

4.8 Overall limitations of the SDG cost and impact projections

The main limitation of the modelling approach used is its scope, insofar that it limits what activities and health services to include within our resource estimates. We relied on existing models and treatment protocols to model resource needs, including only interventions whose effectiveness has been demonstrated, for which there is a general consensus on their appropriateness for inclusion in discussions around UHC, and for which there is reasonable information about current country specific coverage levels. However, there are interventions which would be important to include within a country-level comprehensive package of services and for which no available model could be identified, such as for interventions to reduce suicide, or treatment of cancers not included in our analysis (e.g, oral cancer, child leukaemia) or hepatitis. Other areas important for public health which we were not able to address include assistive technologies and oral health, given little information about current coverage levels.

In addition to health sector interventions, we have modelled out resource needs for additional areas such as WASH and indoor air pollution. However, these investments represent only a selected part of the overall multisectoral needs for health. In terms of reducing mortality and overall improvement in health, important gaps within our analysis include addressing road traffic injuries, as well as other injuries, including those linked to violence and crime, limiting exposure to chemicals, and reducing violence against women.

Due to overall uncertainty in relation to predicting what the future will bring, we do not model changes in technology over time, with a few exceptions where we adapt the projections to allow for more efficient behaviours. This includes, for example, the modelling of family planning services, where we model a shift towards modern methods within the scale-up of general contraceptive prevalence. Another example is within the cold chain modelling, where the model assumes a shift towards renewable energy sources (i.e., solar panels) over time.

We do not have data on actual cost structures in countries. Therefore, our approach of adding the incremental per capita need on top of the total current spending is an approach with many limitations. For example, health worker salaries constitute a large share of the resource need. However, while our model projects the salaries that health workers will receive by country, the actual wage bill structure in country may be different.

In terms of addressing the knowledge gap on current cost structures, the technical review meeting provided an opportunity for validating prices of cost drivers. The validation process focused on the components within the model that drive the cost and impact projections, and inputs were provided from participating countries.[48]

[48] Representatives from Bangladesh, Brazil, China, Egypt, Ethiopia, India, Indonesia, Iran, Mexico, Myanmar, Nigeria, Pakistan, the Philippines, and Viet Nam were present.

Section 5. Methods for projecting available financing

5.1 Outputs

The projections developed cover the period from 2016 to 2030 and concern three health expenditure aggregates:

1. Total health expenditure (THE)
2. Domestic total health expenditure (D-THE)
3. General government health expenditure (GGHE)

Simple and transparent estimation methods (detailed below) were adopted to allow a common approach for all countries using only universally available inputs such as gross domestic product forecasts and population projections. Range estimates have been favoured over point estimates because of the inherent uncertainty associated with long-term projections.

5.2 Inputs

The key variables and their sources used in the projections are shown in Table S10.

Table S12: Sources for assumptions on baseline and 2030 targets within our model

Acronym	Variable	Source
THE	Total health expenditure	WHO Global Health Expenditure Database
D-THE	Domestic total health expenditure	WHO Global Health Expenditure Database
GGHE	General government health expenditure	WHO Global Health Expenditure Database
POP	Population	UN World Population Prospects, 2015 Revision, Total Population Medium Fertility Variant
GGE	General government expenditure	IMF, World Economic Outlook, April 2016
GDP	Gross domestic product	IMF, World Economic Outlook, April 2016

The health expenditure input data is based on financing agents consistent with the System of Health Accounts (SHA 1.0). It includes both current and capital health expenditure. General government health expenditure includes social health insurance as well as foreign development assistance for health channelled through government as budget support. It is assumed that these external resources are progressively replaced by domestic resources.

Due to the absence of a dependable source with a complete set of GDP projections to 2030 for all WHO member countries, the non-parametric method of bootstrapping was used to obtain GDP growth estimates beyond the IMF's projections to 2021 (further details provided below).

5.3 Units of Measurement

All amounts are measured in constant 2014 USD. Exchange rates are held constant for future periods based on 2014 annualised exchange rates. All aggregated results are calculated as unweighted simple averages unless otherwise indicated.

5.4 Scenarios

We consider 4 scenarios: flatline, business as usual, moderate progress, and optimistic.

5.5. Estimation Methods

5.5.1 Total health expenditure (THE)

Total health expenditure, which includes, among other components, government health expenditure, social health insurance, voluntary private health insurance, out-of-pocket spending, and aid, provides the overall envelope of available resources for health in a country. Although THE and its per capita amount by themselves reveal little about the quality, efficiency and equity of a country's health care system, THE gives insights into potential levels of attainable health care given the available resources. This is particularly important when it comes to meeting essential health needs in severely resource constrained environments such as in conflict settings and low income countries.

Total health expenditure for each country for years 2015-2030 in each scenario is given by

$$THE_{c,t} = \left[\left(\frac{THE}{GDP}\right)_{c,\ t-1} \times f(\delta_c) \right] \times GDP_{c,t}$$

where

- c is the country and t is the year
- GDP is gross domestic product based on IMF-WHO projections
- THE is total health expenditure
- $f(\delta_c)$ is a health-economy expansion function

This top-down approach gives THE as a function of just GDP and the share of health expenditure to the total economy. Our flatline scenario golds the last 5-year average of THE as a share of GDP constant whereas our business as usual scenario alters health's share of the economy based on country specific historical trends given by linear regression of observed health expenditure data from 1995 to 2014. The moderate progress and optimistic scenarios are based on normative increases in THE as a share of GDP that would constitute a favourable expansion of available resources for health, specifically, a 1% point increase over 2015 to 2030 under the moderate scenario and a 2% point increase under the optimistic scenario (e.g. THE as % of GDP increases from an initial 3% to 4% under the moderate scenario and to 5% under the optimistic scenario. See also the section on scenarios below).

Although the relationship between health expenditure and economic development has been studied extensively, it should be noted that the above health-economy expansion function is not an elasticity. Without a more complex model and the availability of projections of other predictive variables, the use of an elasticity is not possible.

Figure S6. THE% GDP and GDP per capita in 2014, selected countries with population > 500,000

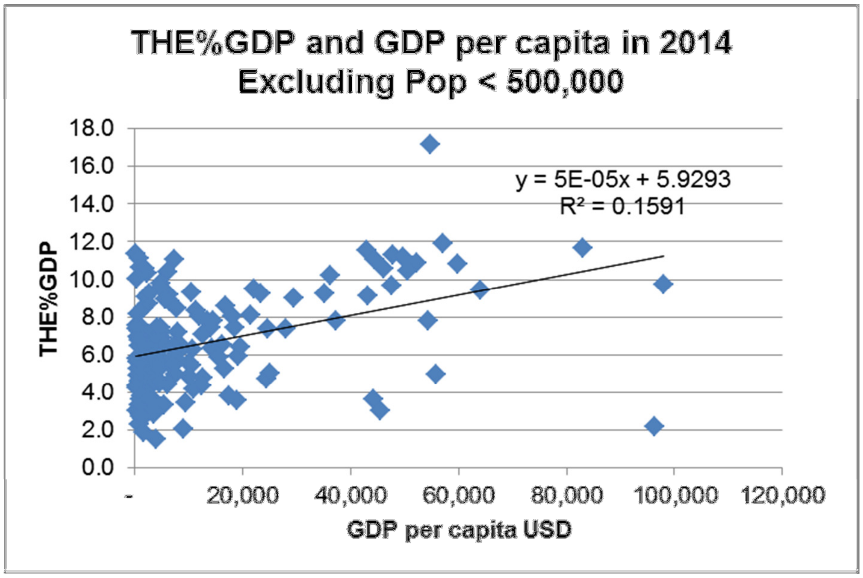

As illustrated in the scatterplot diagram above, the direct relationship between GDP and THE as a share of GDP per capita is weak in part due to the presence of aid in low and middle income countries. The assumed expansion of THE as a share of GDP under the progress and ambitious scenarios is nevertheless consistent with theoretical literature and observed historical trends.

5.5.2 Domestic total health expenditure (D-THE)

Domestic total health expenditure, which is equal to total health expenditure minus external resources as a source, provides an additional perspective on available resources and is a measure constructed exclusively for this exercise.

Domestic total health expenditure for each country for years 2015-2030 in each scenario is given by

$$D\text{-}THE_{c,t} = \left[\left(\frac{D\text{-}THE}{GDP}\right)_{c,\ t-1} \times f(\gamma_c) \right] \times GDP_{c,t}$$

where

- c is the country and t is the year
- GDP is gross domestic product based on IMF-WHO projections
- D-THE is domestic total health expenditure

- $f(\gamma_c)$ is a health-economy expansion function

As for total health expenditure discussed above, the projections of D-THE are based on a top-down approach and consier the same equivalent scenarios as THE (see the scenario-specific analysis below for the specific parameters).

5.5.3 General government health expenditure (GGHE)

General government health expenditure, which also encompasses social health insurance, represents a large part of total health expenditure in most countries and plays a central role in advancing universal health coverage by reducing financial barriers and impoverishment through prepayment and pooling. Projections of GGHE can therefore provide some general insights into both the quantity and quality of future health spending. This emphasis on government spending is also consistent with the Addis Ababa Action Agenda, which focuses on the need to raise domestic resources.

Government health expenditure for each country for years 2015-2030 in each scenario is given by

$$GGHE_{c,t} = \left[\left[\left(\left(\frac{GGE}{GDP}\right)_{c,\ t-1} \times f(\alpha_c)\right)\right] \times \left[\left(\left(\frac{GGHE}{GGE}\right)_{c,\ t-1} \times f(\beta_c)\right)\right]\right] \times GDP_{c,t}$$

where

- c is the country and t is the year
- GDP is gross domestic product based on IMF-WHO projections
- GGE is general government expenditure
- GGHE is general government health expenditure
- $f(\alpha_c)$ is a fiscal space expansion function
- $f(\beta_c)$ is a health prioritisation function

This bottom-up approach gives GGHE as a function of fiscal space, health prioritisation and GDP. Our different scenarios are established by altering the fiscal space and health prioritisation variables (α and β) based on different assumptions and analyses of historical data. Essentially, alpha determines by how much and how quickly government revenues increase and beta determines how health's share of the total government expenditure evolves. The product of the fiscal space and health prioritisation gives the often discussed share of GGHE to GDP.

$$\frac{GGHE}{GDP} = \uparrow \frac{GGE}{GDP} \times \frac{GGHE}{GGE} \uparrow$$

In our flatline, business as usual, and moderate progress scenarios, historical values and trends were projected forward to establish variations of what might be considered the range of probable future available resources. Under the flatline scenario, the 5-year average values for fiscal space and health prioritisation were held constant. Under the business as usual scenario, a linear regression of observed health expenditure data from 1995 to 2014 was used to establish the country specific parameters of alpha and beta. If the historical trend

growth in fiscal space and health prioritisation was not statistically significant, the last values for fiscal space and health prioritisation were held constant. The moderate progress scenario assumes certain improvements on top of the continuation of historical trends and our optimistic scenario sets normative levels that would mark a dramatic and significant positive change given different starting points (see table below and summary in section on scenarios). The possibility that the business as usual and moderate scenarios, which are based on country-specific historical trends, could exceed the normative levels of the optimistic scenario was not ruled out.

Table S13: Assumptions on increases in allocation towards health

GGE%GDP<br>Fiscal Space	GGHE%GGE<br>Health Prioritisation
If <20% in 2014 increase to 25% by 2030	If <7% in 2014 increase to 10% by 2030
If >20% and <40% in 2014, increase of 5% (max 40%) by 2030	If >7% and <13% increase by 3% (max 15%) by 2030
If >=40% in 2014, flatline (i.e. hold ratio constant)	If >=13% in 2014 make 15% in 2030 (N.B. If >15% in 2014 reduce to 15%)

5.5.4 Gross domestic product (GDP)

Gross domestic product is a readily available key measure of economic development and a central variable to the above outlined methods of projecting health expenditure. Despite the existence of GDP forecasts for many countries, a dependable source with a complete set of GDP growth projections for all WHO Member Countries out to 2030 does not exist, as far as the authors are aware. Given the considerable uncertainty and potentially arbitrary nature of GDP predictions beyond 5-10 years, a simple, defensible method to get range estimates for all countries was required.

On the advice of counterparts from the World Bank (WB) and the International Monetary Fund (IMF), we prepared GDP growth projections for 2022 to 2030 with lower and upper ranges using the IMF's projections to 2021 and each country's historical data. The non-parametric method of bootstrapping to obtain expected average growth rates with a lower and upper bound for years 2022-2030 was adopted for its simplicity and its non-reliance on statistical assumptions about the normality of the data. The bootstrap was based on 1000 sample replications (draw and replacement) and greater weighting was placed on more recent years (2011-2021) assuming that current growth patterns would have more influence on future growth out to 2030. Specifically, the

$$w_i = i^{th}/\sum_{i=1}^{26} i \quad \rightarrow \quad 1995{:}\, w_1 = \frac{1}{351} = 0.00285;\ 1996{:}\ w_2 = \frac{2}{351} = 0.0057$$

where

- W are weights of sampling probabilities (e.g. weight for 1995 observations = 0.2%, for 1996 =0.6% and so on)

A sensitivity analysis comparing the GDP growth rates obtained using the Bootstrap method with available GDP growth rates from other sources showed our projected range estimates to be consistent and robust.

Where GDP forecasts to 2030 were available from an official institution of a country, such as the national central bank, these were used in place of IMF-WHO projections.

5.6 *Scenario-specific analysis*

5.6.1 *Scenario Parameters*

Four scenarios were modelled to establish the available resources that might be potentially available with different priorities and financing policies.

1. Flatline – expenditure ratios remain unchanged from their current values
2. Business as usual – historical trends in expenditure growth continue
3. Moderate progress – positive trends accelerate and increase notably
4. Optimistic – dramatic and significant positive changes in health expenditure

As outlined below, these scenarios were modelled slightly differently for the bottom-up approach used to estimate GGHE compared with the top-down approach used for the THE and D-THE envelopes.

Table S14: Assumptions

**Assumptions for GGHE estimates**				
	GGE%GDP<br>Fiscal Space	GGHE%GGE<br>Health Prioritisation	GDP	Population
Flatline	Average value 2010-2014 held constant		IMF-WHO Projections<br>:<br>Low, Medium & High Growth Variants	UN Population Projections : Medium Fertility Variant
Business as Usual	Change based on country specific historical trends given by ordinary least squares over 1995-2014			
Moderate progress (variant a) – "double effort"	Change based on double the magnitude of country specific historical trends given by ordinary least squares over 1995-2014. If trends are in negative direction, average trend of income group adopted.			
Moderate progress (variant b) – "best performer"	Change based on best performer within income group over 1995-2014. Value for largest fiscal space expansion and health reprioritisation may come from different countries.			
Optimistic	• If <20% in 2014, increase to 25% by 2030<br>• If >20% and <40% in 2014, increase of 5% (max 40%) by 2030<br>• If >=40% in 2014, flatline (i.e. hold ratio constant)	• If <7% in 2014, increase to 10% by 2030<br>• If >7% and <13% in 2014, increase by 3% (max 15%) by 2030<br>• If >=13% in 2014 make 15% in 2030 (N.B. If >15% in 2014 reduce to 15%)		

**Assumptions for THE and D-THE**			
	THE%GDP<br>Health-Economy	GDP	Population
Flatline	Average value 2010-2014 held constant	IMF-WHO Projections<br>:<br>Low, Medium & High Growth Variants	UN Population Projections : Medium Fertility Variant
Business as Usual	Change based on country specific historical trends given by ordinary least squares over 1995-2014		
Moderate progress	Increase of 1%point by 2030 (e.g. 3% to 4%)		
Optimistic	Increase of 2%points by 2030 (e.g. 3% to 5%)		

5.6.2 *Scale-up curves for financial projections*

Rather than adopting a linear growth pathway, the change in the annual increase of available resources (i.e. scale-up) is determined by each country's categorisation. For conflict and the first subset of foundation countries, the expansion of available resources is delayed and then accelerates in later years consistent with the fact these countries need to rebuild and/or strengthen their health system foundations. HS3countries, in contrast, should in

principle be able to raise and absorb additional resources rapidly with their more developed health systems and better governance structures. Hence, the scale-up curve for transformation countries assumes early rapid growth before tapering (i.e. front loading). HS1 and HS2 countries, which possess by definition health systems in between those of HS3and conflict countries, exhibit a smoother and steadier growth pathway over the entire period. The scale-up curves used for these country groups are the same as those used in the SDG costing exercise (see figure S3).

5.6.3 Limitations of methods used to project available financing

One of the first limitations of the projections relates to the available historical data that combines current and capital health expenditure, which should ideally be analysed and projected separately. Another important limitation of the baseline data is the inclusion of on-budget foreign aid under general government health expenditure. This is due to the aggregated reporting of expenditure by financing agents. This means that for some countries, a certain amount of GGHE is actually from external sources, which complicates the discussion of domestic revenue-raising. It is assumed as part of this study that this amount is progressively replaced by truly domestic public revenue.

Next, the top-down projections of THE give only the size of the health resources envelope and not its breakdown. The bottom-up estimations of GGHE are to also be treated separately and not combined with the projections of THE. This study does not attempt to estimate other disaggregated values of THE, such as voluntary health insurance or out-of-pocket spending, which will be influenced by future health financing reforms among other factors. Hence, it is also not the purpose of this analysis to consider future changes to the shares of health expenditure in relation to THE or GGHE.

Another challenge is the uncertainty of future aid flows and allocations, which are highly variable and politically determined. This study does not provide estimates of future aid even though external resources are likely to continue to be an important source of revenue for many countries, especially those in the conflict and foundation groups.

Finally, it should be noted that the analysis relies on GDP growth projections that are inherently difficult to predict far into the future. To mitigate this, the study uses the best available forecasts from the IMF then builds upon these by constructing a range of average expected growth rates although this implicitly assumes the continuation of historical long-term growth.

Section 6. Review processes

This work was guided by regular consultation and review processes. A WHO and UNAIDS expert group met monthly to provide inputs on the methodological framework, scope of analysis, and modelling approach. The group included representatives from individual disease areas and health system building blocks.

In July 2016, WHO organised an expert review and country feedback meeting to discuss the methodology and preliminary results of the analysis. Participants included international experts and academics, and representatives from 14 low and middle income countries jointly accounting for more than 75% of population covered in the analysis.[49]

Discussions considered the methodological approach, presentation of results and key messages. Moreover country participants reviewed country specific input assumptions and provided feedback on these. The main focus for country specific review was assumptions for cost drivers, as well as assumptions for health impact projections.

Participant feedback was discussed and informed a revision in methods, where there was agreement that methods could be improved, for example on costing of emergency risk management, as well as the projections of available health financing. Moreover, country feedback informed the revision of data within the models with regards to country specific data points such as the number of existing health workers, the salary cost of a nurse, current health service coverage for specific interventions, and epidemiology such as maternal mortality or the current prevalence of tobacco use. Feedback was only incorporated when references were provided.

Section 7. Efficiency considerations

In recognition of the fact that current health systems in low and middle income countries may not currently be operating at high levels of efficiency and there may be scope for improvement, we design scenarios around efficiency, the purpose of which is to demonstrate how expectations on efficiency would affect the estimated potential funding gap in countries. While expectations for zero wastage may be unrealistic, we consider scenarios that consider improving current system efficiencies which would effectively free up resources and lower overall projected costs. The converse argument is that weak capacity in low-income countries increases the costs of making improvements, and that current inefficiencies could be assumed to also be prevalent in future systems – at least for the short term - such that projected marginal costs should be higher.

7.1 Methods

Based on the World Health Report (WHR) 2010, we adopt specific assumptions around the share of health expenditure that is inefficiently used. We consider two alternative scenarios both for the Progress and Ambitious resource needs estimates, where one assumes a lower level of efficiency and another assumes a higher level of efficiency.

7.1.1 Less efficient scenario

The WHR2010 reported that 20-40% of resources in health systems are currently wasted. As such, actual implementation in country may incur additional resources beyond what we have modelled as our standard scenario, which assumes that incremental costs 2016-2030 reflect efficient practice. The five categories of inefficiencies considered in WHR2010 were human resources, medicines, hospitals, leakages, and the intervention mix. We consider our model to specify the requirements for human resources, hospitals, and

[49] Bangladesh, Brazil, China, Egypt, Ethiopia, India, Indonesia, Iran, Mexico, Myanmar, Nigeria, Pakistan, Philippines, and Viet Nam.

intervention mix rather rigorously. We therefore only model additional resource requirements due to potential inefficiency for two categories: medicines and leakages. As such, we apply a cost increase on the incremental cost projections for 2016-2030. For medicines we apply a constant ratio across all years (15%) for all countries, based on WHR2010, on the projected additional commodity cost. With respect to leakages, the inefficiency loss is estimated to range from 5-10% for LMICs, and we therefore apply a 10% mark-up on the estimated overall additional costs. The same assumptions are applied to the cost projections for the two scale-up scenarios (ambitious and progress).

7.1.2 More efficient scenario

For the more efficient scenario we consider that the incremental investments will reflect efficient practise (as in the standard scenario), and we make the adjustments for increasing efficiency on the current total health expenditure (THE) by country. We model a shift towards more effective practices over time such that the potential efficiency savings outlined in WHR2010 are reached by 2030, that is that 3% to 5% of THE could be saved due to increased efficiency in the procurement of medicines in low income countries, and 2% to 5% in middle income countries. We use the lower range for the Progress scenario, and the higher range for the Ambitious scenario. These are applied on the THE projections for 2016-2030 for each respective scenario (ambitious and progress). Efficiency is assumed to gradually increase over time in a linear fashion such that the high estimate is attained by 2030. Similarly for leakages, we assume a gradually more efficient system such that by 2030 an estimated 10% of THE is released for more productive purposes in the ambitious scenario, and similarly 5% of THE can be released in the progress scenario. This scenario is equivalent to expanding fiscal space by reorienting health systems towards more effective practises.

7.2 Results

Here we present results for low-income countries, which are the countries that have the greatest needs to reach SDG benchmarks. The purpose is to highlight how expectations on efficiency would affect the estimated potential funding gap for these countries

Table S15. Projected funding gap in 2030 (difference between projected costs and projected available funding), with inefficiency-efficiency range, Total for low income countries (N=28), by scenario, billion US$2014

	Ambitious scale-up, optimistic financing projections	Progress scale-up, moderate financing projections
Regular scenario (costs)	16	7
Less efficient scenario (costs)	20	10
Increasing efficiency scenario (funds released)	7	4

Figure S7. Projected funding gap in 2030 (difference between projected costs and projected available funding) in billions US$2014, Total for low income countries (N=28)

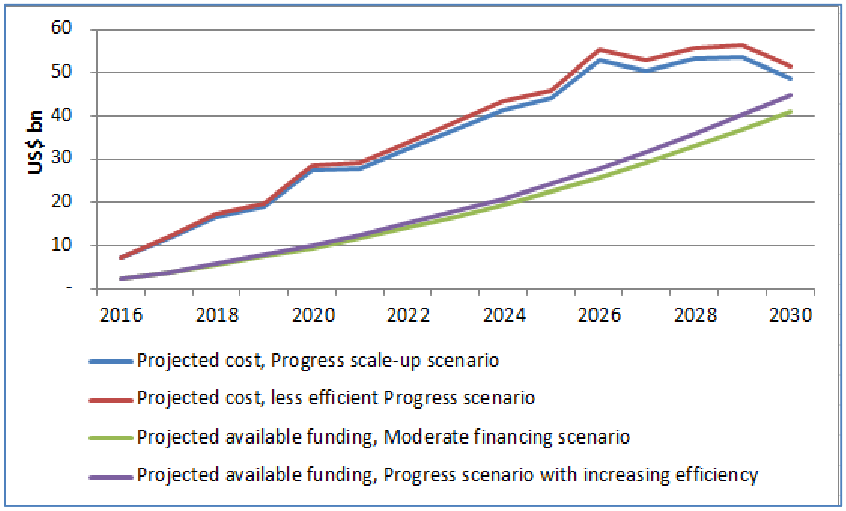

Figure S7A. Progress scale-up and moderate financing projections

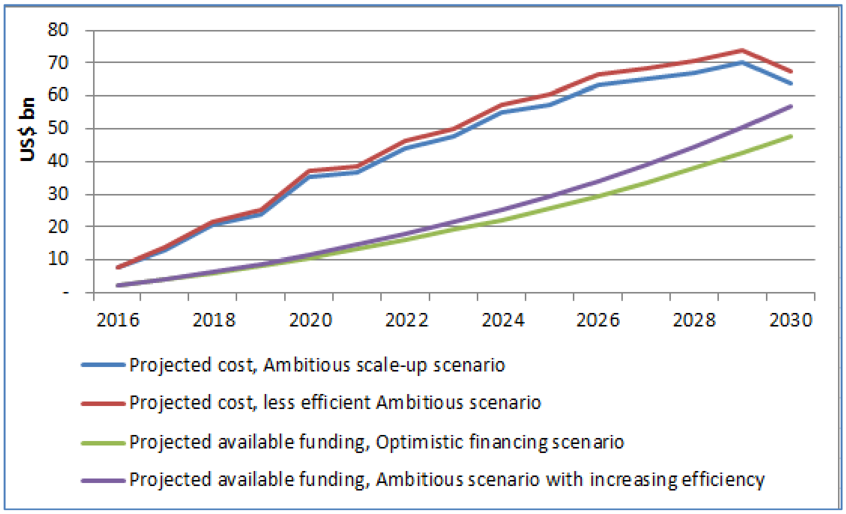

Figure S7B. Ambitious scale-up and optimistic financing projections

Section 8. Additional results, tables and figures

This section presents additional tables on projected investments and related costs.

8.1 Total additional cost by investment area

Table S16. Total Additional Costs, by investment area, 2016-2030, Billion USD 2014, Ambitious Scenario (67 countries total)

Investment area	2016	2017	2018	2019	2020	2021	2022	2023	2024	2025	2026	2027	2028	2029	2030
Infrastructure and Equipment	26.1	37.7	53.7	61.4	88.4	78.9	94.8	91.7	101.1	105.9	118.6	118.6	120.6	127.2	93.2
Health workforce	6.3	20.2	29.6	50.6	69.5	84.4	96.5	107.0	116.0	122.8	129.0	134.9	140.4	145.5	149.9
Health information systems	0.0	0.2	0.1	0.4	0.5	0.7	0.6	0.6	0.5	0.5	0.5	0.7	0.6	0.6	0.6
Supply chain	4.9	5.0	5.6	6.8	7.9	8.6	9.5	10.4	11.3	12.1	13.1	14.0	14.9	15.7	16.5
Health financing policy	0.0	0.1	0.2	0.3	0.5	0.6	0.8	0.9	1.1	1.3	1.5	1.8	2.0	2.3	2.6
Governance	1.4	1.6	1.6	1.6	1.7	1.8	1.7	1.8	1.8	1.8	1.9	1.8	1.8	1.8	1.8
Emergency Risk Management	0.0	0.0	0.3	0.3	0.4	0.8	1.1	1.3	1.4	1.5	1.6	1.7	1.9	2.0	2.1
Commodities and supplies	6.1	13.2	18.5	23.3	28.2	32.6	36.7	41.5	46.2	50.2	54.6	59.2	63.2	67.3	71.8
Emergency Relief	1.1	1.1	1.1	1.1	0.9	0.8	0.8	0.5	0.5	0.0	0.0	0.0	0.0	0.0	0.0
Reconstruction costs in conflict and fragile settings	0.0	0.2	0.3	1.1	2.0	1.6	0.0	0.0	0.0	0.0	0.0	0.0	0.0	0.0	0.0
Additional health programme costs*	10.8	14.9	17.3	19.5	22.4	21.7	22.2	23.9	25.5	31.3	27.4	28.7	29.2	26.8	28.7
**Total**	56.8	94.2	128.5	166.6	222.4	232.6	264.6	279.6	305.4	327.4	348.3	361.4	374.6	389.1	367.1

Table S17. Total Additional Costs, by investment area, 2016-2030, Billion USD 2014, Progress Scenario (67 countries total)

Investment area	2016	2017	2018	2019	2020	2021	2022	2023	2024	2025	2026	2027	2028	2029	2030
Infrastructure and Equipment	22.5	36.0	46.6	53.1	70.6	65.5	76.0	76.4	83.1	87.5	107.9	99.7	104.1	107.8	80.0
Health workforce	5.7	13.7	20.8	31.6	41.1	49.5	56.4	62.4	67.7	72.1	76.5	80.5	84.3	88.1	91.5
Health information systems	0.0	0.2	0.1	0.3	0.4	0.5	0.4	0.4	0.4	0.4	0.4	0.7	0.5	0.5	0.5
Supply chain	2.0	3.7	4.7	5.7	6.8	7.7	8.5	9.5	10.4	11.3	12.2	13.1	14.0	14.7	12.9
Health financing policy	0.0	0.0	0.1	0.1	0.2	0.2	0.3	0.4	0.5	0.5	0.6	0.8	0.9	1.0	1.1
Governance	1.4	1.4	1.4	1.4	1.5	1.6	1.5	1.5	1.5	1.5	1.6	1.5	1.5	1.5	1.5
Emergency Risk Management	0.0	0.0	0.2	0.3	0.4	0.5	0.6	0.8	0.7	0.8	0.8	0.9	0.9	1.0	1.0
Commodities and supplies	4.6	10.5	14.7	18.4	22.2	25.9	29.3	33.0	37.0	40.3	43.8	47.6	51.0	53.9	57.3
Emergency Relief	1.1	1.1	1.2	1.2	1.0	0.9	0.8	0.6	0.6	0.0	0.0	0.0	0.0	0.0	0.0
Reconstruction costs in conflict and fragile settings	0.0	0.2	0.3	1.1	2.0	1.6	0.0	0.0	0.0	0.0	0.0	0.0	0.0	0.0	0.0
Additional health programme costs*	9.0	12.1	12.9	14.6	17.1	16.4	16.8	18.5	19.7	25.4	21.3	22.5	22.9	21.7	22.4
Total	46.4	79.0	103.0	127.9	163.1	170.3	190.6	203.5	221.6	239.9	265.1	267.2	280.1	290.2	268.3

**"Additional health programme costs" include those that are programme-specific but do not refer to specific medicines, drugs or lab tests. This includes costs for programme-specific administration staff, supervision and monitoring relative to the services for which the programme provides leadership and oversight (e.g., the national malaria programme provides implementation guidance, monitors and supervises service delivery for malaria). It also includes mass media campaigns and demand generation.*

8.2 Strengthening key components of the health system

Table S18. Strengthening key components of the health system, 2016-2030

Country Groups	Total Health Facilities Built, 2016-2030	Number of Rural Health Centers Built 2016-2030	Number of Urban Health Centers Built 2016-2030	Number of Rural District Hospitals Built 2016-2030	Number of Urban District Hospitals Built 2016-2030	Number of Provincial Hospitals Built 2016-2030	Total Health Workers Added 2016-2030	Number of physicians added to the health workforce 2016-2030	Number of nurses and midwives added to the health workforce 2016-2030	Number of other cadres of health workers added to the health workforce 2016-2030
Ambitious scale-up scenario										
All	415,034	261,449	116,266	29,826	6803	690	23,567,016	3,014,527	10,480,173	10,072,316
Conflict-affected states	9,870	6,108	2,730	749	278	4	519,505	85,140	198,513	235,852
Vulnerable systems	34,814	24,775	5,997	3,072	829	141	1,699,054	258,214	703,005	737,834
HS1	53,959	40,522	6946	5,635	779	77	3,198,961	494,926	1,287,331	1,416,704
HS2	206,840	133,931	54,230	15,478	2738	463	13,770,370	1,837,014	5,692,770	6,240,585
HS3	109,551	56,113	46,364	4892	2178	4	4,379,127	339,233	2,598,554	1,441,340
Progress scale-up scenario										
All	377,948	244,020	108,117	20,540	4,776	494	14,244,625	1,882,100	6,086,331	6,276,194
Conflict-affected states	8,883	5,646	2,534	522	177	3	360,410	52,364	142,817	165,228
Vulnerable systems	31,490	22,991	5.671	2,095	635	97	944,007	130,828	346,496	466,683
HS1	49,544	38,394	6,656	3,890	546	58	1,545,949	215,361	565,285	765,303
HS2	189,605	125,810	50,823	10,737	1,903	331	8,391,114	1,133,926	3,346,060	3,911,128
HS3	98,426	51,179	42,432	3,295	1,515	5	3,003,145	349,620	1,685,674	967,851

8.3 Scenarios of Additional Resource Needs vs. Additional projected Financing

Figure S8. Scenarios of Additional Resource Needs vs. Additional projected Financing, US$ 2014 billion, HS1 Countries (N=26)

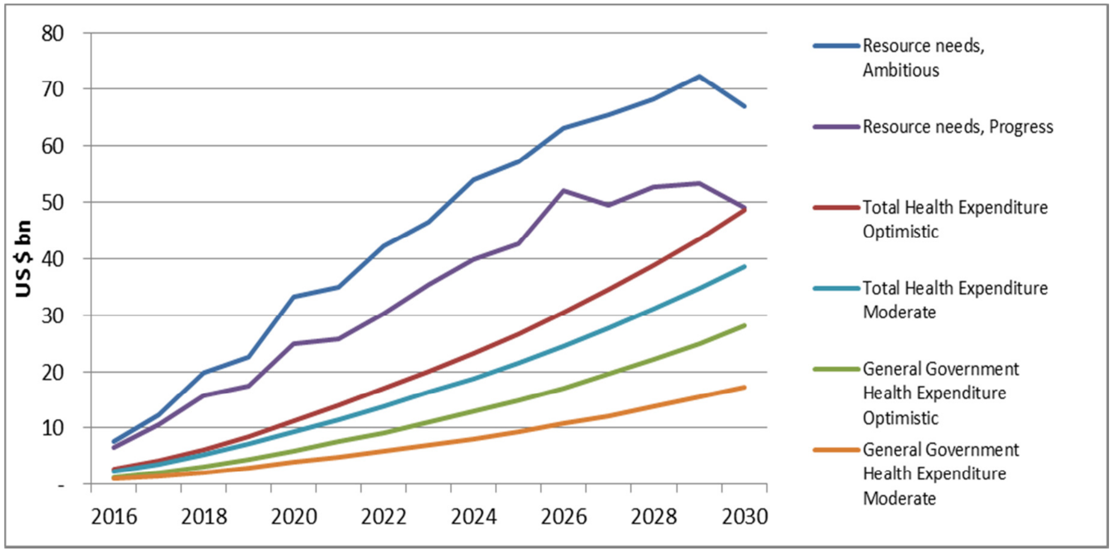

Figure S9. Scenarios of Additional Resource Needs vs. Additional projected Financing, US$ 2014 billion, HS2 Countries (N=16)

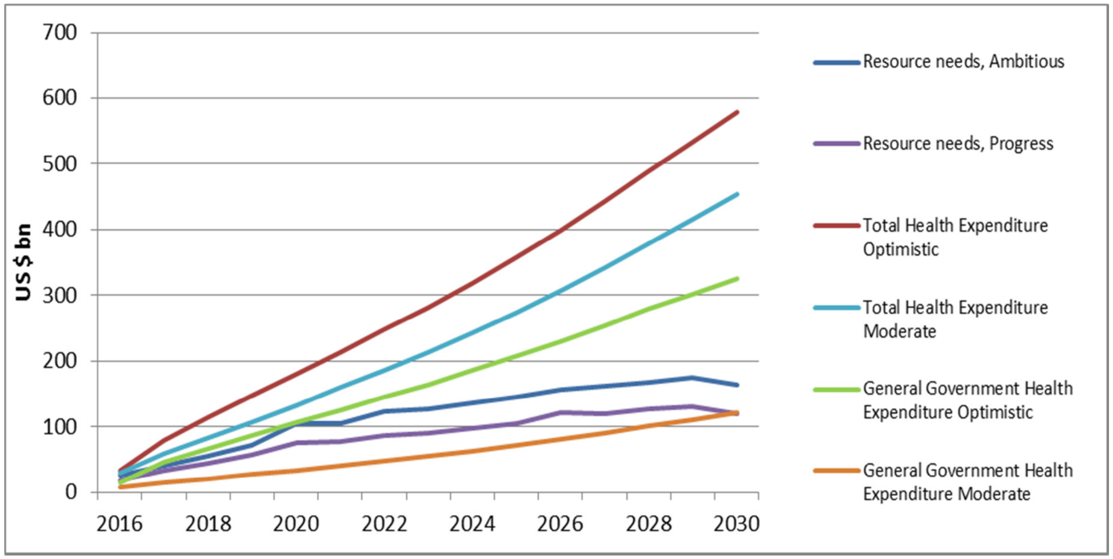

Figure S10. Estimated incremental resource needs and projected additional available financing in year 2030, average per capita and per country group (US$ 2014)

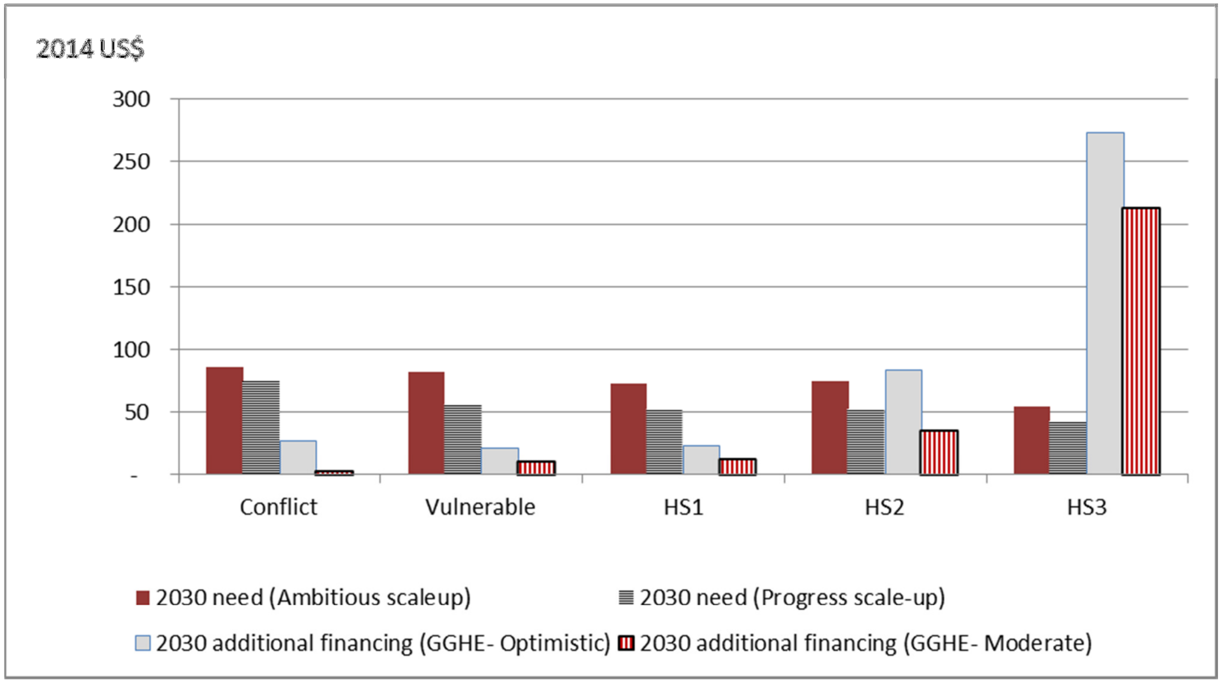

*8.4 Estimated Required Health Expenditure, as percentage (%) of projected GDP**

Table S19. Estimated Required Health Expenditure, as percentage (%) of projected GDP*

Country groups	N	Total Health Expenditure (as % of GDP) 2014			Total Projected Additional Resource Needs (as % of GDP), 2030			Total Projected Required Health Expenditure ** (as % of GDP) 2030		
Progress scenario		Average	Min	Max	Average	Min	Max	Average	Min	Max
All 67	67	5.6%	2.2%	10.8%	3.5%	0.1%	18.7%	6.5%	1.9%	22.6%
Conflict-affected states (C)	4	4.1%	2.7%	4.8%	9.3%	1.3%	18.7%	12.6%	4.5%	22.6%
Vulnerable systems (V)	11	6.8%	3.6%	10.8%	6.1%	3.4%	10.6%	9.5%	5.5%	12.9%
HS1	15	5.5%	2.2%	8.0%	6.0%	1.5%	12.3%	8.6%	2.1%	16.5%
HS2	16	4.8%	2.7%	7.5%	1.5%	0.5%	3.5%	3.6%	1.9%	5.9%
HS3	21	6.2%	4.0%	9.1%	0.4%	0.1%	0.9%	4.3%	2.1%	8.9%
Ambitious scenario		Average	Min	Max	Average	Min	Max	Average	Min	Max
All 67	67	5.6%	2.2%	10.8%	4.6%	0.2%	17.9%	7.5%	2.1%	20.5%
Conflict-affected states (C)	4	4.1%	2.7%	4.8%	9.7%	1.6%	17.9%	13.0%	4.7%	20.5%
Vulnerable systems (V)	11	6.8%	3.6%	10.8%	8.5%	5.0%	14.2%	11.9%	7.2%	16.2%
HS1	15	5.5%	2.2%	8.0%	8.0%	1.9%	16.3%	10.7%	2.5%	20.5%
HS2	16	4.8%	2.7%	7.5%	2.0%	0.5%	4.4%	4.0%	2.1%	6.7%
HS3	21	6.2%	4.0%	9.1%	0.5%	0.2%	1.1%	4.4%	2.1%	9.2%

*Average/Min/Max refers to the average (mean), lowest and highest country values within each group. ** Total required expenditure estimated as Total Health Expenditure (THE) in 2014 plus the additional projected resource needs. There are two scenarios for projected THE: one moderate and one optimistic scenario. In this table the Progress scenario costs are compared with the Moderate scenario projected THE, and the Ambitious scenario costs are compared against the Optimistic scenario projected THE; which is why the total projected required THE as a share of GDP is higher for some countries in the Progress scenario than in the Ambitious scenario.

8.5 Resource needs by service delivery platform

We map the resource requirements to the four service delivery platforms. Intervention-specific costs such as commodities and supplies are directly associated with a specific platform. The allocation of health workforce costs by platform is based on bottom-up estimations of required full –time equivalent health workers per intervention, year and country from the OneHealth Tool simulations. Costs related to infrastructure are only included under platforms 3 (health centres) and 4 (hospitals). Overarching functions such as those related to governance, financial administration and emergency preparedness are presented separately.

More than half of additional resources will be required to support service delivery through first level clinical services. This is where the majority of investments in health workforce and infrastructure will be required to ensure that primary level quality care is accessible. This is also the platform to which most health interventions have been mapped, both preventive and curative (see section 2.2). Investments in specialized care entail setting up and running district hospitals to provide referral care.

Figure S11. Additional resource needs by service delivery platform (Ambitious scale-up scenario, 67 countries, 2030)

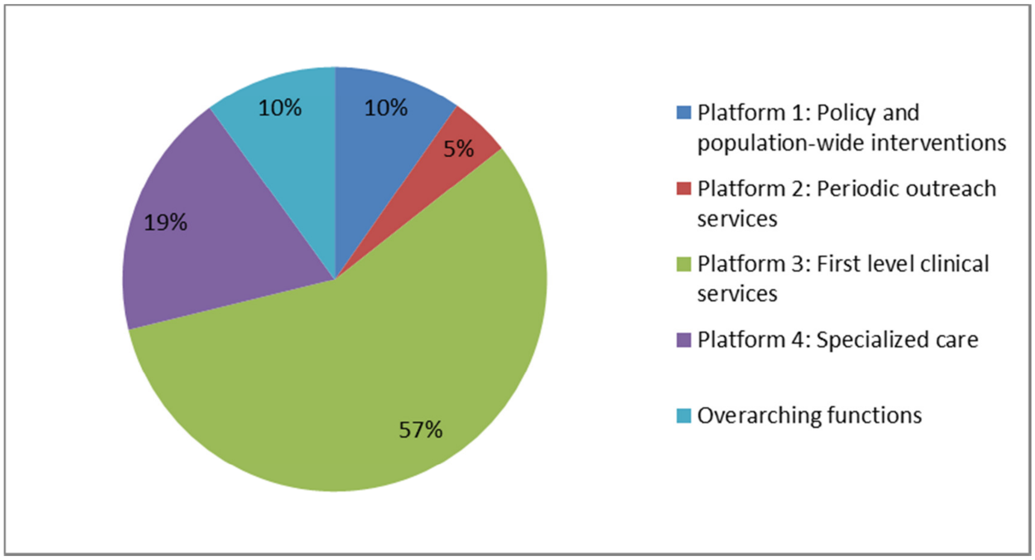

Table S20 illustrates the investment profile across country typologies. Primary level care will require the bulk of additional resources across all settings. The greatest relative investment in referral care is required in vulnerable and low income (HS1) countries where infrastructure investments have been overlooked.

Table S20. Additional resource needs by service delivery platform, Ambitious scale-up scenario, total by country typology, year 2030

	Costs included	Conflict	Vulnerable	HS1	HS2	HS3
**Share of costs, per service delivery platform**						
Platform 1: Policy and population-wide interventions	Commodities specific to interventions included under platform 1, a share of health worker time allocated to platform 1, Policy interventions aimed at changing behaviour	6%	6%	8%	8%	13%
Platform 2: Periodic schedulable and outreach services	Commodities specific to interventions included under platform 2, a share of health worker time allocated to platform 2, outreach activities to vulnerable groups	9%	7%	5%	6%	3%
Platform 3: First level clinical services	Commodities specific to interventions included under platform 3, a share of health worker time allocated to platform 3; Constructing, equipping and running health centres	52%	51%	51%	57%	60%

Platform 4: Specialized care	Commodities specific to interventions included under platform 4, a share of health worker time allocated to platform 4; Constructing, equipping and running hospitals; conditional cash transfers for facility deliveries.	22%	27%	28%	20%	13%
Overarching functions	All costs related to Supply chain, Governance, Health financing, Emergency preparedness, and Health Information Systems; Overall programme management.	11%	9%	9%	10%	11%
**Affordability of supporting overarching functions* with platforms 1 and 2**						
Number of countries for which estimated additional costs in 2030 (ambitious scenario) exceed projected additional available total health expenditure in 2030 (optimistic scenario)		1	0	0	0	0
Number of countries for which estimated additional costs in 2030 (ambitious scenario) exceed projected additional available general government health expenditure in 2030 (optimistic scenario)		2	2	2	0	0

**This includes the overarching costs of governance, health financing policy, health information systems, a share of supply chain costs, and overall programme management, in addition to specific activities and commodities associated with platforms 1 and 2.*

In settings where clinical services are still underdeveloped and health workforce density is low, there is still potential for rapidly moving towards full coverage with those interventions that can be delivered through the first two platforms: policy, population-wide, and periodic schedulable and outreach delivery. A comparison of the projected costs with the estimates additional available total health expenditure in 2030 reveals that only one (conflict affected) country may not be able to afford universal provision of interventions provided through these platforms. A comparison of costs with projected additional government health expenditure indicates that 61 out of 67 countries should be able to fully fund these interventions through government generated revenue streams.

8.6 Breakdown of disease specific resource needs

Figure S12 and Table S21 present a breakdown of projected costs that are specific to each disease control/ prevention programme area. These estimates include commodities specific to each intervention as well as additional programmatic interventions such as in-service training, outreach and monitoring activities.

Figure S12. Additional investments in specific disease control/prevention programmes, US$ 2014 billion, Ambitious scenario (all 67 countries)

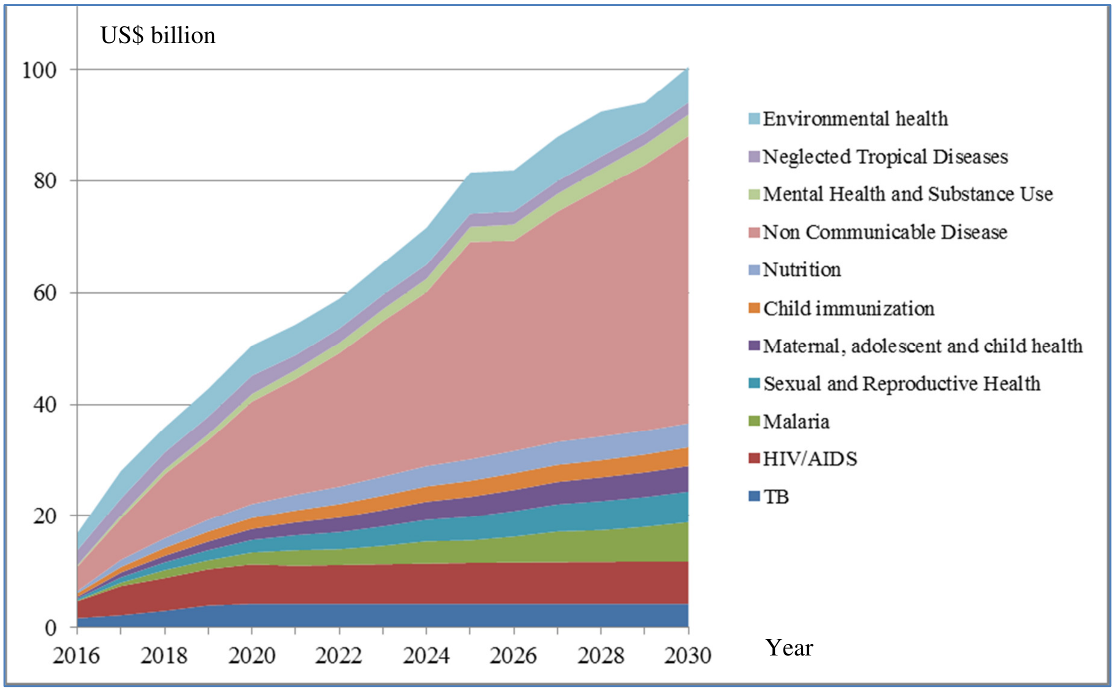

Table S21. Additional disease prevention and control /programme-specific costs 2016-2030, by programme area, US$ 2014 billion (all 67 countries), Ambitious scale-up scenario

Programme area	US$ bn 2016-2030	Percentage share
TB	57	6%
HIV/AIDS	102	11%
Malaria	51	5%
Sexual and reproductive health	50	5%
Maternal, adolescent and child health	42	4%
Child immunization	35	4%
Nutrition	46	5%
Non communicable disease (including cancer)	421	44%
Mental health and substance use	31	3%
Neglected tropical diseases	40	4%
Environmental health	89	9%
**Total**	**963**	**100%**

Numbers may not sum to the total because of rounding

References for supplementary material

Deogan C, Ferguson J, Stenberg K. Resource needs for adolescent friendly health services: estimates for 74 low- and middle-income countries. PLoS One. 2012;7(12):e51420. doi: 10.1371/journal.pone.0051420. Epub 2012 Dec 27.

De Vlas SJ, Stolk WA, le Rutte EA, et al. Concerted efforts to control or eliminate neglected tropical diseases: how much healthwill be gained? PLoS Negl Trop Dis 2016; 10: e000438.

Fund for Peace, Fragile states index 2016. http://fsi.fundforpeace.org/

Jamison DT, Shahid-Salles SA, Jamison J, et al. Incorporating Deaths Near the Time of Birth into Estimates of the Global Burden of Disease. In: Lopez AD, Mathers CD, Ezzati M, et al., editors. Global Burden of Disease and Risk Factors. Washington (DC): The International Bank for Reconstruction and Development / The World Bank; 2006. Chapter 6. Available from: https://www.ncbi.nlm.nih.gov/books/NBK11805/ Co-published by Oxford University Press, New York.

Menzies NA, Gomez GB, Bozzani F, et al. (2016). Cost-effectiveness and resource implications of aggressive action on TB in China, India and South Africa: a combined analysis of nine models. *Lancet Global Health*. 4 (11), e816-e826. doi.org/10.1016/S2214-109X(16)30265-0.

Phillips, J., & Millum, J. (2015). Valuing Stillbirths. Bioethics, 29(6), 413–423. http://doi.org/10.1111/bioe.12120

Portnoy A, Ozawa S, Grewal S, et al., 2015. Costs of vaccine programs across 94 low- and middle-incomecountries. *Vaccine*. 2015 May 7;33 Suppl 1:A99-108. doi: 10.1016/j.vaccine.2014.12.037.

Qureshi, Z. U., Millum, J., Blencowe, et al. (2015). Stillbirth should be given greater priority on the global health agenda. The BMJ, 351, h4620. http://doi.org/10.1136/bmj.h4620

Salomon JA, Vos T, Hogan DR, et al. Common values in assessing health outcomes from disease and injury: disability weights measurement study for the Global burden of Disease Study 2010. 2012 The Lancet 380(9859): 2129-2143.

Stop TB partnership (2015), Global Plan to End TB: the paradigm shift 2016-2020. http://www.stoptb.org/global/plan/plan2/

Stover J, Bollinger L, Izazola JA, et al. (2016) What Is Required to End the AIDS Epidemic as a Public Health Threat by 2030? The Cost and Impact of the Fast-Track Approach. *PLoS ONE* 11(5): e0154893. doi:10.1371/journal.pone.0154893

WHO (2010). The World Health Report. Health systems financing: the path to universal coverage.

WHO (2015), Global Technical Strategy for Malaria 2016–2030.

WHO (2015), Tracking universal health coverage: first global monitoring report.

WHO (2015), Investing to overcome the global impact of neglected tropical diseases.

WHO Global Health Observatory. http://www.who.int/gho/en/ Accessed 24 May 2016.

WHO (2016), FIT – Foundations, Institutions, Transformation. Information Brochure, Department of Health Systems Governance and Financing.

World Bank (2004). World Development Report 2004. Making Services Work for Poor People.

United Nations, Department of Economic and Social Affairs, Population Division, World Population Prospects: The 2012 Revision, New York, 2013
